# Aqueous glyoxal: a versatile synthon in heterocyclic synthesis

**DOI:** 10.1039/d5ra08496b

**Published:** 2026-02-19

**Authors:** Abolfazl Olyaei, Mahdieh Sadeghpour

**Affiliations:** a Department of Chemistry, Faculty of Science, Imam Khomeini International University Qazvin Iran; b Department of Chemistry, Qa.C., Islamic Azad University Qazvin Iran mahdieh.sadeghpour@iau.ac.ir

## Abstract

Glyoxal is a versatile, non-volatile compound with a wide range of applications, attracting significant interest from both researchers and various industries. It can be synthesized by numerous methods and is potentially derivable from natural sources. Moreover, glyoxal forms various hydrates and oligomers (*e.g.*, dimers and trimers). Its high reactivity allows it to undergo addition, condensation, and crosslinking reactions with a wide range of compounds, including alcohols, amines, aldehydes, carboxylic acids, cellulose, polyvinyl alcohol, and urea. This review discusses the applications of aqueous glyoxal as a versatile reagent in organic synthesis, with a focus on protocols for generating diverse molecular frameworks, including five- and six-membered heterocycles, aromatic compounds, fused heterocycles, polyaza polycyclic compounds, polyoxa polycyclic compounds and polyaza–polyoxa polycyclic compounds. The employed strategies include one-pot, multicomponent reactions and sequential methods, which use different catalysts under various conditions.

## Abstract 2

This review highlights the applications of aqueous glyoxal as a versatile reagent in organic synthesis, with a focus on protocols for generating diverse molecular frameworks, including five- and six-membered heterocycles.

## Graphical abstract

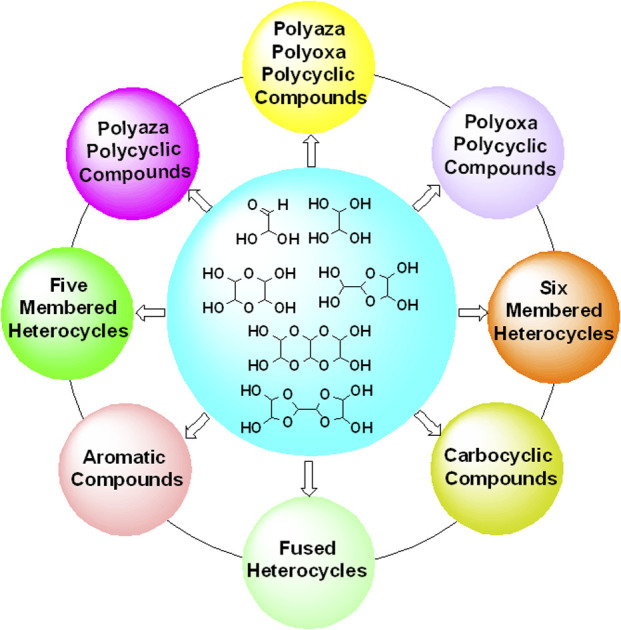

## 1. Introduction

**Fig. 1:**
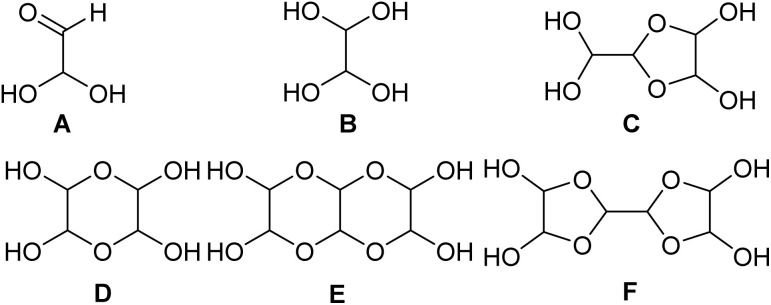
Different structures of aqueous glyoxal (A–F).

**Fig. 2:**
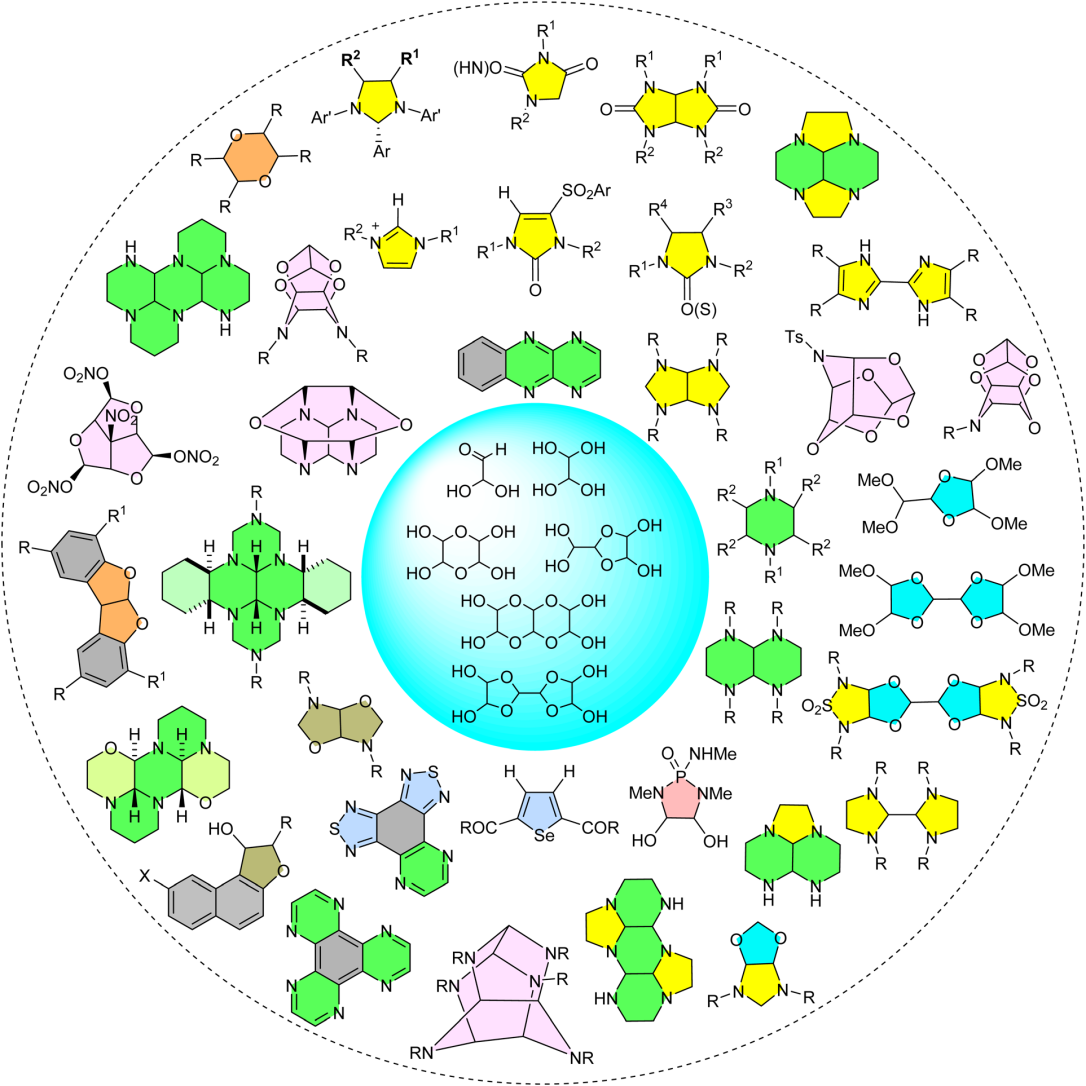
Glyoxal-based pathways for heterocycle synthesis.

Glyoxal is a versatile compound that has attracted the attention of researchers and various industries due to its wide range of applications. Glyoxal, an organic compound with the chemical formula C_2_H_2_O_2_ or O=CH–CH=O, is recognized as the simplest colored organic compound in its monomeric form. The glyoxal monomer is a yellow crystalline substance that transforms into a green liquid upon melting at 15 °C and boils at 50 °C, producing a green gas. The monomer remains stable for only a few hours. Even at 0 °C, it polymerizes into *para*-glyoxal. A small quantity of water appears to accelerate the polymerization process, while a larger amount of water promotes extensive hydration, which, in turn, inhibits polymerization. Glyoxal, while a suspected carcinogen, is less toxic (LD_50_, rat > 2960 mg kg^−1^; LD_50_, mouse > 1280 mg kg^−1^). Higher LD_50_ values indicate lower toxicity. Glyoxal is typically not encountered in its pure form, as it is most commonly handled and stored as a 40% aqueous solution, allowing higher solubility of glyoxal than expected. The availability of water plays a key role in glyoxal chemistry. It forms a variety of hydrates, including oligomers known as dimers and trimers A–F (Fig. 1). Furthermore, hydrogen bonding within single glyoxal oligomers and between two oligomers and water molecules may provide stabilization of condensed-phase products.^1–4^ This review aims to provide a comprehensive account of glyoxal's use in heterocyclic synthesis. We cover its preparation, applications, and key reactions reported between 1953 and 2025, with a summary of the central reaction pathways presented schematically in Fig. 2.

## 2. Preparation of glyoxal

Glyoxal is non-volatile and potentially derivable from natural sources.^5^ It was initially synthesized through the reaction of ethanol with nitric acid and named by the German-British chemist Heinrich Debus.^6,7^ Additionally, numerous patents have been published on the dehydrogenation of ethylene glycol. The most effective method for producing glyoxal involves the dehydrogenation of ethylene glycol in the presence of oxygen using various catalysts, such as a copper catalyst,^8,9^ copper oxide supported on alumina,^10,11^ and a silver catalyst in the spiral form.^12^ Moreover, glyoxal is synthesized in the laboratory by oxidation of acetaldehyde with selenious acid^13^ or by ozonolysis of benzene.^14^ Furthermore, in 1979, Qingjiang reported additional methods for glyoxal production, such as acetylene oxidation, ethylene oxidation, oxalic acid reduction hydrolysis, and ethylene glycol gas-phase oxidation methods.^15^ In 1999, Minhas and co-workers demonstrated that glyoxal can be produced through the degradation of glucose *via* retroaldol condensation and autoxidation reactions.^16^ In 2011, Li and colleagues outlined the industrial synthesis of glyoxal using the acetaldehyde nitric acid oxidation method.^17^ Lange *et al.* highlighted that glyoxal is derived from both exogenous and endogenous sources. Endogenously, glyoxal is produced through the catabolism of carbohydrates, proteins, and fats.^18^ In summary, a range of ethylene glycol oxidation methods under varying conditions has been reported in the literature.^19–25^

## 3. Applications of glyoxal

Glyoxal is highly reactive, participating in addition, condensation, and crosslinking reactions with various compounds containing alcohols, amines, aldehydes, carboxyl groups, cellulose, polyvinyl alcohol, and urea. It finds extensive applications across diverse industries, including textiles, printing and dyeing, construction materials, leather, pharmaceuticals, and more, demonstrating significant potential for further development and utilization.^26^ Additionally, the development of resin adhesives by replacing formaldehyde with low-toxicity, environmentally friendly, and easily degradable glyoxal has emerged as a prominent and innovative focus in the industry.^27^ It has been widely used as a green environmental additive in the paper-making and textile industries.^28^ In wood adhesives, it is mainly used to substitute, partially or totally, the crosslinking agent or curing agent in natural wood adhesives such as tannin-based adhesives,^29,30^ lignin-based adhesives^31^ and protein-based adhesives.^32^ Glyoxal serves as a wrinkle-resistant finishing agent for cotton fabrics, creating cross-links with fibers through condensation reactions, thereby achieving wrinkle-resistant effects.^33^ Additionally, combining ethylene glycol with glyoxal can enhance the strength of the fabric.^34^ Also, glyoxal is safe for use in products intended to be applied to the nail at concentrations ≤1.25%.^35^

## 4. Glyoxal reactions

In 1953, Whisenhunt graduated from the University of Oklahoma. He wrote his thesis on the novel reactions of glyoxal,” which presented a series of glyoxal reactions for the synthesis of acyclic and heterocyclic organic compounds such as the reaction of glyoxal with isobutylene, 1,2-propanediol, 1,3-propanediol, 1,3-butanediol, 2-hydroxyethylamine, oxamide and lactic acid, preparation of acyclic and cyclic acetals of glyoxal and glyoxal tetraacetate.^36^

### 4.1. Synthesis of five-membered heterocycles

#### 4.1.1. Synthesis of imidazolidinones and imidazolidinthiones

**Scheme 1:**
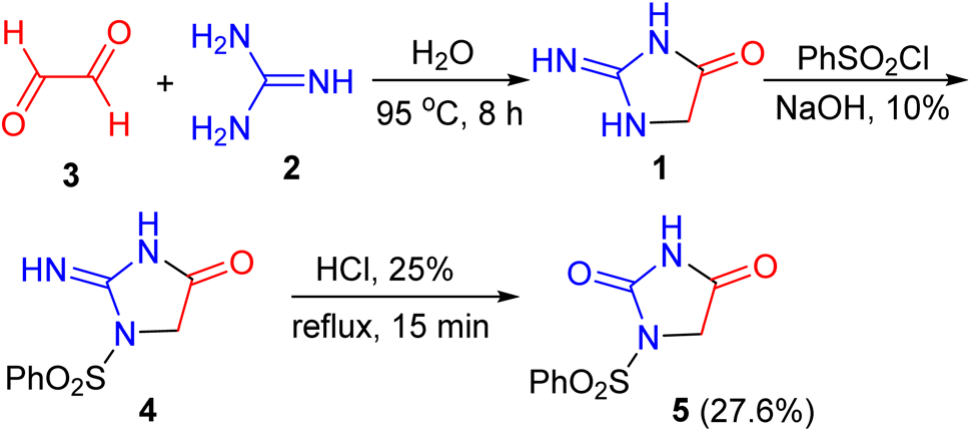
Synthesis of l-benzenesulfonyl-2,4-imidazoledione 5.

In 1953, Bengelsdo reported a new synthesis of 2-imino-4-imidazolidone (glycocyamidine) (1) by the interaction of guanidine hydrochloride (2) with aqueous glyoxal (3) in water at 95 °C for 8 h. Compound 1, by treatment with benzenesulfonyl chloride in the presence of NaOH solution, yielded l-benzenesulfonyl-2-imino-4-imidazolidone (4), which readily hydrolyzed with 25% hydrochloric acid solution to l-benzenesulfonyl-2,4-imidazoledione (5) (Scheme 1).^37^

**Scheme 2:**
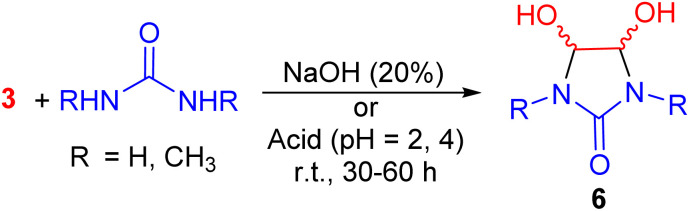
Synthesis of 4,5-dihydroxy-2-imidazolidinones 6.

In 1965, the Vail group investigated the reaction of glyoxal with *N*,*N*′-dimethylurea and urea under both acidic and basic conditions, leading to the formation of 4,5-dihydroxy-2-imidazolidinones 6. Rates for the formation of *cis*- and *trans*-4,5-dihydroxy-1,3-dimethyl-2-imidazolidinone and for the conversion of the pure isomers to an equilibrium mixture at various pH values were examined by NMR spectroscopy. It is probable that equimolar amounts of the *cis*- and *trans*-isomers are formed initially by a nonstereospecific addition, but the less stable *cis*-isomer is rapidly converted into the *trans* form under the conditions of the reaction. The resulting equilibrium mixture of products is predominantly the *trans*-isomer (Scheme 2).^38^

**Scheme 3:**
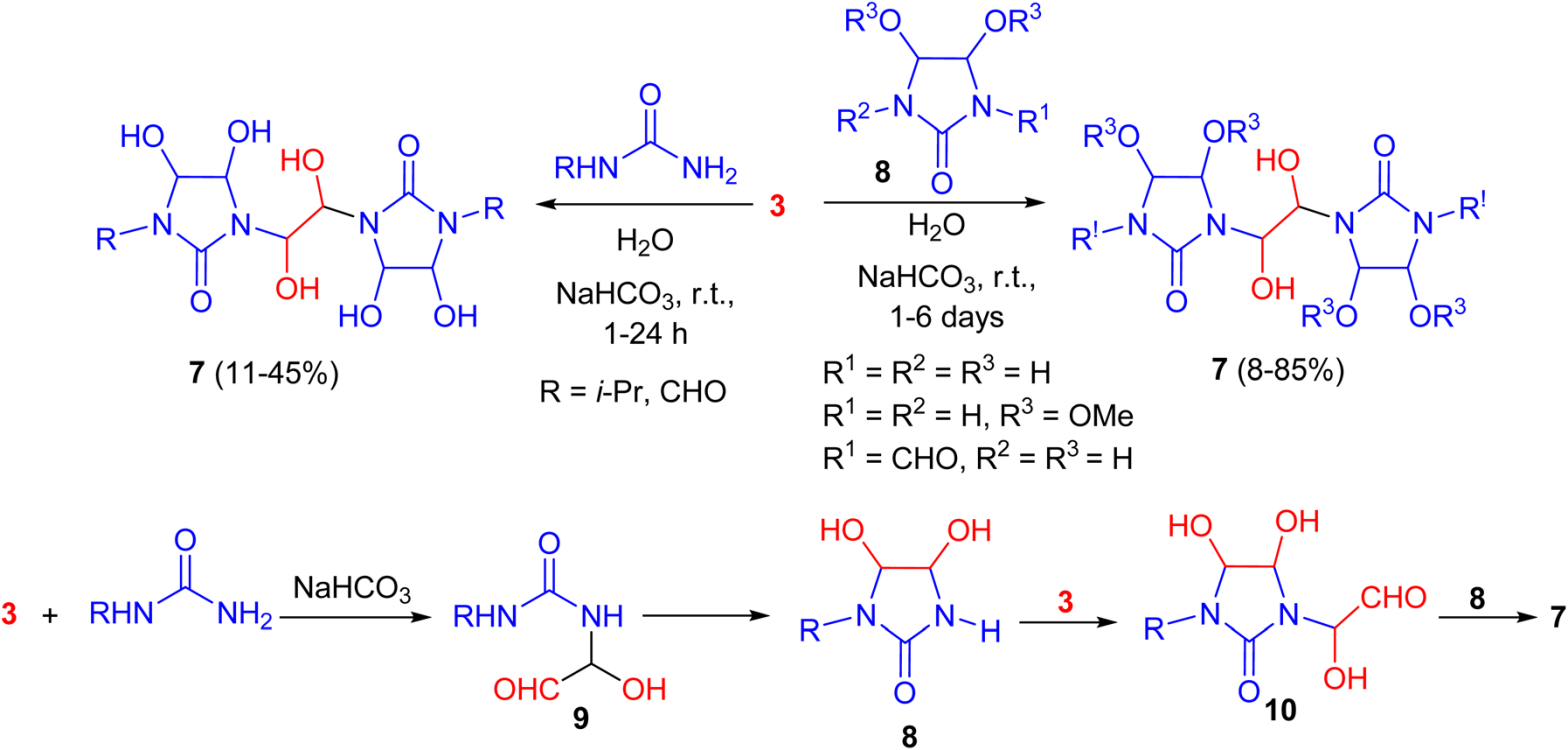
Preparation of 1.2-bis-(2-oxoirnidazolidin-*l*-yl)ethane-1.2-diols 7.

Next, 1,2-bis-(2-oxoirnidazolidin-*l*-yl)ethane-1,2-diol, and the corresponding derivatives 7, were synthesized in 8–85% yields by the reaction of glyoxal with imidazolidin-2-one and its derivatives 8 in water at room temperature for 1–6 days. These compounds were also directly prepared in 11–45% yields by the reaction of glyoxal with urea or mono-substituted urea in water at room temperature for 1–24 h. The mechanism proposed for the base-catalysed addition of glyoxal to urea involves abstraction of a proton from the urea and formation of an intermediate 9 by attack of the resultant nucleophile on glyoxal. The intermediate 9 then cyclises to an imidazolidin-2-one (8). In the case of mono-substituted urea, the resultant imidazolidine-2-one may then lose the remaining proton on the nitrogen atom and attack a further molecule of glyoxal to form the intermediate 10. Addition of this intermediate either to a second molecule of l-substituted imidazolidin-2-one, or to the monosubstituted urea, followed by addition of glyoxal, would then give the observed product 7 (Scheme 3).^39^

**Scheme 4:**
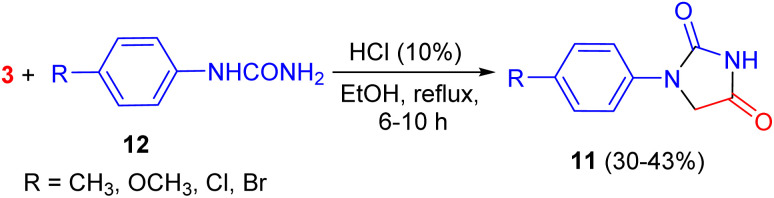
HCl-catalyzed synthesis of 1-aryl substituted hydantoins 11.

After that, Nematollahi and co-workers reported a series of 1-aryl substituted hydantoins 11 in 30–43% yields by condensation of monosubstituted ureas 12 and glyoxal in the presence of 10% solution of hydrochloric acid in ethanol under reflux for 6–10 h. An analysis of the experimental results was conducted to determine how specific electronic and steric factors contribute to both the synthetic utility and the mechanistic pathway of the urea-glyoxal condensation (Scheme 4).^40^

**Scheme 5:**
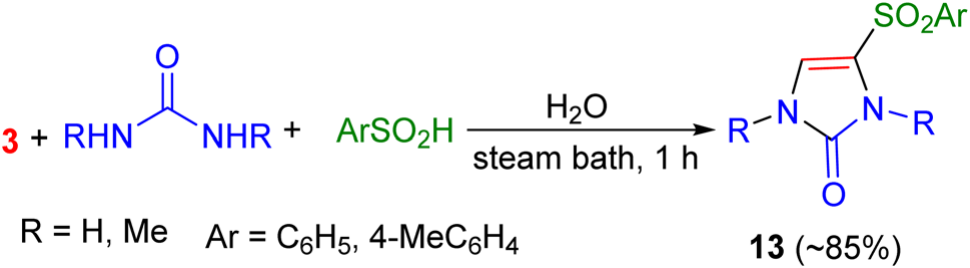
Green synthesis of arylsulfonyl-2-imidazolinones 13.

In 1996, Shutalev and Sivova described the reaction of glyoxal with urea or 1,3-dimethylurea and *p*-toluenesulfinic acid or benzenesulfinic acid in water on a steam bath for 1 h, which resulted in the formation of arylsulfonyl-2-imidazolidinones 13 in yields of about 85% (Scheme 5).^41^

**Scheme 6:**
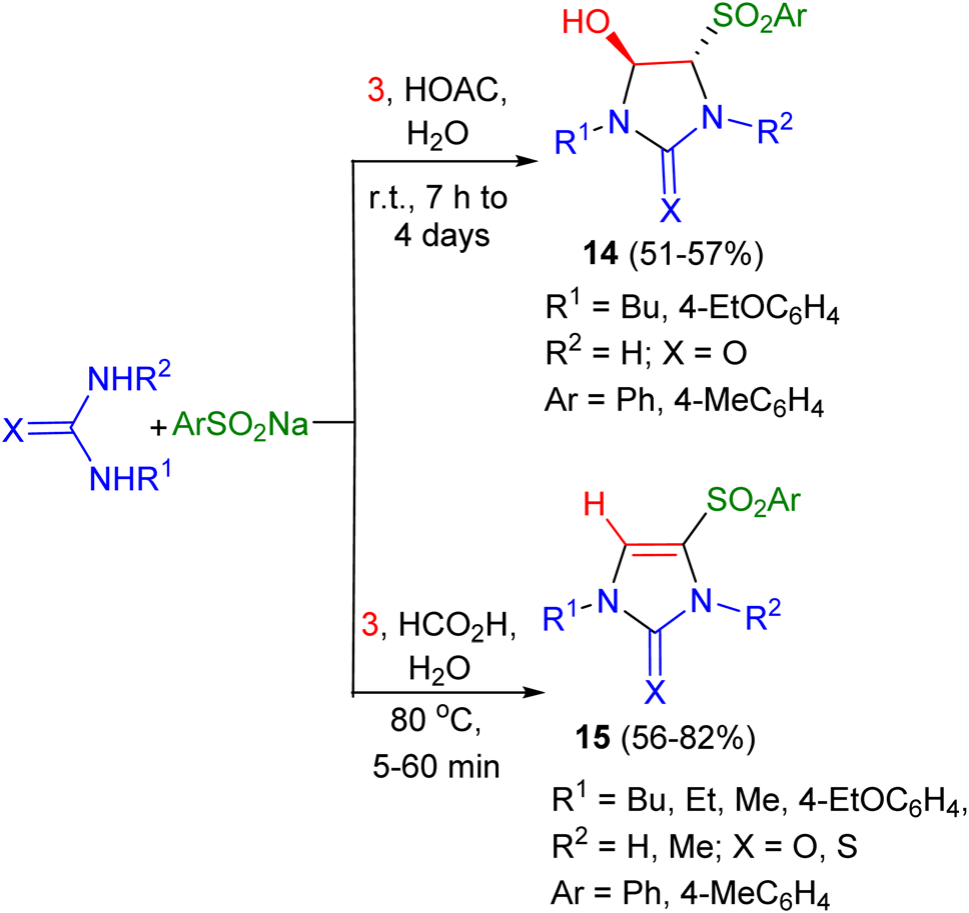
Synthesis of imidazolidin-2-ones 14 and 1,3-dihydroimidazol-2-ones/2-thione 15.

In 2000, Liepa and Wright developed a method for the synthesis of imidazolidin-2-one derivatives 14 in 51–57% yields by the reaction of glyoxal with hydrated sodium *p*-toluenesulfinate or sodium benzenesulfinate and urea derivatives in HOAc/H_2_O at room temperature for 7 h to 4 days. Additionally, 1,3-dihydroimidazol-2-ones/1,3-dihydroimidazolo-2-thione derivatives 15 were prepared in 56–82% yields through the reaction of glyoxal with sodium *p*-toluenesulfinate or sodium benzenesulfinate and urea derivatives using formic acid as a catalyst in H_2_O at 80 °C for 5–60 min (Scheme 6).^42^

**Scheme 7:**
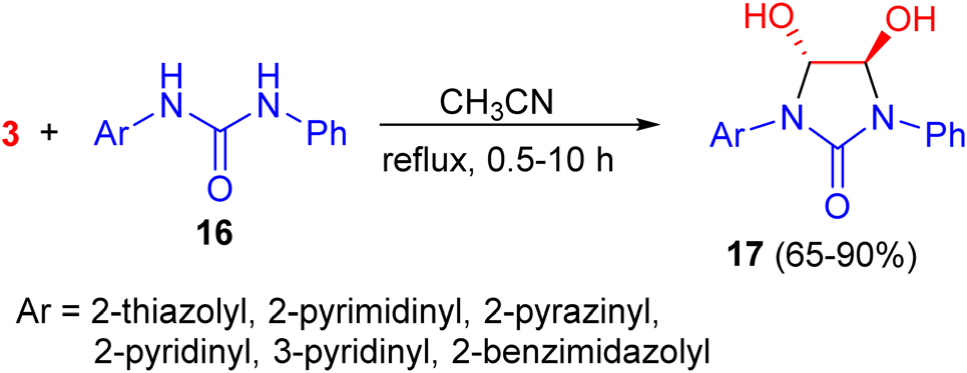
Synthesis of unsymmetrical 4,5-dihydroxy-2-imidazolidinones 17.

In 2006, Gandi and his group reported a formic acid-catalyzed cyclocondensation reaction of aqueous glyoxal with *N*-heteroaryl-*N*′-phenylureas (16) in acetonitrile under reflux conditions. This reaction yielded the corresponding 1-heteroaryl-3-phenyl-4,5-dihydroxy-2-imidazolidinones 17 in 65–90% yields within 0.5–10 hours. Physicochemical studies revealed that the NH proton in benzimidazoles undergoes rapid migration between the two nitrogen atoms, a phenomenon known as degenerate tautomerism. For prototropic tautomerism of 1-(2-benzimidazolyl)-3-phenyl-4,5-dihydroxy-2-imidazolidinone, the free-energy barrier (Δ*G*^≠^) was determined to be 81 ± 2 kJ mol^−1^ (Scheme 7).^43^

**Scheme 8:**
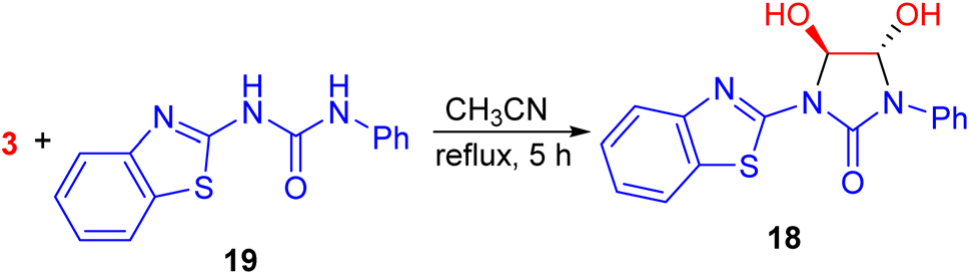
Synthesis of imidazolidin-2-one 18.

The synthesis of 1-(1,3-benzothiazol-2-yl)-4,5-dihydroxy-3-phenylimidazolidin-2-one (18) was achieved through a formic acid-catalyzed reaction between *N*-(benzothiazol-2-yl)-*N*′-phenylurea (19) and glyoxal in refluxing acetonitrile for 5 h. X-ray crystallographic analysis revealed key structural features: (1) the two hydroxyl groups adopt an anti-configuration relative to each other, (2) the benzothiazolyl and phenyl rings form a distinct dihedral angle, and (3) the crystal packing is stabilized by relatively strong intermolecular hydrogen bonds between adjacent molecules (Scheme 8).^44^

**Scheme 9:**
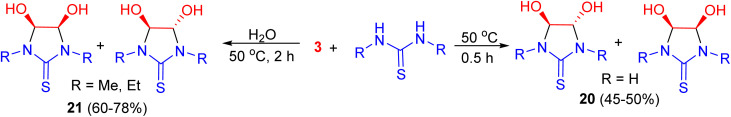
Synthesis of the 4,5-dihydroxyimidazolidine-2-thione derivatives 20 and 21.

In 2009, Nelyubina and colleagues synthesized 4,5-dihydroxyimidazolidine-2-thione derivatives 20 and 21 through two distinct routes: The reaction of thiourea with glyoxal at 50 °C for 30 minutes, yielding products 20 in 45–50%, and the reaction of thiourea derivatives with glyoxal trimer dihydrate in water using NaHCO_3_ at 50 °C for 2 h, yielding products 21 in 60–78%. In both cases, the products were obtained as mixtures of *cis*- and *trans*-isomers. A comparative study of the compounds and their carbonyl analogues, analyzing electron density distribution in their crystals, explained the differences in the number of and geometric parameters for hydrogen bonds involving their (thio)carbonyl groups (Scheme 9).^45^

**Scheme 10:**
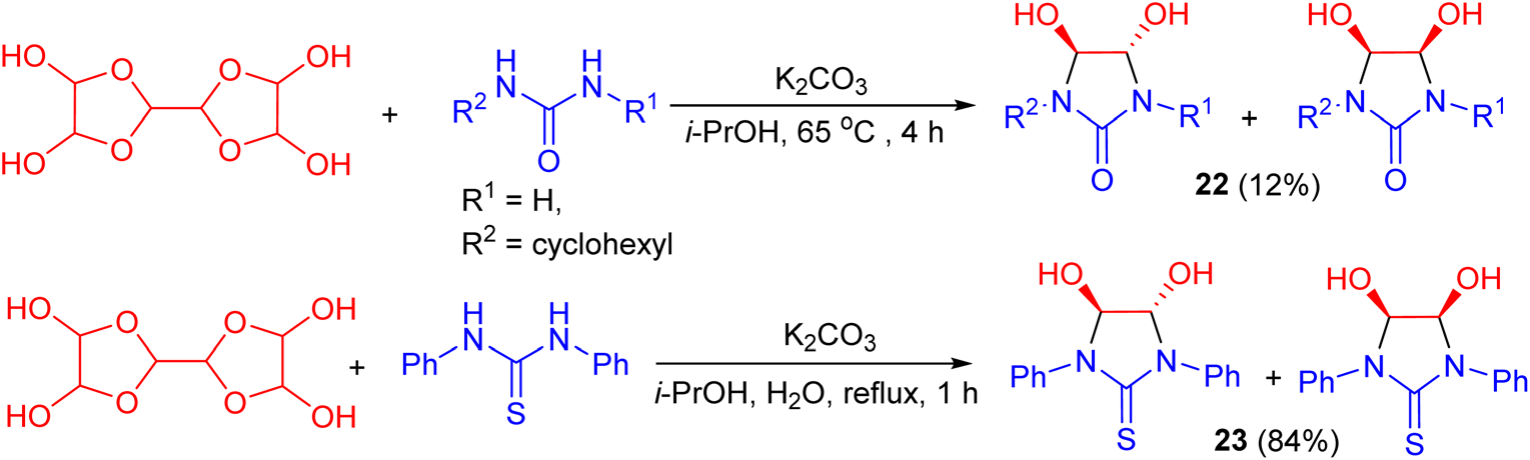
Preparation of 4,5-dihydroxyimidazolidin-2-ones/thiones 22 and 23.

Next, Kravchenko *et al.* described the diastereoselective synthesis of 4,5-dihydroxyimidazolidin-2-ones (thiones) 22 and 23 in yields of 12% and 84%, respectively. The reactions involved the condensation of glyoxal (as its trimeric dihydrate) with urea or thiourea under different conditions: For 22, the reaction was carried out with urea in the presence of K_2_CO_3_ in *i*-PrOH at 65 °C for 4 h, whereas for 23, thiourea was used in a mixed H_2_O/*i*-PrOH solvent system under reflux for 1 h. X-ray diffraction analysis confirmed the structures of the diastereomers. Additionally, ^1^H-NMR spectroscopy revealed a diastereomeric ratio of 50 : 1 for 22 and 11 : 1 for 23 (Scheme 10).^46^

**Scheme 11:**
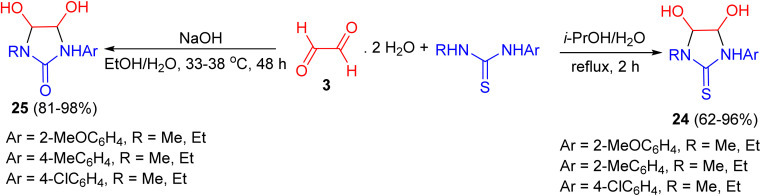
Synthesis of 4,5-dihydroxyimidazolidine-2-thiones (24) and 4,5-dihydroxyimidazolidin-2-ones (25).

In 2025, Izmestev *et al.* reported that 1,3-disubstituted thioureas and ureas react with glyoxal trimer dihydrate to afford 4,5-dihydroxyimidazolidine-2-thiones 24 and 4,5-dihydroxyimidazolidin-2-ones 25, respectively. The thiourea-derived heterocycles 24 were obtained in 62–96% yield after 2 h under reflux in *i*-PrOH/H_2_O, while the urea-derived analogues 25 were formed in higher yields (81–98%) in 48 h at 35 °C in EtOH/H_2_O (Scheme 11).^47^

#### 4.1.2. Synthesis of glycolurils

**Scheme 12:**
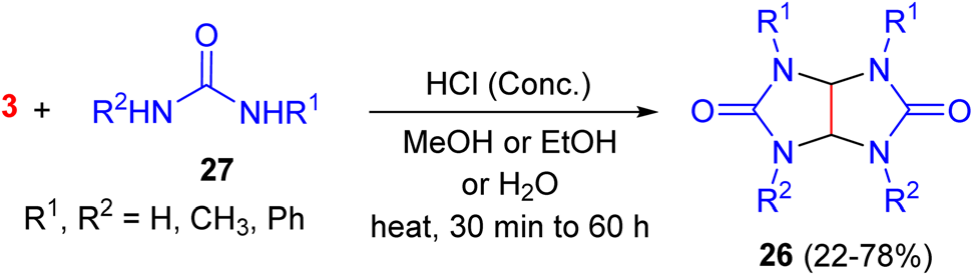
HCl-catalyzed synthesis of imidazoimidazoles 26.

In 1963, tetrahydroimidazo[4,5-*d*]imidazole-2,5-diones 26 were synthesized in 22–78% yields by the condensations of a variety of substituted ureas 27 with glyoxal in the presence of HCl as a catalyst in MeOH, EtOH or H_2_O under heating for 30 min to 60 h (Scheme 12).^48^ The dipole moment of the tetramethyl derivative was determined in benzene according to the method of Halverstadt and Kumler. The observed dipole moment was 4.05 D, thereby proving that these compounds have the expected *cis*-geometry.^49^

**Scheme 13:**
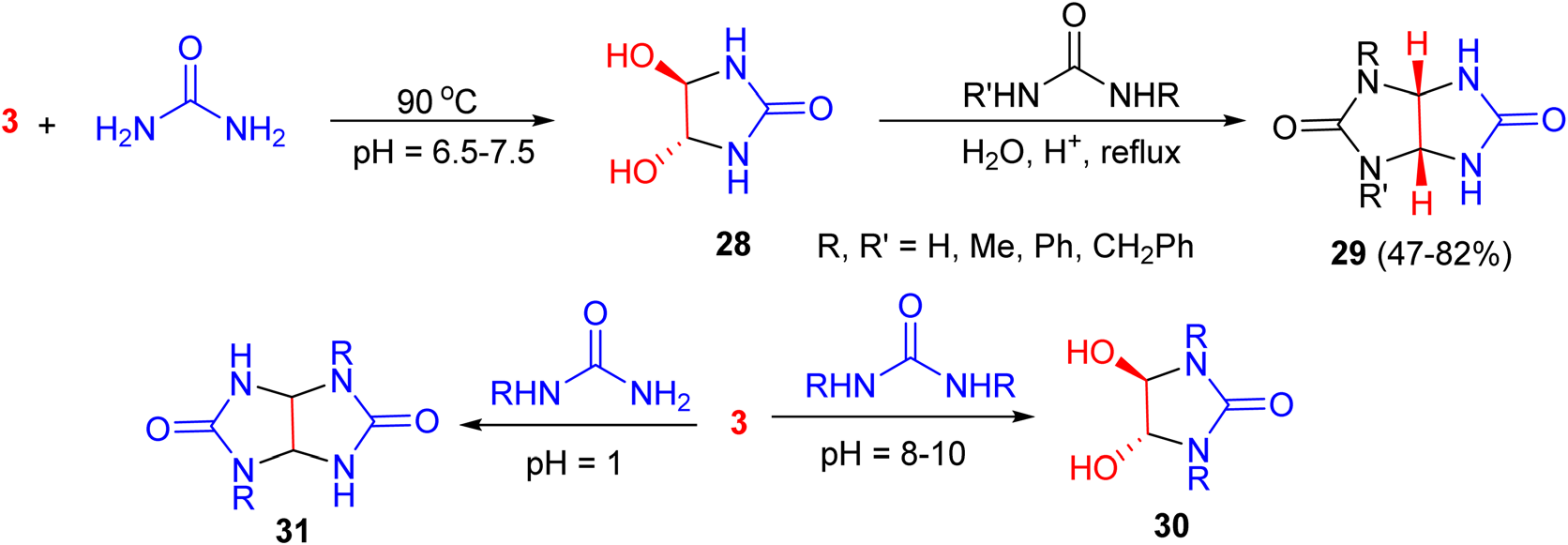
Synthesis of the imidazolidinones 28 and 30, and glycoluril derivatives 29 and 31.

In 1988, Grillona *et al.* presented the synthesis of *trans*-4,5-dihydroxy-2-imidazolidinone (28) by the reaction of urea with glyoxal at 90 °C and pH 6.5–7.5. When 28 reacted with urea, monoalkyl- or aryl-urea, or 1.3-dialkylurea under acidic conditions, glycoluril, monoalkyl- or aryl-glycolurils or l,3-dialkylglycolurils 29 were obtained in 47–82% yields, respectively. Also, the reaction of urea derivatives with glyoxal under base-catalyzed conditions (pH 8–10) and acidic conditions (pH 1) afforded imidazolidinones 30 and glycoluril derivatives 31, respectively (Scheme 13).^50^

**Scheme 14:**
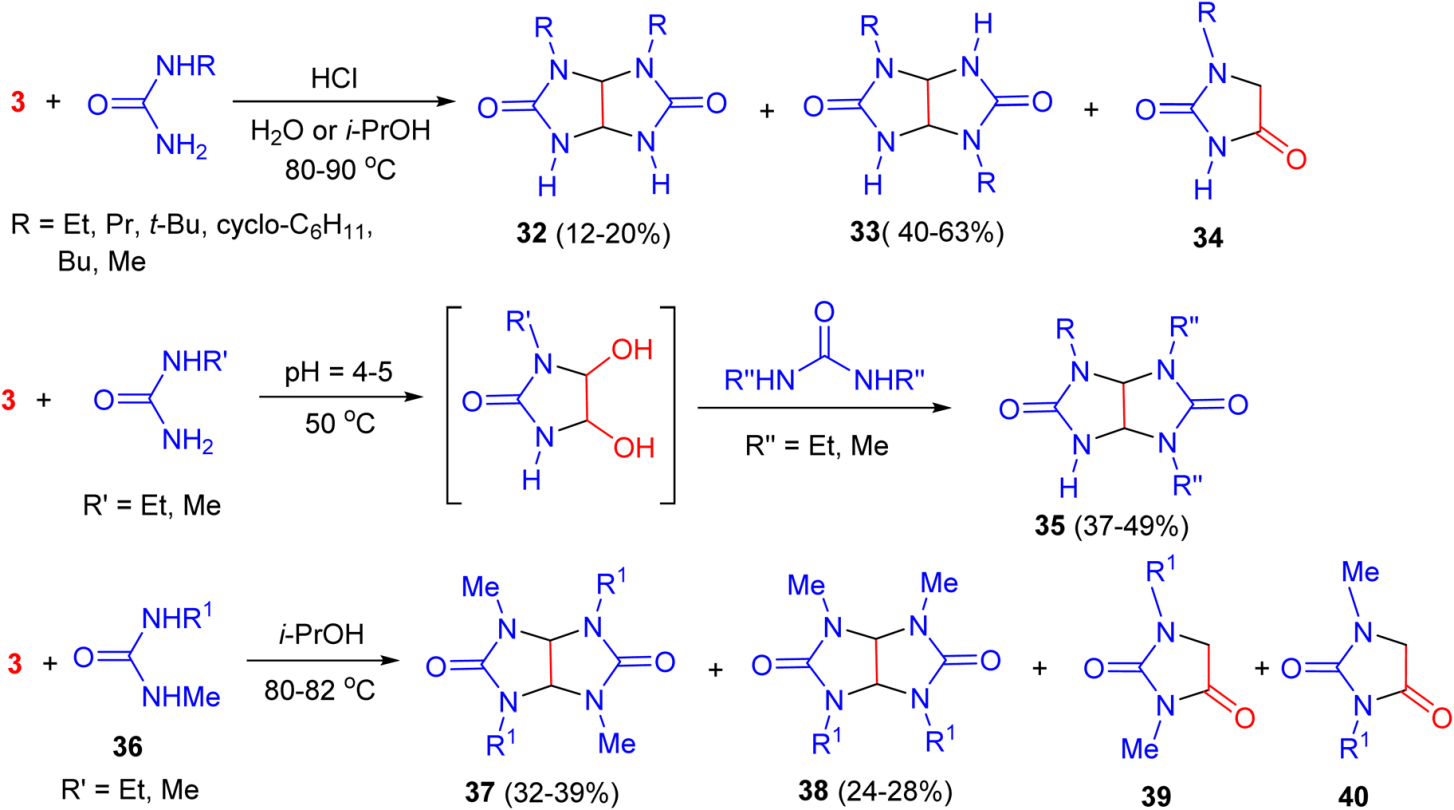
Synthesis of chiral mono-, di-, tri-, and tetraalkylglycolurils 32, 33, 35, 37 and 38.

In 2005, Kravchenko and co-workers synthesized chiral dialkylglycolurils 32 and 33 by the reaction of glyoxal with one or two moles of alkylureas in water or isopropanol at 80–90 °C in the presence of a catalytic amount of hydrochloric acid. The *trans*- to *cis*-isomer ratios (compounds 32 and 33) were estimated from the ratios of the signals for the protons of the CH–CH groups. The signals for these protons in the *trans*-isomers appear as singlets, whereas the corresponding signals for the *cis*-isomers appear as AMX systems. The yields of *trans*- and *cis*-glycolurils 32–33 were 40–63 and 12–20%, respectively. Hydantoins 34 were obtained as by-products. Moreover, tri-*N*-alkyl-glycolurils 35 were synthesized by the one-pot reaction of 1-alkyl-4,5-dihydroxyimidazolidin-2-ones (which were synthesized by the reactions of 1-alkylureas with glyoxal) with 1,3-dialkylureas in 37–49% yields. The reactions of dialkylureas 36 with glyoxal using acid catalyst occurred regioselectively to give *trans*-tetraalkylglycolurils 37 as the major products. The reactions also produced isomeric *cis*-tetraalkylglycolurils 38 and hydantoins 39 and 40. Hydantoins 39 and 40 were not isolated (Scheme 14).^51^

**Scheme 15:**
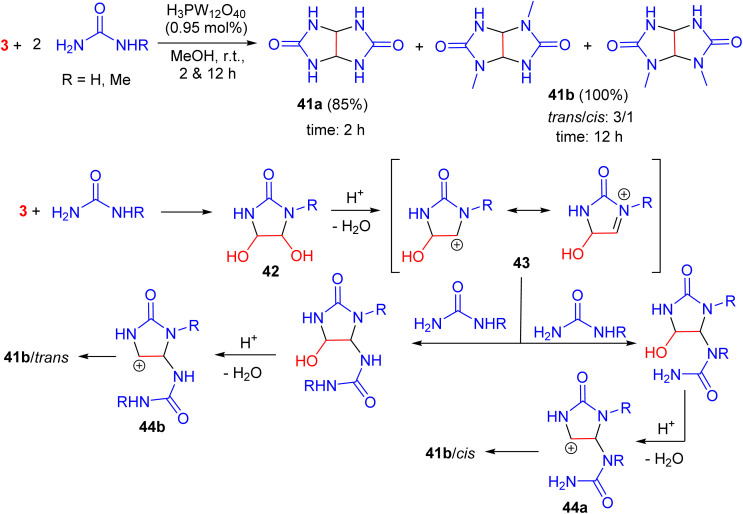
Keggin-type H_3_PW_12_O_40_ catalyzed synthesis of *cis*- and *trans*-alkyl substituted glycolurils 41a–b.

In 2012, Tayebee and his team achieved *cis*- and *trans*-alkyl substituted glycolurils 41a–b in high yields with a *cis*/*trans* ratio of 1 : 3. The reaction involved glyoxal and urea derivatives and was catalyzed by Keggin-type H_3_PW_12_O_40_ in methanol at room temperature, with a reaction time of 2–12 h. They proposed a reasonable reaction pathway as described in Scheme 15. In the first step, an intermediate, formulated as 1-alkyl-4,5-dihydroxyimidazolidin-2-one 42, would be formed from the reaction of glyoxal and mono-alkylurea molecules. Then, this intermediate was protonated in the presence of an acid catalyst, followed by elimination of a water molecule to generate the carbonium ion 43. The latter reacted with a second urea molecule to generate the final glycoluril through a new carbonium ion intermediate 44a–b. Two alternative reaction pathways through these two intermediates are possible, depending on which urea fragment, NH_2_ or NHR, is bound to the final carbonium ion to give the *cis*- and *trans*-isomers. However, the reactions of glyoxal with monoalkyl ureas always produced mixtures of *trans*- and *cis*-glycolurils.^52^

**Scheme 16:**
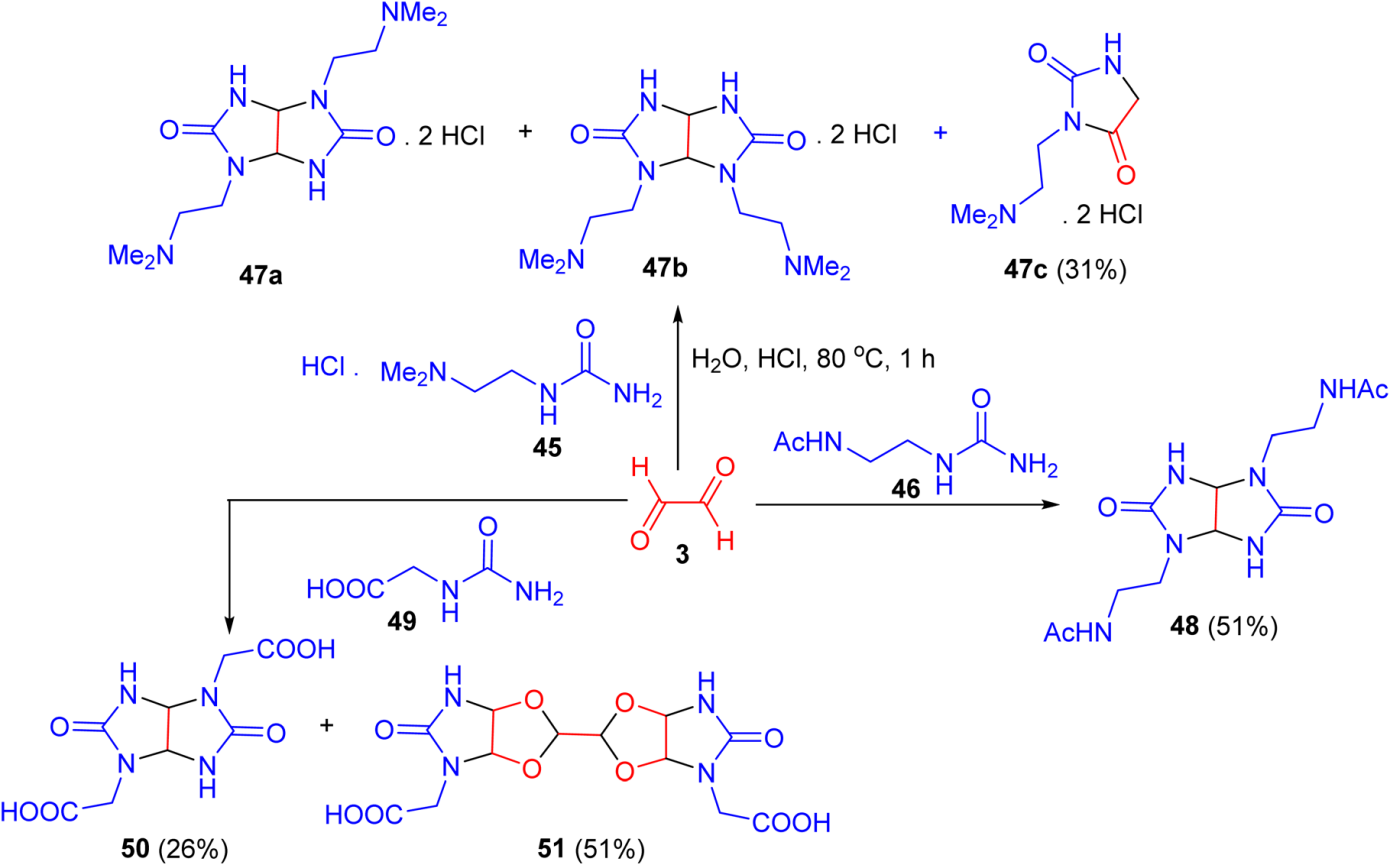
Regioselective synthesis of glycolurils 47, 48 and 50.

In 2014, a study demonstrated that treating glyoxal with either 1-[2-(dimethylamino)ethyl]urea (45) or 1-(2-acetylaminoethyl)urea (46) in water under acidic conditions (concentrated HCl, pH 1) at 80 °C for 1 h produced a mixture of *trans*- (47a) and *cis*-glycolurils (47b) in a 1 : 1 ratio (49% combined yield), or *trans*-isomer 48 (51% yield). However, the separation of 47a and 47b was unsuccessful. The ^1^H-NMR spectrum also revealed signals corresponding to the known hydantoin derivative 47c. Furthermore, when glyoxal was reacted with *N*-carbamoyl glycine (49) under similar conditions, the *trans*-isomer 50 was obtained in 26% yield, while the dimeric product 3,3′-bis[(1*R*,5*S*)-2,4-dioxa-6,8-diaza-7-oxobicyclo[3.3.0]oct-6-yl)acetic acid] (51) was formed in 51% yield (Scheme 16).^53^

**Scheme 17:**
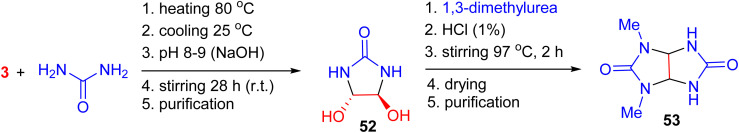
Synthesis of *trans*-4,5-dihydroxy-2-imidazolidinone 52 and 2,4-dimethylglycoluril 53.

Demets and his colleagues reported the synthesis of *trans*-4,5-dihydroxy-2-imidazolidinone (52) *via* the reaction of glyoxal with urea in the presence of NaOH at room temperature over 28 h. Subsequently, treating 52 with 1,3-dimethylurea in water under concentrated HCl at 97 °C for 2 h yielded 2,4-dimethylglycoluril (53). However, the instability of 53, which slowly decomposes into urea and 1,3-dimethylimidazolidine-2,4-dione, significantly reduced the synthesis yield (Scheme 17).^54^

**Scheme 18:**
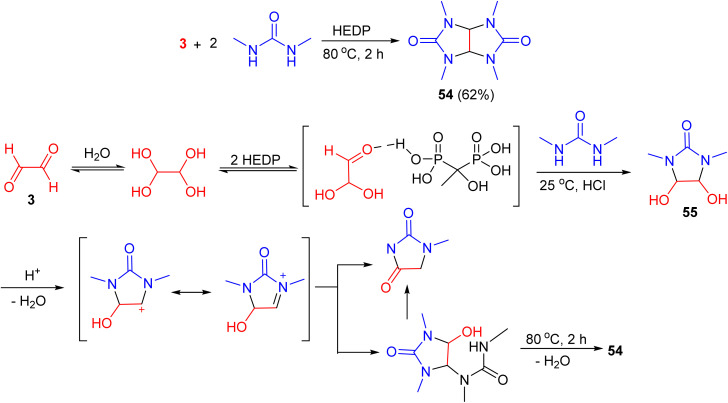
HEDP-catalyzed synthesis of tetramethylglycoluril 54.

Etidronic acid [1-hydroxyethane-1,1-diylbis(phosphonic acid), HEDP] as a green catalyst, was used for the synthesis of 2,4,6,8-tetramethyl-2,4,6,8-tetraazabicyclo[3.3.0]octane-3,7-dione 54 in 62% yield *via* the reaction of *N*,*N*′-dimethylurea and aqueous glyoxal at 80 °C for 2 h. A proposed mechanism is outlined in Scheme 18. According to the data gathered by NMR monitoring, 4,5-dihydroxy-1,3-dimethylimidazolidin-2-one 55 is formed as an intermediate product. The stepwise reaction mechanism was partially confirmed by NMR monitoring of model reactions. These studies suggest that the formation of target product 54 at elevated temperatures proceeds *via* several possible pathways, ultimately yielding tetramethylglycoluril **54**.^55^

#### 4.1.3. Synthesis of imidazolidines

**Scheme 19:**
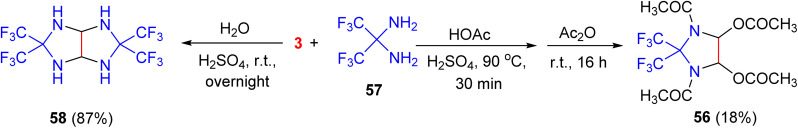
Synthesis of the imidazolidine derivative 56 and tetraaza-bicyclooctane derivative 58.

In 1987, Adolph and his group reported the synthesis of 2,2-bis(trifluoromethyl)-4,5-diacetoxy-l,3-diacetylimidazolidine (56) in 18% yield by condensation of 2,2-diaminohexafluoropropane (57) with glyoxal and acetic anhydride in HOAC containing H_2_SO_4_ as a catalyst. Additionally, 3,3,7,7-tetrakis(trifluoromethyl)-2,4,6,8-tetraazabicyclo-[3.3.0]octane (58) was synthesized in 87% yield through the reaction of 57 with glyoxal in H_2_O in the presence of H_2_SO_4_ at room temperature, overnight (Scheme 19).^56^

**Scheme 20:**
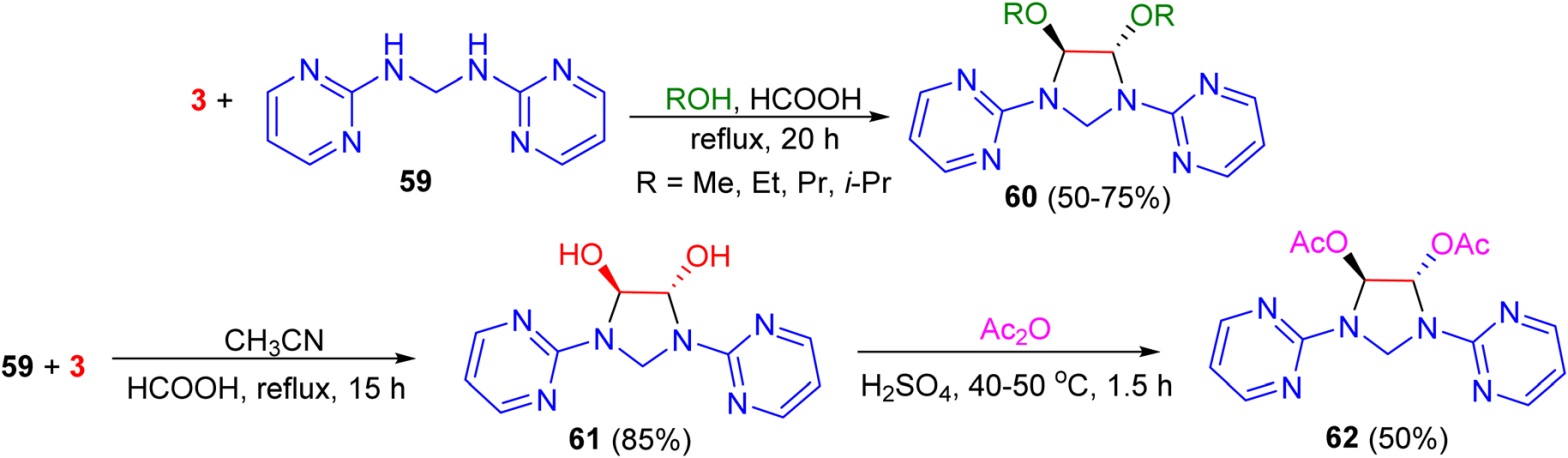
Synthesis of the imidazolidine derivatives 60–62.

In 2006, Ghandi and co-workers demonstrated that cyclocondensation of *N*,*N*′-bis(2-pyrimidinyl)methanediamine 59 with glyoxal in alcohols (MeOH, EtOH, PrOH and *i*-PrOH) using formic acid as a catalyst under reflux conditions for 20 h, led to the formation of the corresponding 4,5-dialkoxy-1,3-bis(2-pyrimidinyl)imidazolidines 60 in 50–75% yields. 4,5-Dihydroxy-1,3-bis(2-pyrimidinyl)imidazolidine 61 was obtained after 15 h in 85% yield when the reaction was carried out in refluxing acetonitrile in the presence of formic acid. Moreover, the reaction of compound 61 with acetic anhydride in the presence of H_2_SO_4_ at 40–50 °C for 1.5 h resulted in the formation of the corresponding *trans*-4,5-diacetoxy-1,3-bis(2-pyrimidinyl)imidazolidine 62 in 50% yield. Based on ^1^H-NMR analysis, it was found that the *trans*-isomers were selectively obtained in these cyclocondensation reactions (Scheme 20).^57^

**Scheme 21:**
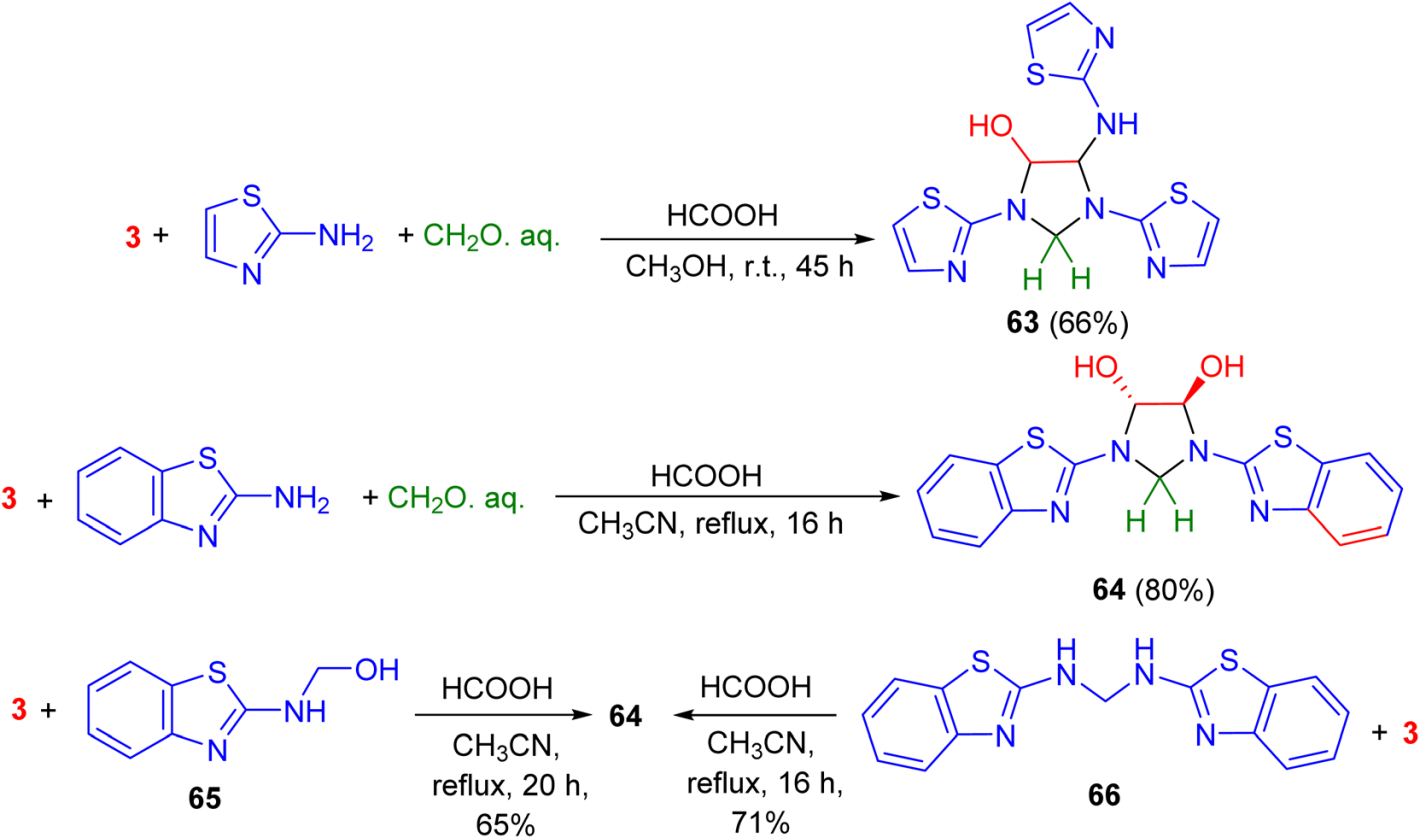
Preparation of imidazolidines 63 and 64.

In 2007, Ghandi and Olyaei revealed that the reaction of 2-aminothiazole with aqueous glyoxal and aqueous formaldehyde in MeOH using HCOOH as a catalyst at room temperature for 45 h produced imidazolidine 63 in 66% yield. On the other hand, acid-catalyzed one-pot three-component reaction of 2-aminobenzothiazole, aqueous glyoxal and aqueous formaldehyde in CH_3_CN under reflux conditions for 16 h afforded *trans*-4,5-dihydroxy-1,3-bis(2-benzothiazolyl)imidazolidine 64 in 80% yield. Finally, the reaction of compound 65 or 66 with aqueous glyoxal in refluxing CH_3_CN using HCOOH as a catalyst for 16–20 h produced 64 (Scheme 21).^58^

**Scheme 22:**
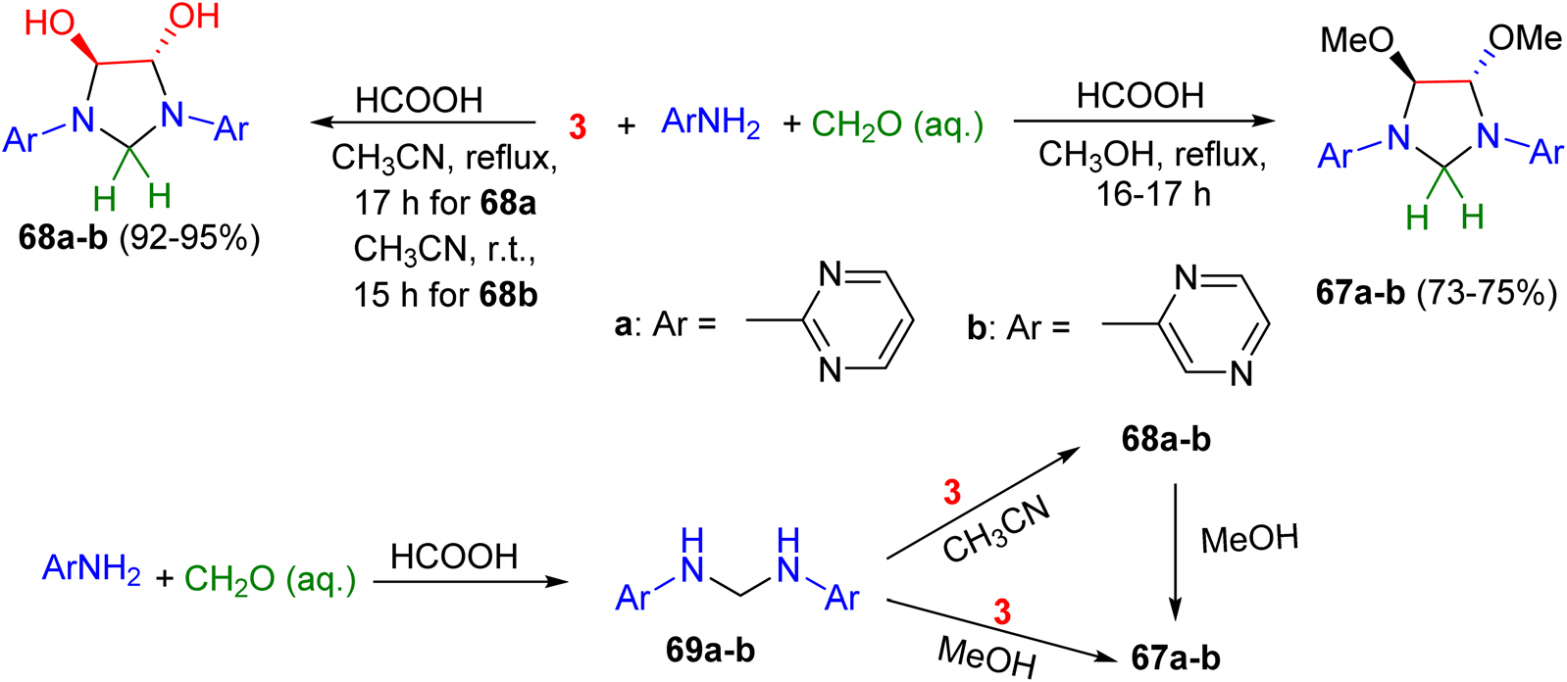
Synthesis of the imidazolidine derivatives 67 and 68.

Next, the four-component reaction of aminodiazines (2-aminopyrimidine and 2-aminopyrazine), glyoxal and formaldehyde in methanol under reflux conditions for 16–17 h afforded *trans*-4,5-dimetoxy-1,3-bis(2-pyrimidinyl)imidazolidine (67a) in 75% yield and *trans*-4,5-dimetoxy-1,3-bis(2-pyrazinyl)imidazolidine (67b) in 73% yield, respectively. Changing methanol to acetonitrile resulted in the formation of the corresponding 1,3-bis(2-pyrimidinyl) and-1,3-bis(2-pyrazinyl)-derivatives of *trans*-4,5-dihydroxyimidazolidine (68a–b) in 92–95% yields. The proposed mechanism is illustrated in Scheme 22. The condensation of aminodiazines with formaldehyde produces the intermediates 69a–b, which then undergo reaction with glyoxal to form 68a–b. Subsequently, the reaction of 68a–b with methanol leads to the formation of 67a–b, respectively.^59^

**Scheme 23:**
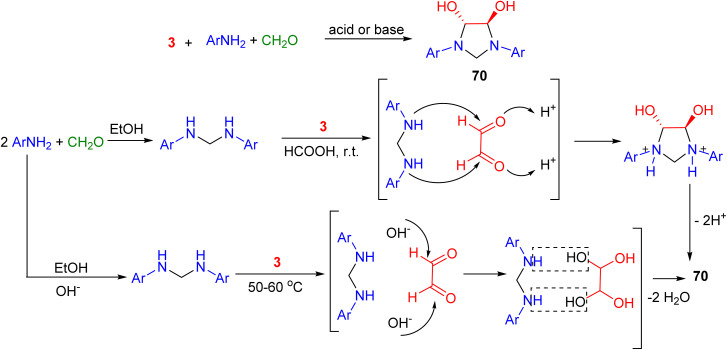
Synthesis of (±)-*trans*-4,5-dihydroxy-1,3-diarylimidazolidines 70.

Tian and co-workers synthesized (±)-*trans*-4,5-dihydroxy-1,3-diarylimidazolidines 70 by the condensation reaction of aromatic amines with glyoxal and formaldehyde, catalyzed by acid or base under a one- or two-step reaction sequence. The result shows that an aromatic amine with strong electron-withdrawing groups cannot produce the desired product, an aromatic amine with mild electron-withdrawing groups can produce the desired product by base catalysis at 50–60 °C, and an aromatic amine with a mild electron-withdrawing or electron-donating group may generate the desired product by acid catalysis with a two-step reaction procedure. A plausible reaction is illustrated in Scheme 23.^60^

**Scheme 24:**
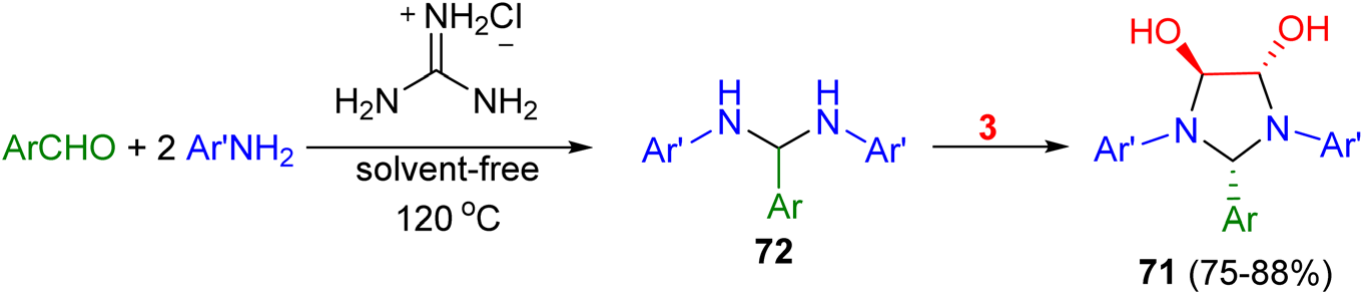
Synthesis of *trans*-4,5-dihydroxy-2-aryl-1,3-bis(heteroaryl)imidazolidines 71.

In addition, Olyaei and his group reported a facile, one-pot stereoselective synthesis of *trans*-4,5-dihydroxy-2-aryl-1,3-bis(heteroaryl)imidazolidines 71 in 75–88% yields by a cyclocondensation reaction of heteroarylamines, benzaldehydes and aqueous glyoxal in the presence of guanidinium chloride as a polyfunctional organocatalyst under solvent-free conditions for 23–76 minutes. The proposed mechanism is shown in Scheme 24. The catalyst initially acts as a hydrogen-bond donor to activate the aldehyde by the formation of a six-membered ring. Subsequently, a Schiff base was formed by nucleophilic addition of the amine to the aldehyde and dehydration in the presence of the catalyst acting as an acid. Next, the Schiff base is further attacked by a second amine to give the *gem*-diamine intermediate 72. Finally, nucleophilic addition of 72 to the carbonyl of the glyoxal gave the final product 71.^61^

**Scheme 25:**
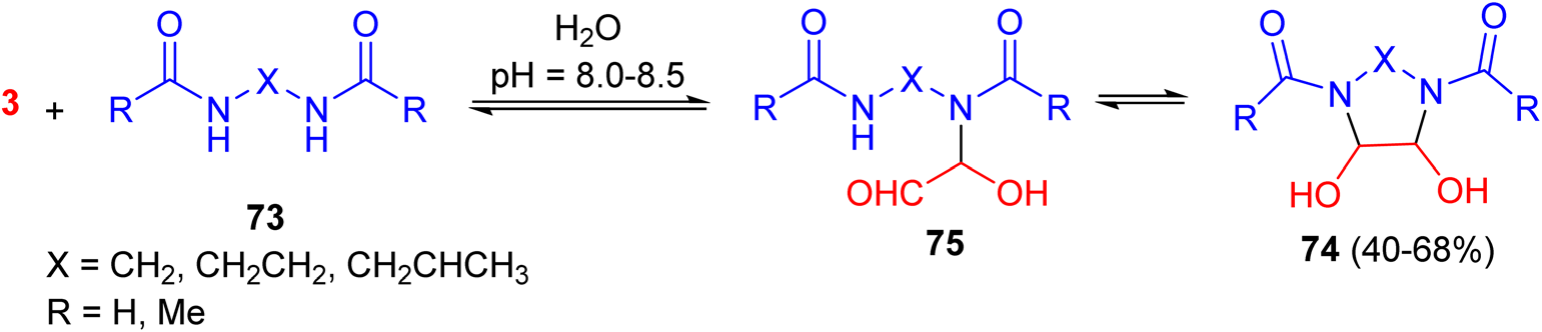
Synthesis of the bis-amide glyoxal products 74.

The Vail group disclosed that various bisamides of the type RCONH-X-NHCOR 73, when treated with glyoxal in water at room temperature for a few minutes to one day, produced, in some cases, the desired cyclic compounds 74 in 40–68% yields. The reaction is considered to be similar to urea glyoxal additions: addition to the carbonyl carbon of the glyoxal afforded intermediate 75, followed by cyclization to the product 74 (Scheme 25).^62^

#### 4.1.4. Synthesis of imidazo[4,5-*d*]imidazoles

**Scheme 26:**
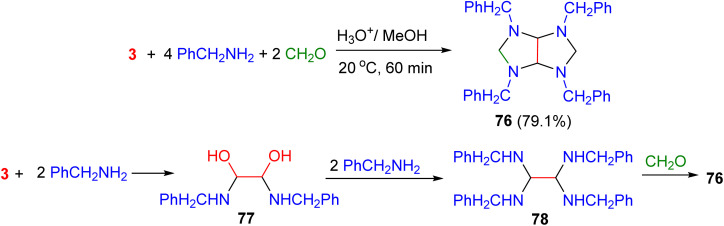
Synthesis of 2,4,6,8-tetrabenzyl-2,4,6,8-tetraazabicyclo[3.3.0]octan (76).

In 1992, Farnia *et al.* discovered that condensation of glyoxal with benzylamine and formaldehyde in acidic media gave 2,4,6,8-tetrabenzyl-2,4,6,8-tetraazabicyclo[3.3.0]octan (76) in 79.1% yield. The formation of 76 is believed to first go through diol 77, which can be isolated at 0 °C. When formaldehyde is present, diol 77 further reacts with additional amines to give tetraamine 78, which is then rapidly cyclized to 76. Based on the NMR spectroscopy results, the *cis*-structure with a *syn*-envelope conformation is proposed for 76 (Scheme 26).^63^

**Scheme 27:**
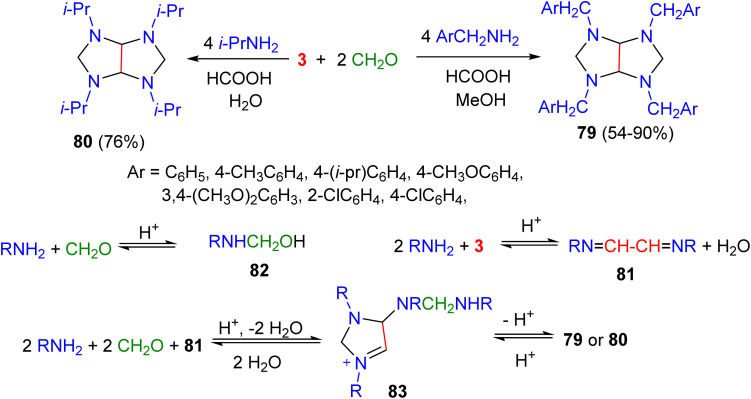
Formation of 2,4,6,8-tetrasubstituted-2,4,6,8-tetraazabicyclo[3.3.0]octanes 79 and 80.

Nielsen and his group reported that condensation of formaldehyde, glyoxal, and primary amines, in a stoichiometric ratio, led to the formation of 2,4,6,8-tetrasubstituted-2,4,6,8-tetraazabicyclo[3.3.0]octanes 79 in 54–90% yields in methanol solvent with formic acid catalyst. Additionally, 2,4,6,8-tetraisopropyl-2,4,6,8-tetraazabicyclo[3.3.0]octane 80 was synthesized in 76% yield by the reaction of isopropylamine with formaldehyde and glyoxal using formic acid catalyst in water. Also, these compounds decompose in acidic media. Thus, the relative stability of the tetrasubstituted bicyclooctanes in acidic media varies with substituents in the order: CH_3_ > C_6_H_5_CH_2_ > *i*-C_3_H_7_. The suggested mechanism is outlined in Scheme 27. The glyoxal diimine 81 is an important intermediate in the synthesis. The diimine can react stepwise with 2 mol of amine and formaldehyde, or with the methylolamine 82, to produce the monocyclic imidazoline cation 83, which, upon proton loss, yields the desired product.^64^

**Scheme 28:**
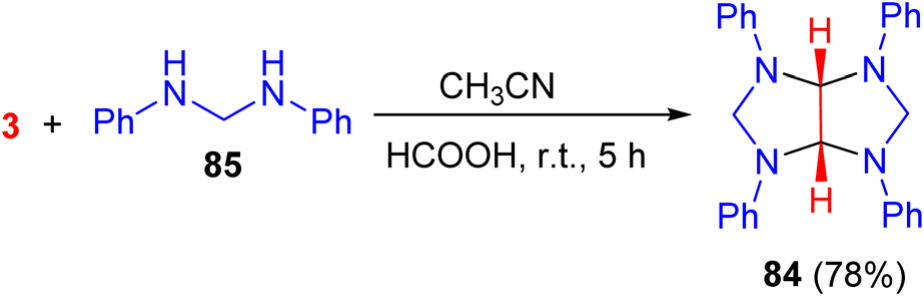
Synthesis of 2,4,6,8-tetraphenyl-2,4,6,8-tetraazabicyclo[3.3.0]octane 84.

After that, Ghandi and his group demonstrated the synthesis of 2,4,6,8-tetraphenyl-2,4,6,8-tetraazabicyclo[3.3.0]octane 84 in 78% yield by the reaction of *N*,*N*′-bisphenylmethanediamine 85 (2.0 mmol) with glyoxal (1.0 mmol, 40% aq.) in the presence of formic acid as a catalyst in acetonitrile at room temperature for 5 h (Scheme 28).^65^

#### 4.1.5. Synthesis of imidazoles and imidazolium zwitterions

**Scheme 29:**
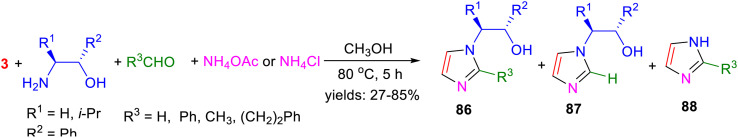
Synthesis of imidazoles 86–88.

In 2006, a highly versatile method for the preparation of enantiopure 1-substituted, 1,2-disubstituted, and 1,4,5-trisubstituted imidazoles 86–88 was developed by using the cyclocondensation reaction of glyoxal, an aldehyde, a 1,2-amino alcohol, and ammonium acetate at 80 °C for 5 h. The choice of an ammonium source, which has a significant influence on the pH of the reaction mixture, was found to be an important factor to determine the efficiency and selectivity of the reaction (Scheme 29).^66^

**Scheme 30:**
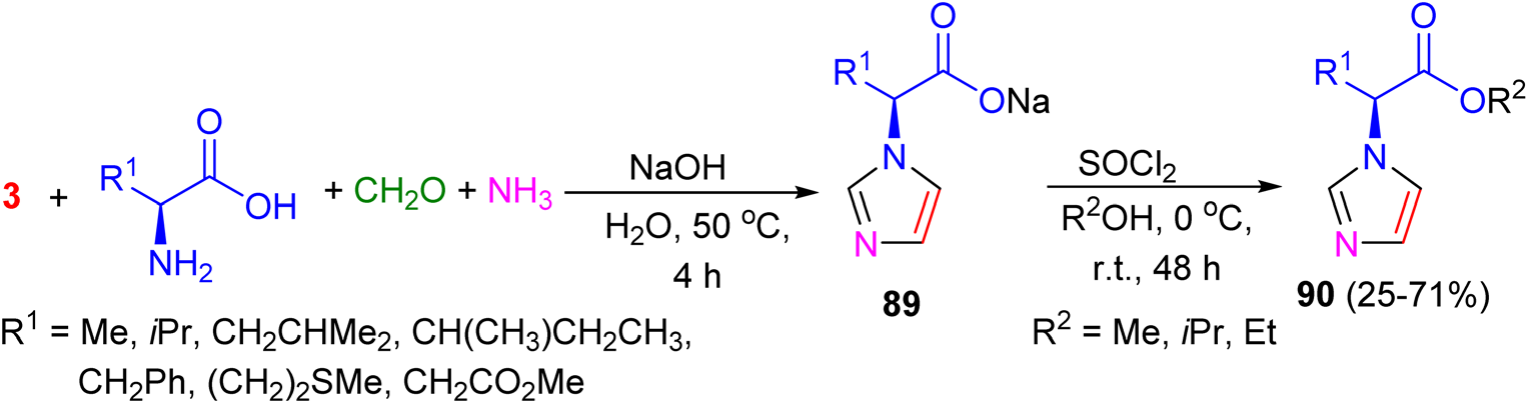
Synthesis of imidazoles 89 and 90.

Under basic conditions, amino acids reacted with glyoxal, formaldehyde, and ammonia in water at 50 °C for 4 h to afford chiral imidazoles 89. This intermediate was then treated with SOCl_2_ in different alcohols at room temperature for 48 h, leading to imidazoles 90 in 25–71% yields (Scheme 30).^67^

**Scheme 31:**
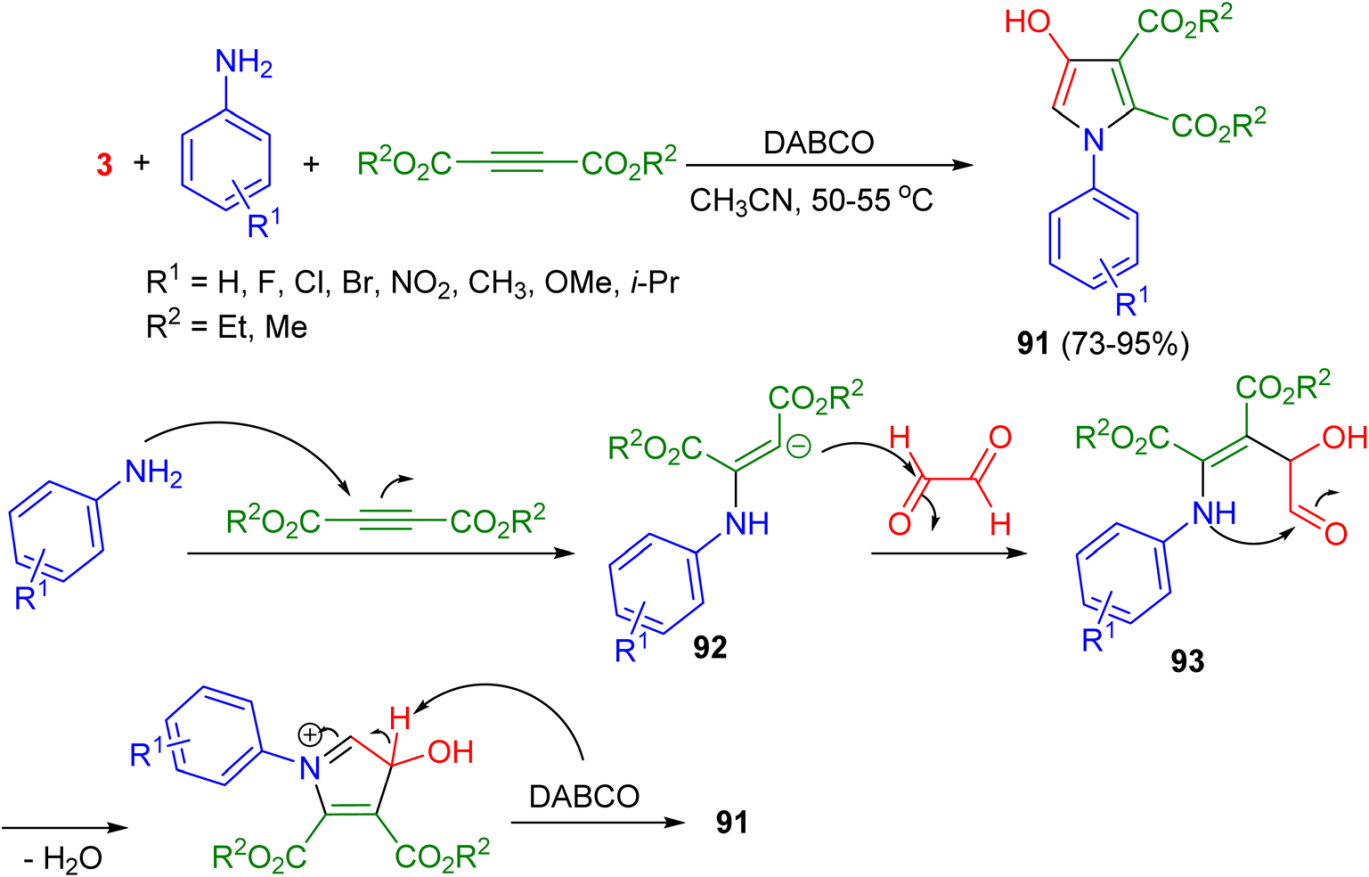
DABCO-catalyzed synthesis of highly substituted pyrroles 91.

In 2011, a simple and efficient three-component protocol for the synthesis of highly substituted pyrroles 91 in 73–95% yields was developed by using amines, DEAD/DMAD, and glyoxal in the presence of DABCO as a catalyst in CH_3_CN at 50–55 °C. It was observed that aromatic amines bearing electron-donating groups resulted in higher yields of the products compared to amines with electron-withdrawing groups. The proposed mechanism proceeds *via* the nucleophilic addition of an amine to DEAD, which generates intermediate 92; this enamine reacts with glyoxal to give intermediate 93, which undergoes condensation followed by proton abstraction by DABCO to yield the desired product 91 (Scheme 31).^68^

**Scheme 32:**
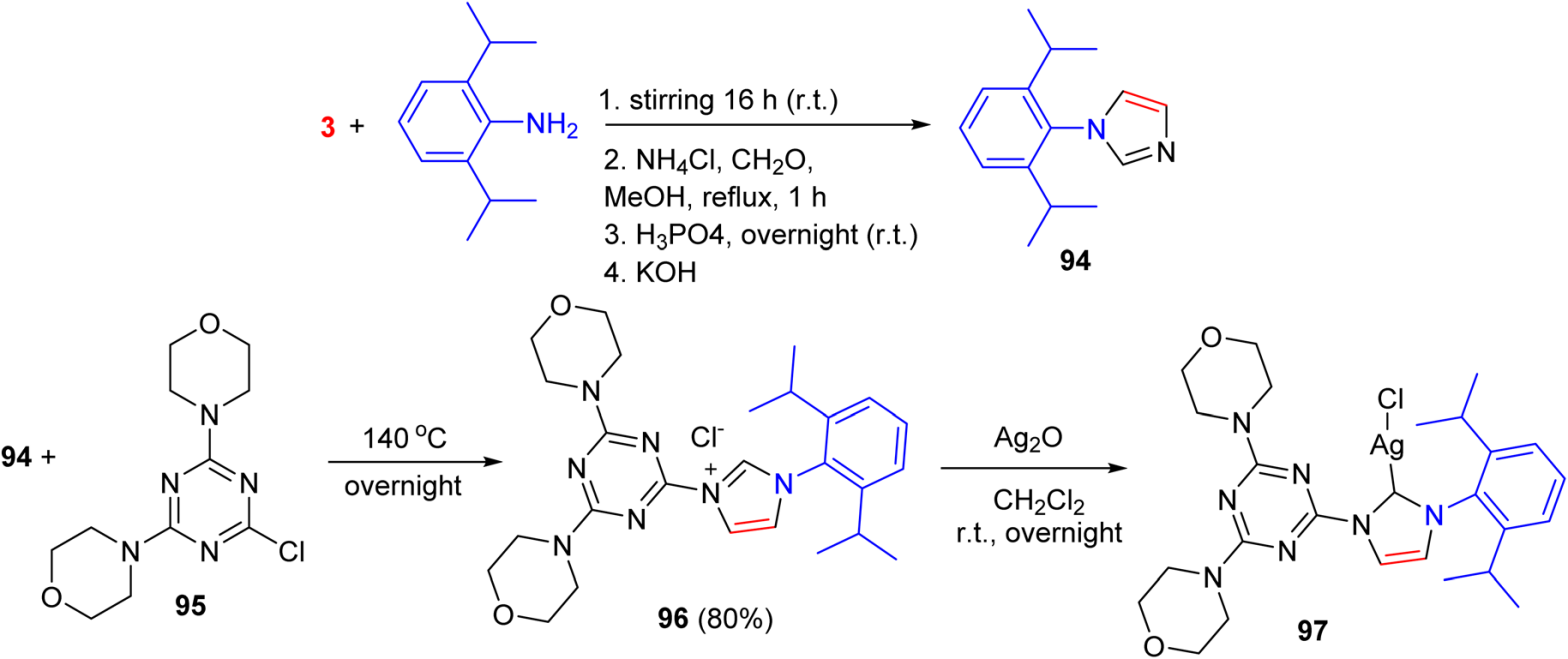
Synthesis of the Ag(i) NHC complex 97.

In 2017, Hasson reported the synthesis of 1-(2,6-diisopropylphenyl)-1*H*-imidazole (94) *via* the reaction of 2,6-diisopropylaniline with glyoxal and aqueous formaldehyde in the presence of NH_4_Cl and H_3_PO_4_ in MeOH. Subsequent treatment of 94 with 2,6-dimorpholino-4-chloro-1,3,5-triazine (95) at 140 °C overnight gave the imidazolium chloride (96) in 80% yield. Finally, reacting 96 with Ag_2_O in CH_2_Cl_2_ at room temperature overnight produced the Ag(i) NHC complex 97 (Scheme 32).^69^

**Scheme 33:**
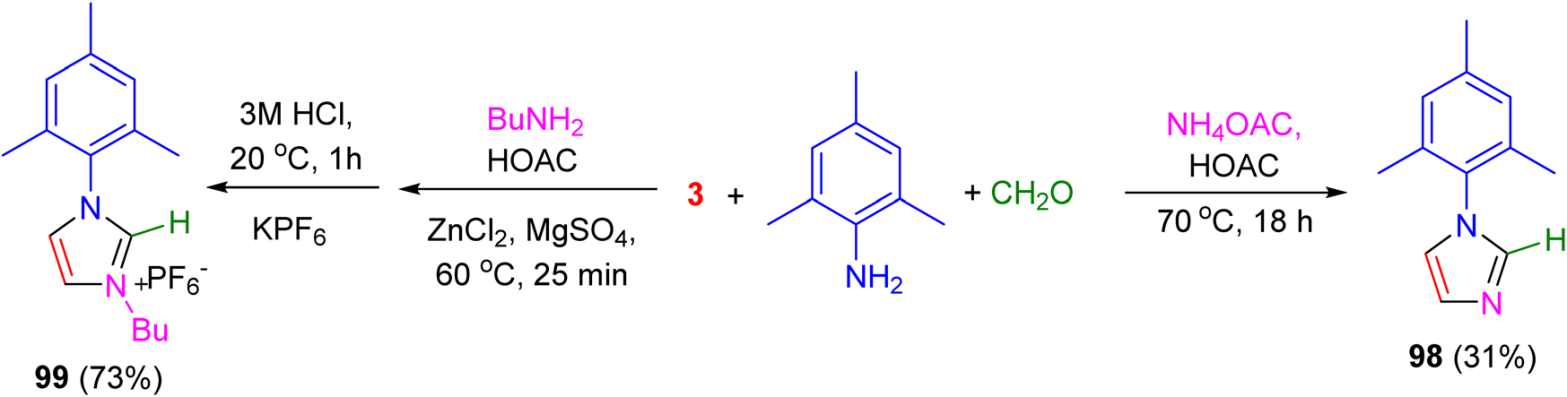
Synthesis of *N*-aryl- and *N*-alkyl-substituted imidazolium 98 and 99.

Bjørsvik and co-workers synthesized 1-(2,4,6-trimethylphenyl)-1*H*-imidazole (98) in 31% yield by condensing mesitylamine with glyoxal, aqueous formaldehyde, and ammonium acetate in acetic acid at 70 °C for 18 h. In a modified procedure, replacing ammonium acetate with butylamine in the presence of ZnCl_2_/MgSO_4_ at 60 °C for 25 minutes, followed by HCl addition and stirring at 20 °C for 1 h, and subsequent treatment with potassium hexafluorophosphate, afforded 3-butyl-1-mesityl-1*H*-imidazole-3-ium hexafluorophosphate (99) in 73% yield (Scheme 33).^70^

**Scheme 34:**
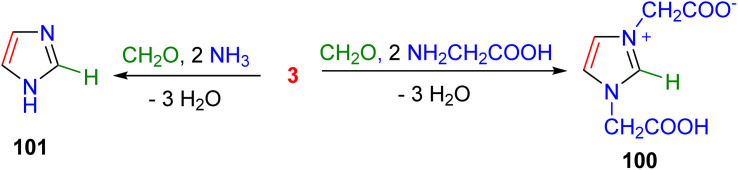
Formation of imidazole (101) and 3-carboxymethyl-1-imidazoliumethanoate (100).

In 2005, Velisek *et al.* reported the synthesis of 3-carboxymethyl-1-imidazoliumethanoate (1,3-bis(carboxymethyl)imidazole) (100) *via* the reaction of glyoxal with glycine under acidic, neutral, or alkaline conditions. The highest yield was obtained in mildly acidic media. In significantly lower amounts (on the order of a few ppm), imidazole (101) and trace levels of 4(5)-methylimidazole were also detected. It is worth noting that Strecker degradation of glycine produces formaldehyde, aminoacetaldehyde, methylamine, ammonia, and other compounds (Scheme 34).^71^

**Scheme 35:**
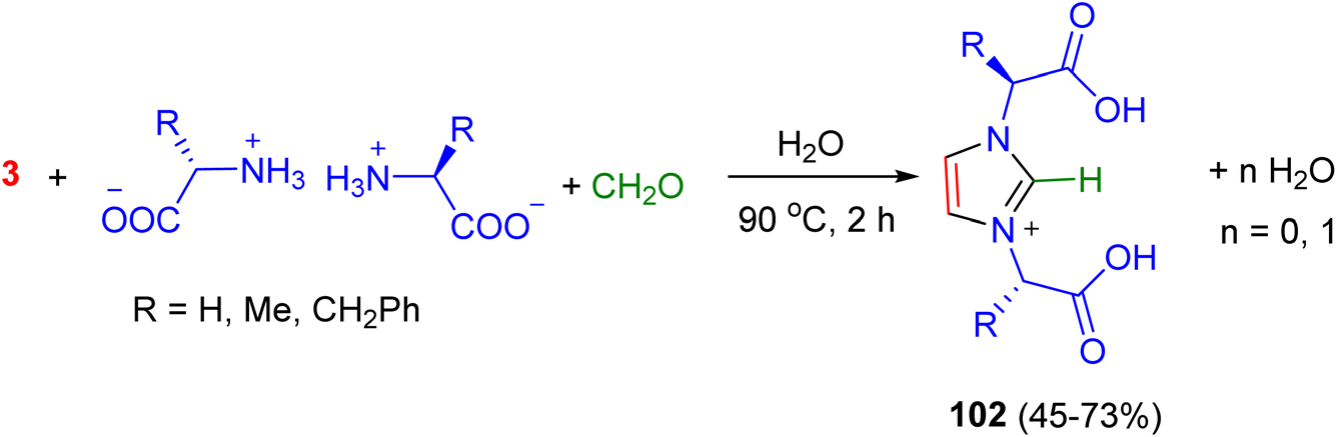
Synthesis of the double carboxylic acid-functionalised zwitterionic imidazolium 102.

Kuhl and Palm synthesized chiral, double carboxylic acid-functionalized imidazolium zwitterions 102 in 45–73% yields from the reaction of l-amino acids (*e.g.*, phenylalanine, alanine and glycine (achiral)) with paraformaldehyde and glyoxal in water at 95 °C for 2 h (Scheme 35).^72^

**Scheme 36:**
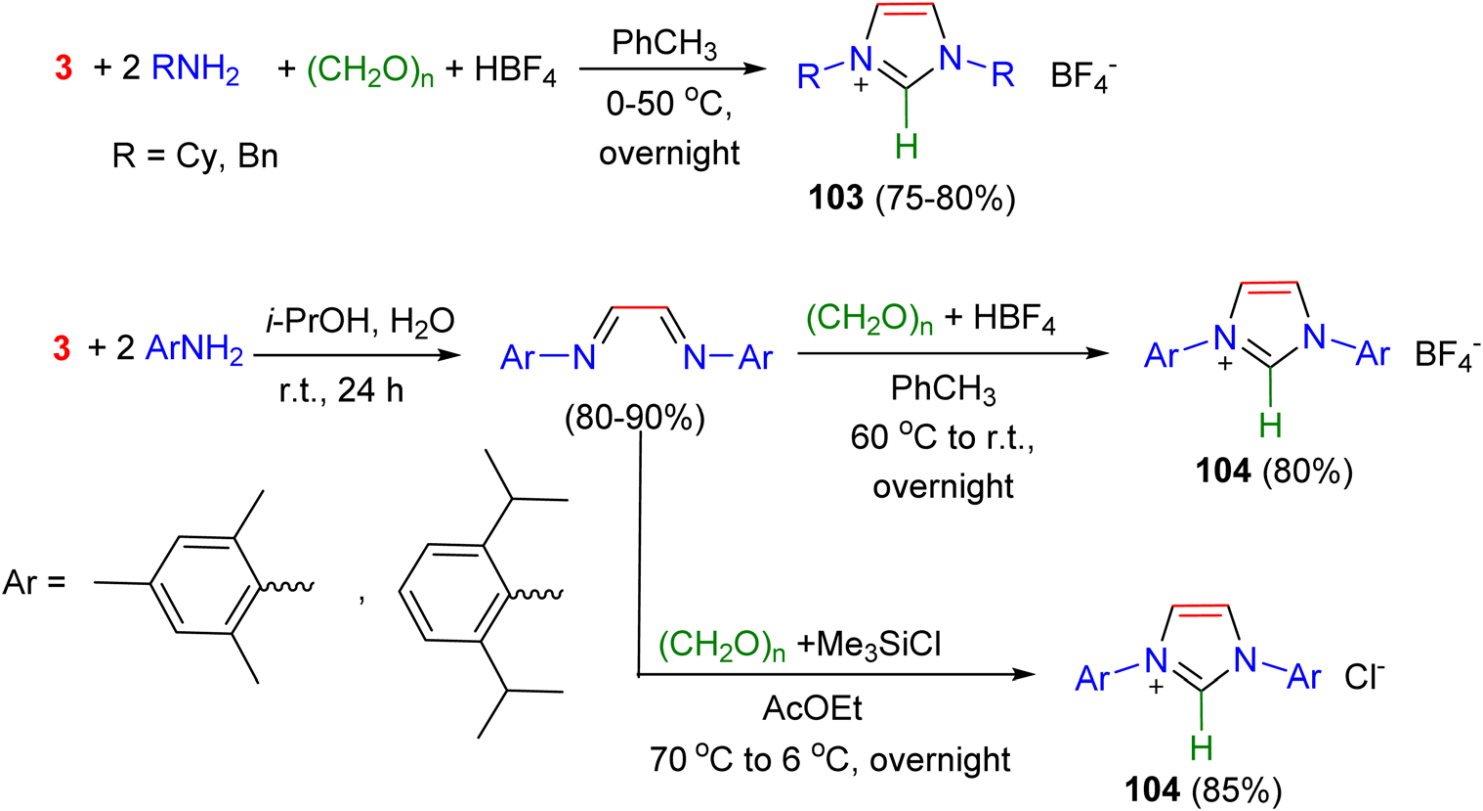
Synthesis of 1,3-dialkyl/diarylimidazolium tetrafluoroborates 103 and 104.

The one-pot condensation of glyoxal, two equivalents of a primary alkylamine, and paraformaldehyde in the presence of aqueous HBF_4_ in toluene at 0–50 °C overnight, provided straightforward access to symmetrical 1,3-dialkylimidazolium tetrafluoroborates 103 in 75–80% yields. To achieve the preparation of 1,3-diarylimidazolium salts 104, it was necessary to isolate the intermediate diimines prior to their cyclization. Although this additional step required more time and reagents, it led to a much more efficient overall process (Scheme 36).^73^

**Scheme 37:**
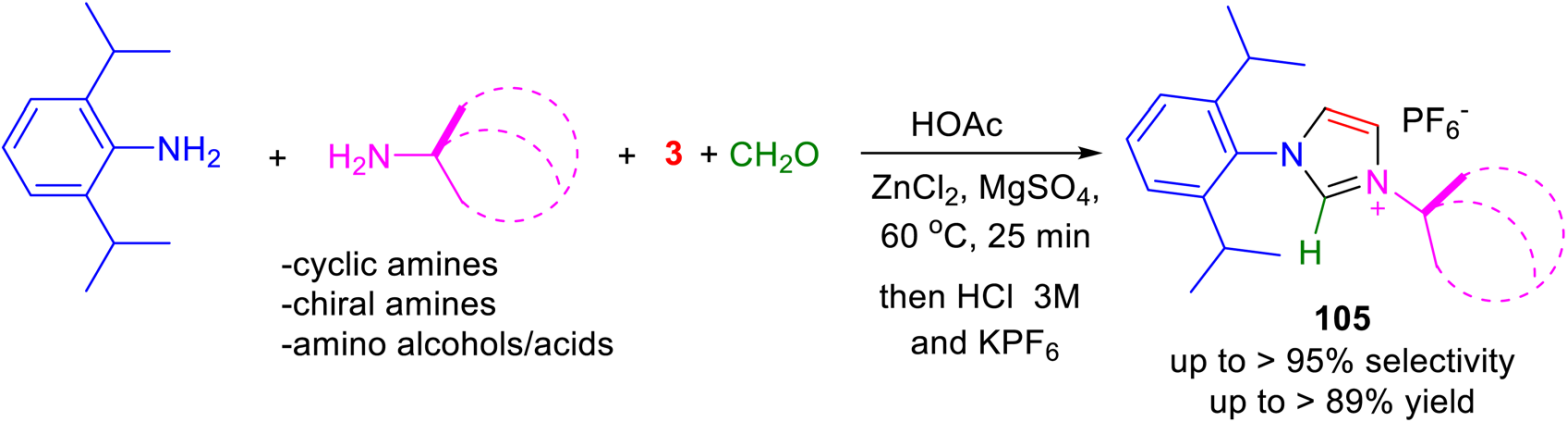
Synthesis of the unsymmetrical unsaturated 2,6-diisopropylphenyl-N-heterocyclic carbene ligands 105.

A multi-component reaction was employed to synthesize bulky unsymmetrical unsaturated 2,6-diisopropylphenyl-*N*-heterocyclic carbene (NHC) precursors (105) in 37–89% yields with excellent selectivity (up to 95%). The reaction involved the condensation of 2,6-diisopropylaniline, cyclic/chiral amines, amino alcohols, amino acids, glyoxal, and formaldehyde in acetic acid (HOAc), catalyzed by ZnCl_2_ and MgSO_4_ at 60 °C for 25 minutes. This method provides efficient access to novel chiral NHC ligands, which have demonstrated successful applications in copper-catalyzed asymmetric allylic alkylation and copper-catalyzed asymmetric borylation reactions (Scheme 37).^74^

**Scheme 38:**
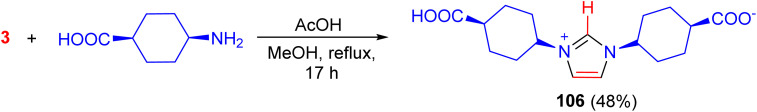
Serendipitous formation of a zwitterionic imidazolium 106.

In 2021, Pampaloni and co-workers described the synthesis of zwitterionic imidazolium molecule 106 in 48% yield through the reaction of 4-aminocyclohexanecarboxylic acid with glyoxal, using acetic acid as a catalyst in methanol under reflux conditions for 17 h. To build the five-membered ring, glyoxal plays the double role of source for C2 and, unusually, C1 units, the latter *via* thermal decomposition of glyoxal into CO and formaldehyde (CH_2_O), which, in turn, is able to guarantee the cyclization process (Scheme 38).^75^

#### 4.1.6. Synthesis of bis-imidazoles

**Scheme 39:**
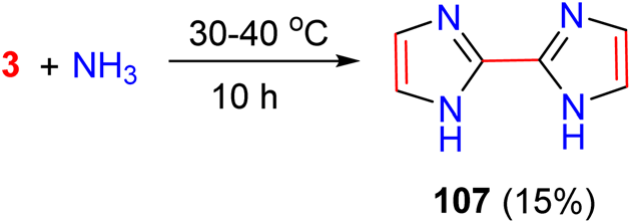
Synthesis of 2,2′-biimidazole 107.

In 2002, Teixido and coworkers reported a low-yielding synthesis of 2,2′-biimidazole (107) from the reaction of glyoxal (20% aqueous solution) with ammonia at 30–40 °C for 10 h. It should be noted that the synthesis of this compound had already been reported in 1987 by Kirchner *et al.* (Scheme 39).^76,77^

**Scheme 40:**
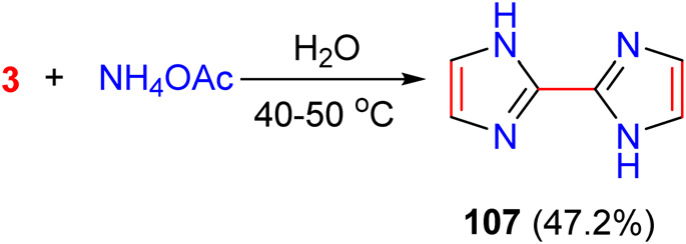
Synthesis of 2,2′-biimidazole (107).

After that, Paraskos *et al.* described the synthesis of 2,2′-biimidazole (107) in 47.2% yield by adding glyoxal to an aqueous solution of ammonium acetate under continuous stirring. The reaction was carried out over 5 hours, with the exothermic process maintaining the temperature between 40–50 °C throughout the addition (Scheme 40).^78^

**Scheme 41:**
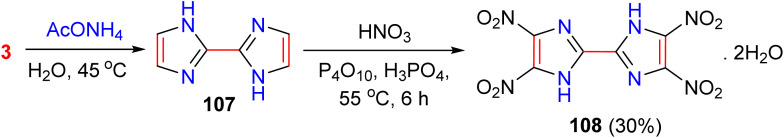
Synthesis of the biimidazole derivatives 107 and 108.

Next, Lewczuk *et al.* produced 2,2′-biimidazole (107) from ammonium acetate and aqueous glyoxal in water at 45 °C. Nitration of 107 with nitric acid in a mixture of phosphoric acid and phosphorus(v) oxide, followed by recrystallization from water, afforded tetranitrobiimidazole dihydrate (108) in 30% yield, free of sulfate impurities. The tetranitrobiimidazole was then converted into the corresponding semicarbazidium, triazolium, and tetrazolium derivatives (Scheme 41).^79^

#### 4.1.7. Synthesis of benzofurans and naphthofurans

**Scheme 42:**
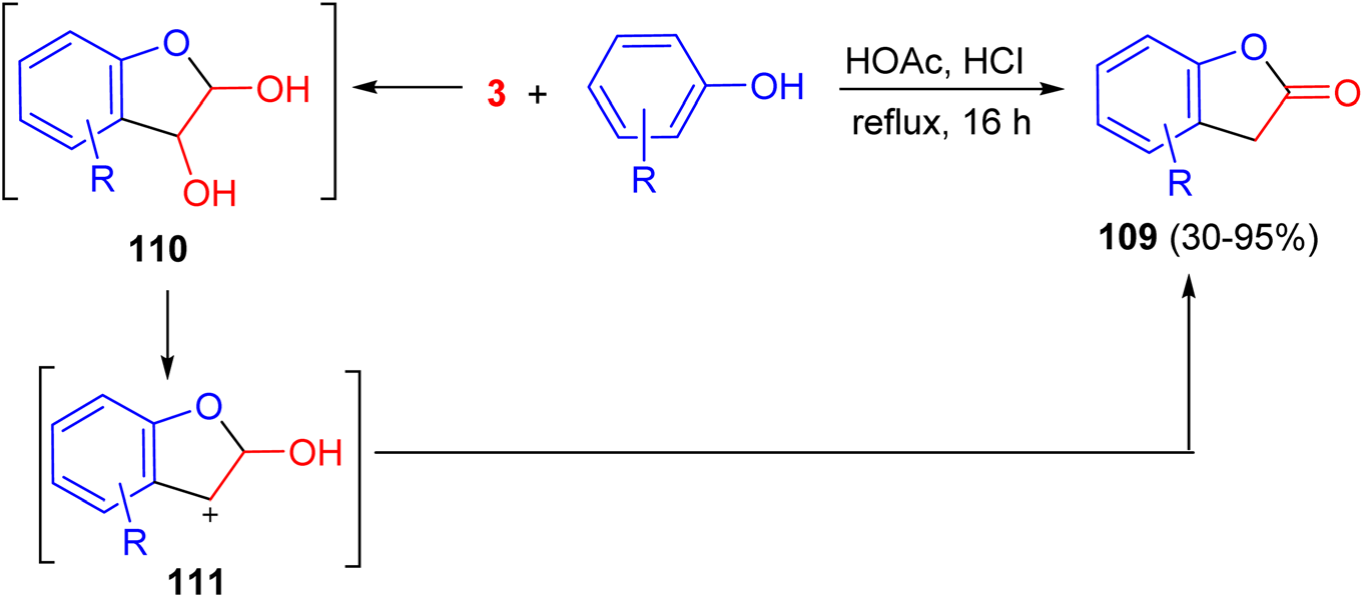
Acid-catalyzed synthesis of 2(3*H*)benzofuranones 109.

In 1975, Layer synthesized 2(3*H*)benzofuranones 109 in 30–95% yields by the reaction of phenol derivatives with glyoxal in refluxing glacial acetic acid with HCl or *p*-TSOH as the catalyst for 16 h. In the proposed mechanism, non-isolable 2,3-dihydroxy-2,3-dihydrobenzofuran 110 was formed in the rate-determining step, which is dehydroxylated to 111. This rapidly loses a proton to give the desired product (Scheme 42).^80^

**Scheme 43:**
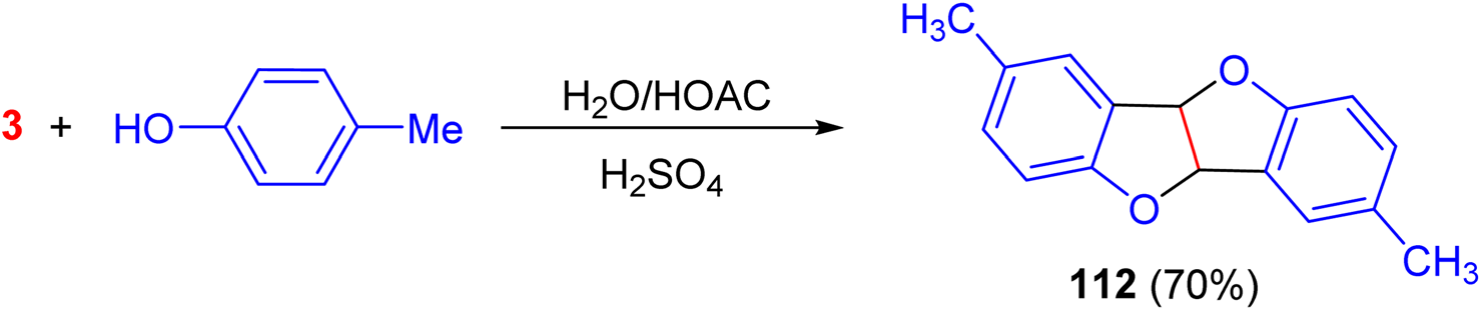
H_2_SO_4_-catalyzed synthesis of 5,5′-dimethyl-coumarano-3′,2′,2,3-coumaran (112).

5,5′-Dimethyl-coumarano-3′,2′,2,3-coumaran (112) was synthesized in 70% yield by reacting equimolar amounts of glyoxal monohydrate and *p*-cresol. The reaction was heated in an aqueous acetic acid solution and catalyzed by sulfuric acid. Repeated attempts to condense glyoxalmonohydrate with *p*-cresol using *p*-toluenesulphonic acid as a catalyst at 102 °C for 14 h, fusion of the same reactants at 130–140 °C for 10 minutes, and condense anhydrous glyoxal with *p*-cresol in bis-(2-ethoxyethyl)ether using *p*-toluenesulphonic acid as a catalyst at 0 °C for 24 h, were unsuccessful (Scheme 43).^81^

**Scheme 44:**
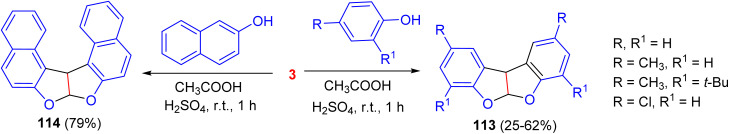
Synthesis of acetals 113 and 114.

The acid-catalyzed reaction of glyoxal at the *ortho*-position of phenols such as phenol, 2-naphthol, *p*-cresol, 2-*t*-butyl-4-methylphenol and *p*-chlorophenol in acetic acid at room temperature for 1 h, afforded the corresponding acetals 113 and 114 in 25–79% yields (Scheme 44). The structures identified by NMR analysis were confirmed in several cases through synthesis *via* an unambiguous route.^82^

**Scheme 45:**
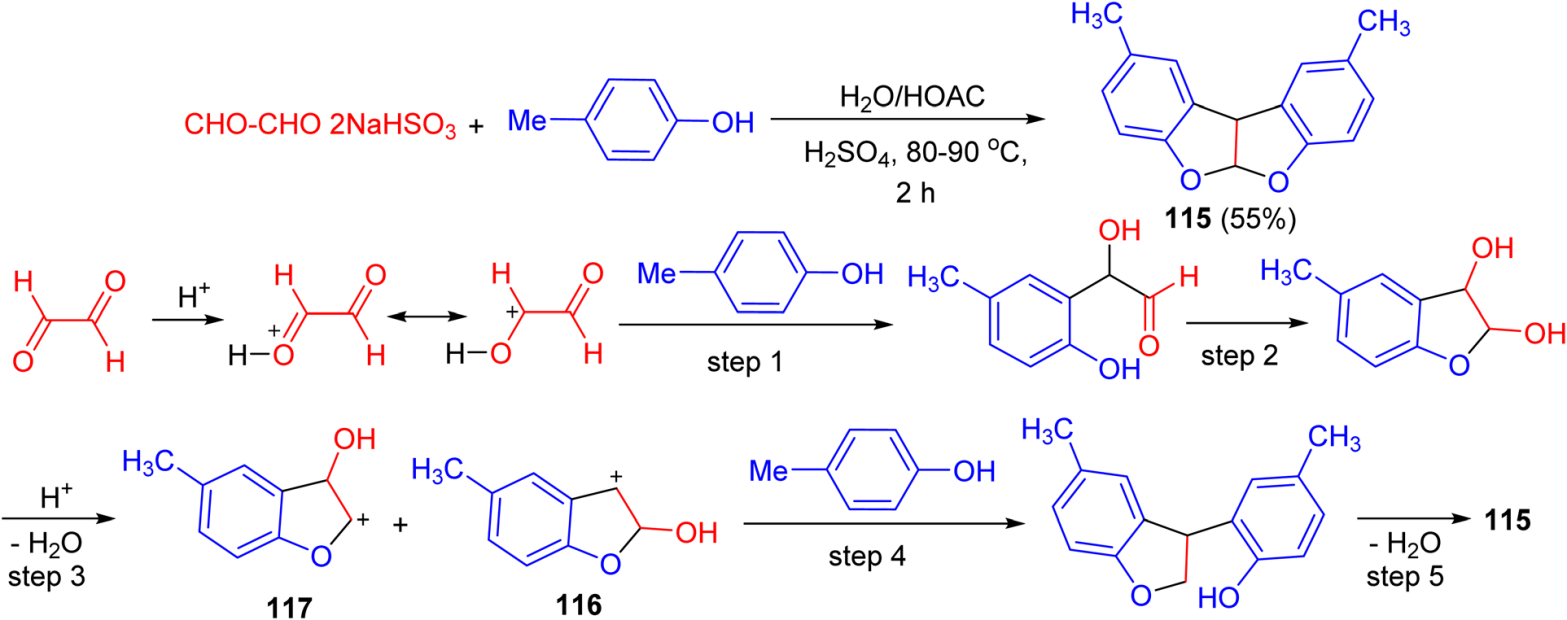
Preparation of 2,9-dimethyl (5*a*, 10*b*)dihydrobenzofuro(2,3-*b*)benzofuran (115).

Next, 2,9-dimethyl (5*a*,10*b*)dihydrobenzofuro(2,3-*b*)benzofuran (115) was produced in 55% yield by reaction of glyoxal bisulphite and *p*-cresol in H_2_O/HOAc using H_2_SO_4_ as a catalyst at 80–90 °C for 2 h. A possible mechanism is illustrated in Scheme 45. An electrophilic substitution on the phenolic substrate by protonated glyoxal must undoubtedly be the first step (step 1). Step 2 is an acetal-forming reaction of phenolic-OH with the second free aldehyde group on the glyoxal, which, by subsequent dehydrogenation, will lead to 116 (step 3). Based on resonance theory, 117 has less stability than 116 (116 is a benzylic cation). The reaction then follows step 4, the electrophilic attack of the cation 116 on the *ortho*-position of the second phenol molecule. Finally, a dehydration reaction produces the product 115 in the last step (step 5).^83^

**Scheme 46:**
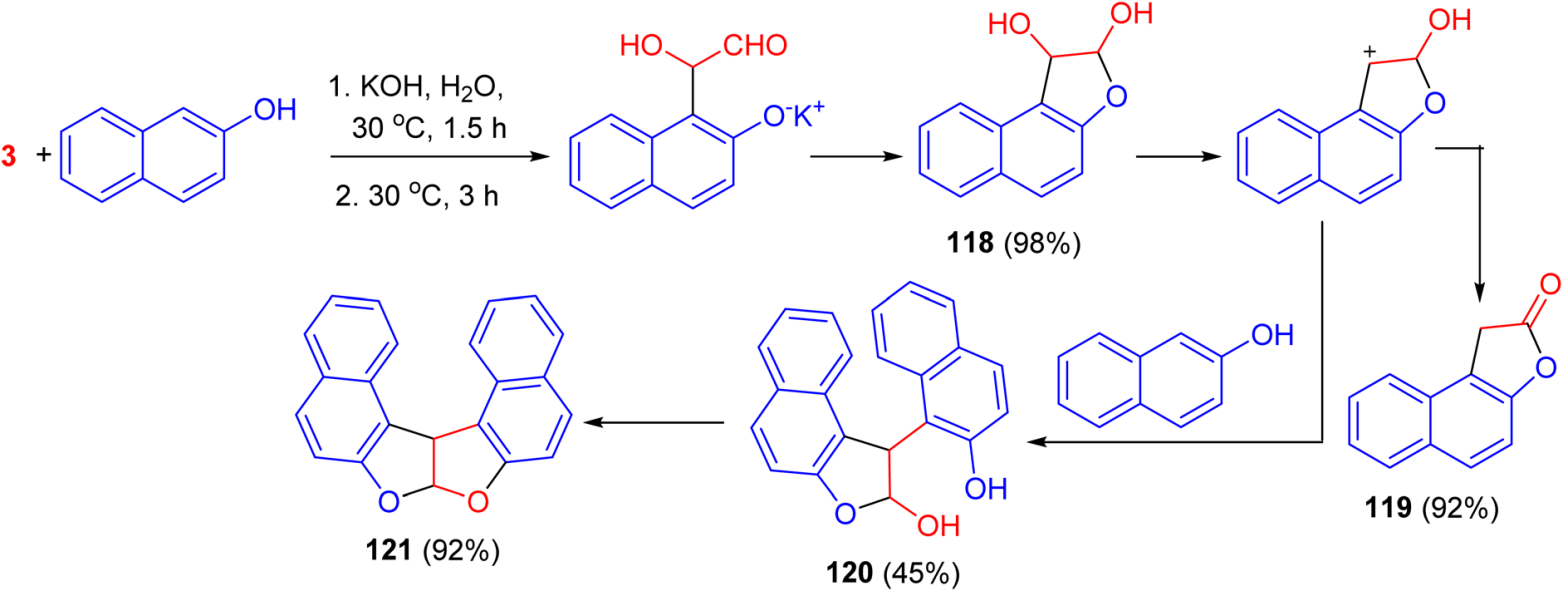
Base-catalyzed preparation of the naphthofuran derivatives 118–121.

Kito and their team successfully developed the alkylation of potassium 2-naphthyl oxide with glyoxal in aqueous media, THF and CHCl_3_. This approach resulted in the formation of 1,2-dihydronaphtho[2,1-*b*]furan-1,2-diol (118) in 98% yield. Without isolation of 118, acidification of this reaction mixture with aqueous HCl led to three products, *i.e.*, naphtho[2,1-*b*]furan-2(1*H*)-one (119) in 92% yield, the hemiacetal of bis(2-hydroxy-lnaphthy1)acetaldehyde (120) in 45% yield, and the corresponding acetal (121) in 92% yield (Scheme 46).^84^

**Scheme 47:**
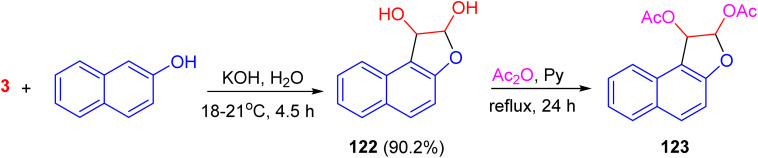
Synthesis of the 1,2-dihydronaphtho[2,1-*b*]furan derivatives 122 and 123.

In 1994, Kito *et al.* reported that the monohydrate of *cis*/*trans*-1,2-dihydronaphtho[2,1-*b*]furan-l,2-diol 122 was obtained *via* treatment of 2-naphthol with aqueous glyoxal (40%) using KOH at 18–21 °C for 4.5 h in 90.2% yield. Furthermore, treatment of 122 with acetic anhydride and pyridine under reflux for 24 h afforded *cis*/*trans*-1,2-diacetoxy-1,2-dihydronaphtho[2,1-*b*]furan (123) in nearly quantitative yield (Scheme 47).^85^

**Scheme 48:**
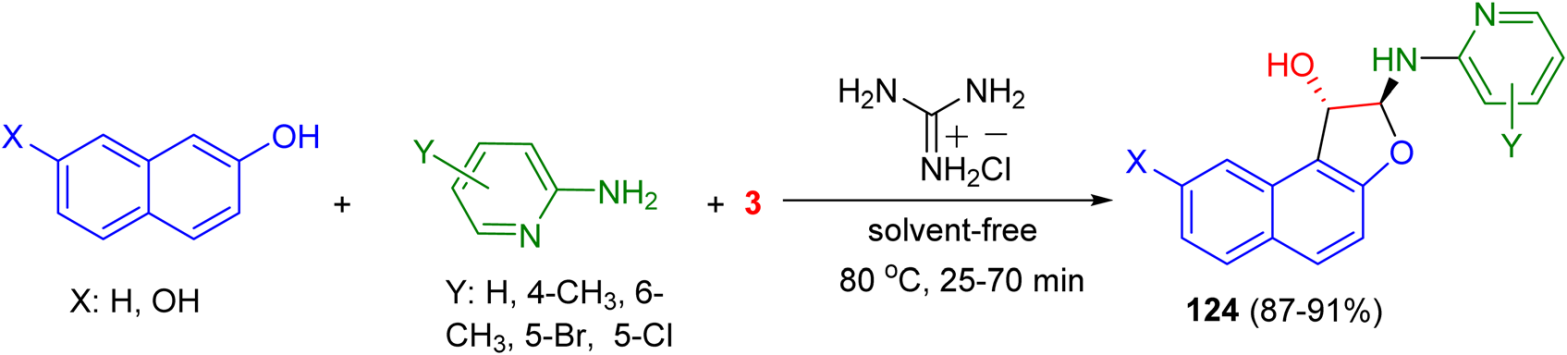
Guanidinium chloride catalyzed synthesis of 1,2-dihydronaphtho[2,1-*b*]furans 124.

A simple and highly efficient one-pot protocol for the synthesis of polysubstituted *trans*-1,2-dihydronaphtho[2,1-*b*]furans 124 in 87–91% yields has been developed using the three-component coupling reaction of 2-aminopyridines, naphthols and aq. glyoxal, in the presence of guanidinium chloride as a polyfunctional organocatalyst, under solvent-free conditions at 80 °C for 25–70 min (Scheme 48).^86^

#### 4.1.8. Synthesis of the other five-membered heterocycles

**Scheme 49:**
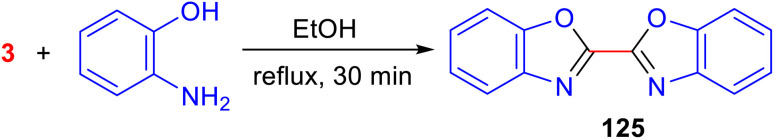
Synthesis of 2,2′-bibenzoxazoline (125).

In 1959, Murase reported the synthesis of 2,2′-bibenzoxazoline (125) by the condensation reaction of *o*-aminophenol with glyoxal in EtOH under reflux conditions for 30 min, as shown in Scheme 49.^87^

**Scheme 50:**
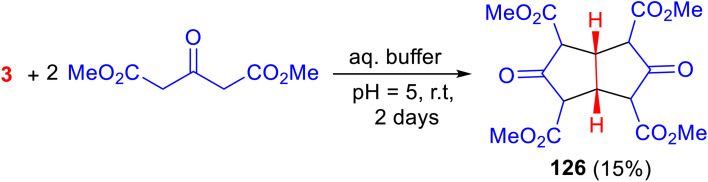
Synthesis of bicyclic compound 126.

Weiss and Edwards produced tetramethyl *cis*-bicyclo[3.3.0]octane-3,7-dione-2,4,6,8-tetracarboxylate 126 in 15% yield through the reaction of dimethyl 1,3-acetonedicarboxylate with glyoxal in aqueous buffer at pH 5 at room temperature for 2 days (Scheme 50).^88^

**Scheme 51:**
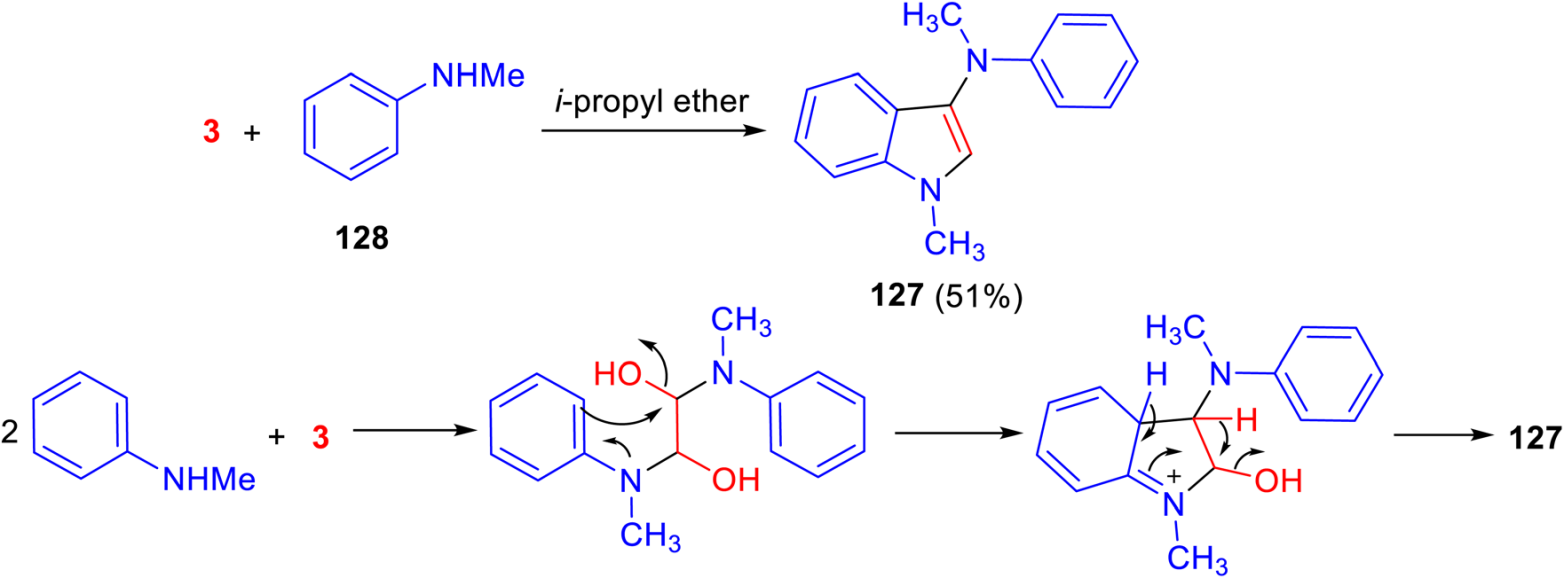
Synthesis of l-methyl-3-(methylphenylamino)indole (127).

After that, synthesis of l-methyl-3-(methylphenylamino)indole (127) in 51% yield was reported by the reaction of *N*-methylaniline (128) with glyoxal in isopropyl ether under heating. The suggested mechanism is outlined in Scheme 51.^89^

**Scheme 52:**
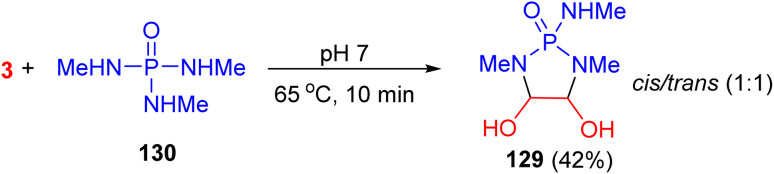
Synthesis of the 1,3,2-diazaphospholidine five-membered ring system 129.

**Scheme 53:**
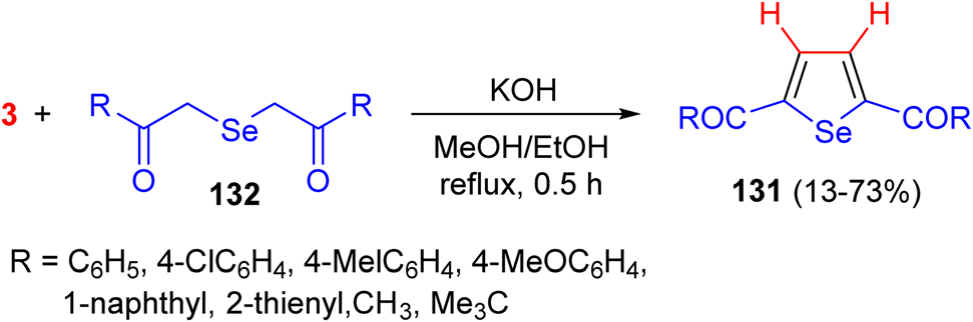
Synthesis of 2,5-diacylselenophenes 131.

In 1977, the 1,3,2-diazaphospholidine five-membered ring system (129) was synthesized in 42% yield by reacting *N*,*N*′,*N*″-trimethylphosphoric triamide (130) with aqueous glyoxal at 65 °C for 10 minutes, using a 50% sodium hydroxide solution to adjust the pH to 7 (Scheme 52). Despite the simplicity of this synthesis, similar attempts with other phosphoramides failed to yield isolable cyclic products.^90^In 1987, Nakayama *et al.* prepared 2,5-diacylselenophenes 131 in 13–73% yields through the base-catalyzed condensation of α,α′-diketo selenides 132 with glyoxal in MeOH/EtOH under reflux conditions for 0.5 h (Scheme 53).^91^

**Scheme 54:**
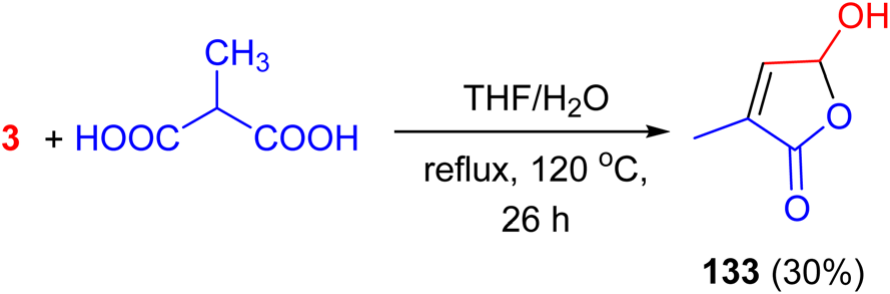
Acid-catalyzed preparation of 5-hydroxy-3-methyl-2(5*H*)-furanone (133).

In 2010, 5-hydroxy-3-methyl-2(5*H*)-furanone (133) was synthesized in 30% yield *via* an acid-catalyzed aldol-type condensation. The reaction between glyoxal and methylmalonic acid was conducted in THF/H_2_O at 120 °C under reflux for 26 h. This furanone is the substructure in natural and synthetic strigolactones, which are germination stimulants for seeds of the parasitic weeds Striga and Orobanche spp. (Scheme 54).^92^

**Scheme 55:**
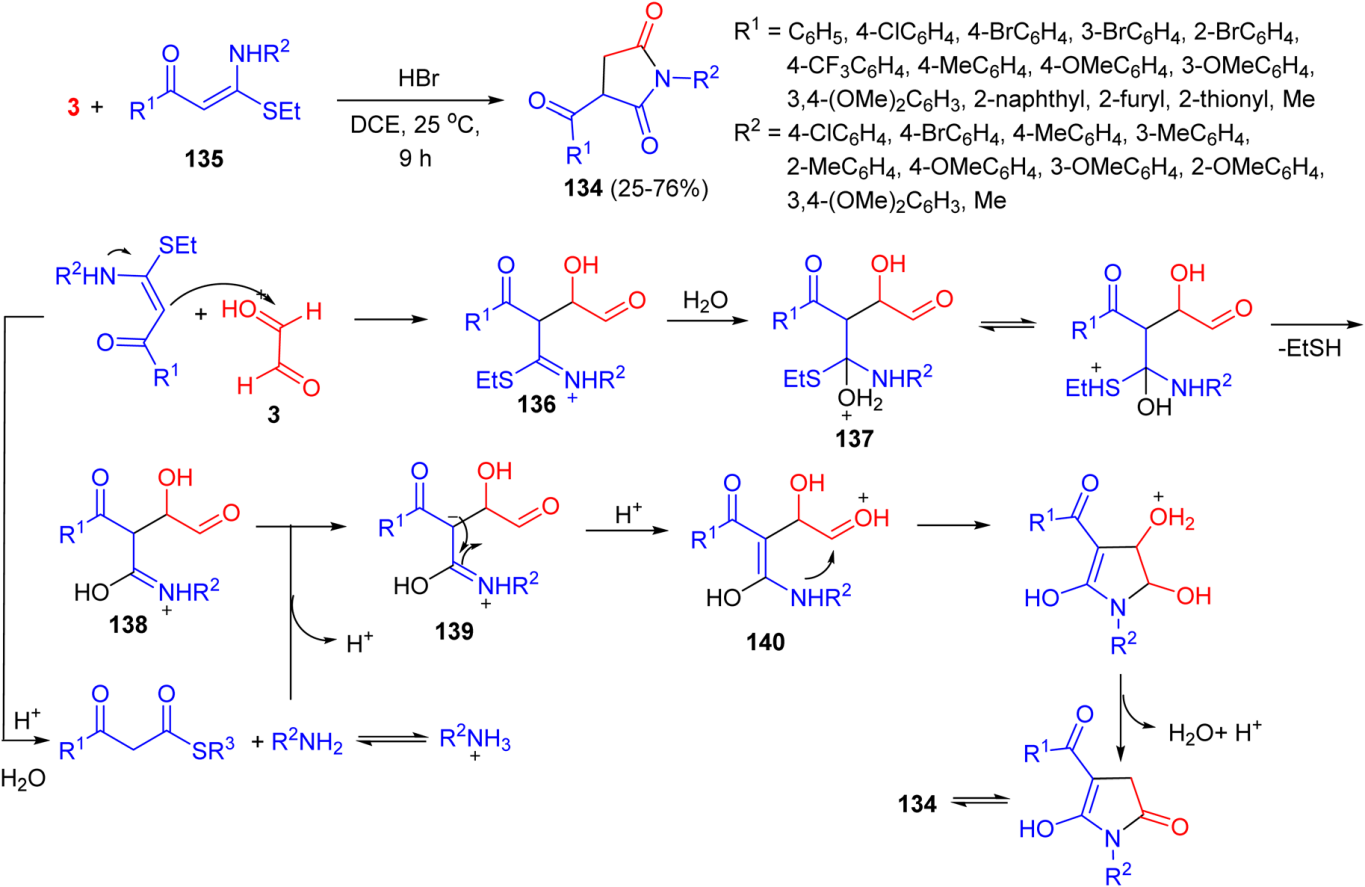
Synthesis of α-substituted succinimides 134.

In 2019, Li and co-workers described the synthesis of α-ketosuccinimides 134 in 25–76% yields by the reaction of ketene *N*,*S*-acetals 135 with glyoxal in the presence of HBr in dichloroethane at room temperature for 9 h. A plausible mechanism is proposed in Scheme 55. Initially, the nucleophilic attack of the electron-rich α-carbon atom of 135 at the activated carbonyl carbon of glyoxal (enamine addition) should occur to form the iminium intermediate 136. Then, nucleophilic attack of H_2_O at the iminium carbon of 136 gives intermediate 137, which converts into iminium intermediate 138 through release of the alkylthio group. Promoted by the amine released from the formation of the β-ketothioester, deprotonation of 138 can lead to intermediate 139, which then forms the *N*,*S*-acetal intermediate 140. Subsequently, intramolecular nucleophilic addition (cyclization) of the amino at the activated aldehyde carbon of 140 takes place. Finally, α-ketosuccinimide is constructed by loss of a molecule of H_2_O and deprotonation.^93^

**Scheme 56:**
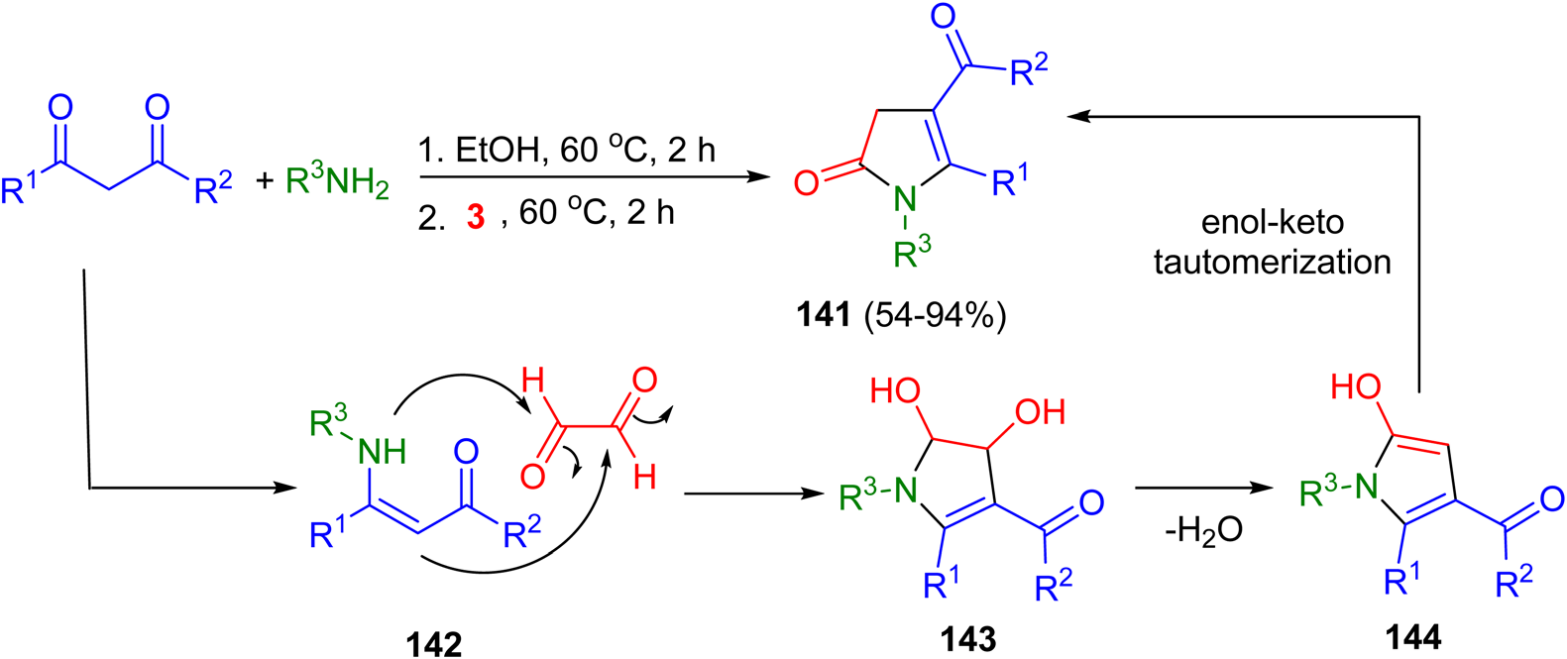
Synthesis of 2-pyrrolin-5-ones 141.

Tang and his group successfully developed an effective approach for the green synthesis of 2-pyrrolin-5-ones 141 in 54–94% yields, using a one-pot cyclization between 1,3-diketones, amines, and bio-renewable glyoxal in EtOH at 60 °C for 4 h. In this environment-benign transformation, water is the only byproduct. A wide range of substrates was tolerated and afforded the corresponding products in moderate to high yields. The proposed mechanism is outlined in Scheme 56. Firstly, the condensation of dicarbonyl compounds and amines gives enaminone intermediate 142. Then, this 1,3-dinucleophilic compound 142 reacts with glyoxal to afford intermediate 143, by a twofold nucleophilic addition. The follow-up dehydration of 143 provides intermediate 144, which is transformed into the final product 141*via* an enol–keto tautomerization.^94^

### 4.2. Synthesis of six-membered heterocycles

#### 4.2.1. Synthesis of piperazines

**Scheme 57:**
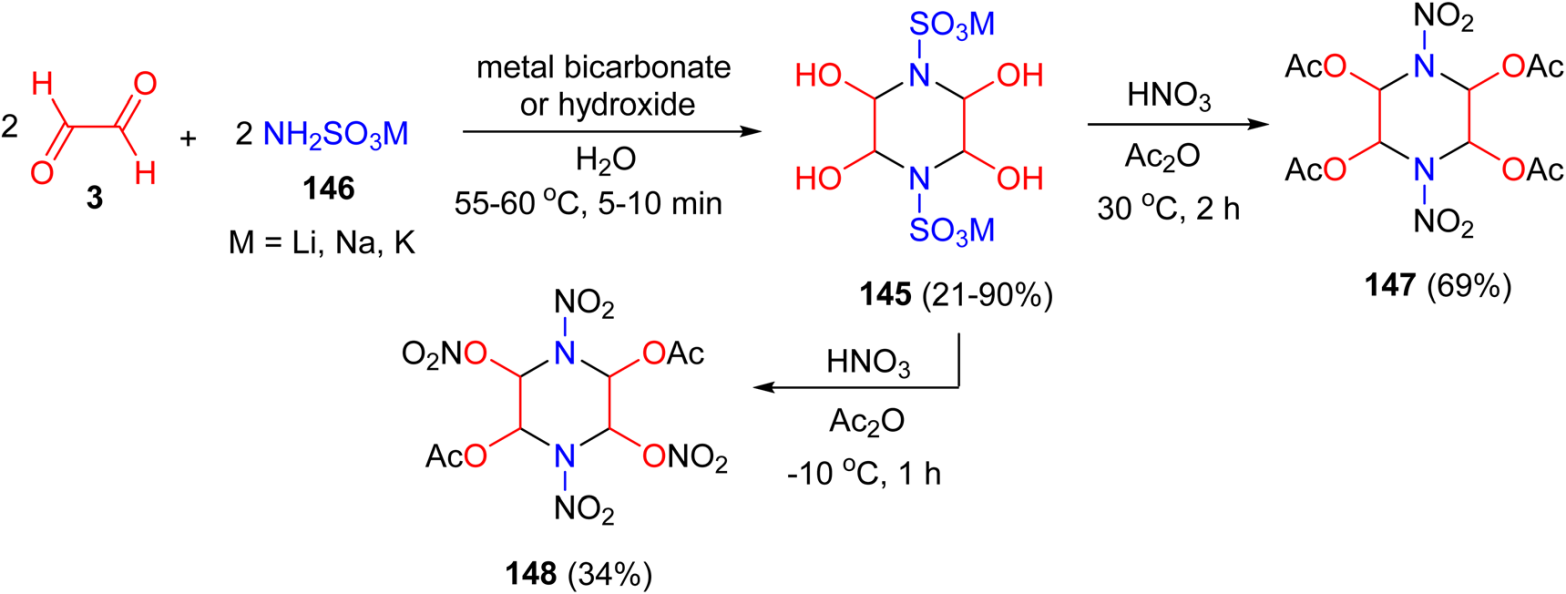
Synthesis of highly substituted piperazines 145, 147 and 148.

In 1967, the Dinwoodie group synthesized 2,3,5,6-tetrahydroxypiperazine-1,4-disulphonic acid salts 145 in 21–90% yields by the addition of aqueous glyoxal to sulphamic acid (146) in the presence of base in water at 55–60 °C in 5–10 min. The free acid was not isolated. 2,3,5,6-tetra-acetoxy-1,4-dinitropiperazine (147) and 2,5-diacetoxy-3,6-dinitrato-l,4-dinitropiperazine (148) were prepared by the action of nitric acid and acetic anhydride on the tetrahydroxypiperazine salts (Scheme 57).^95^

**Scheme 58:**
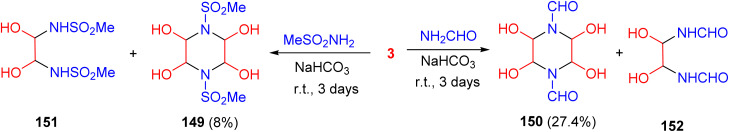
Synthesis of the 2,3,5,6-tetrahydroxypiperazine derivatives 149 and 150.

**Scheme 59:**
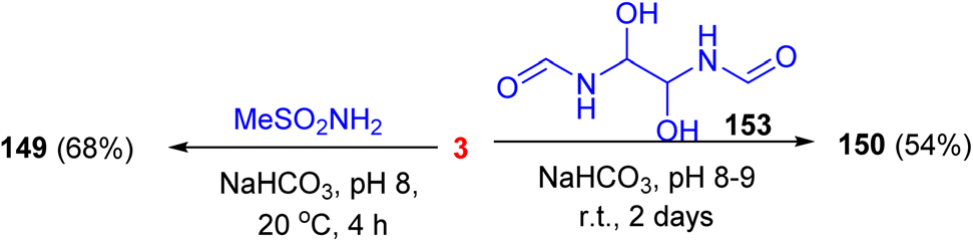
Base-catalyzed synthesis of 2,3,5,6-tetrahydroxypiperazines 149 and 150.

The reaction of glyoxal with formamide and methanesulfonamide under basic conditions at room temperature for 3 days provided a mixture of products (Scheme 58). The 2,3,5,6-tetrahydroxypiperazine derivatives 149 and 150 were isolated in yields of 8% and 27.4%, respectively. The corresponding *N*,*N*′-disubstituted 1,2-diamino-1,2-ethanediols 151 and 152 were also formed in this process.^96^ The same group also synthesized compounds 149 and 150 under basic conditions (pH 8–9, NaHCO_3_). Notably, employing 1,2-diformamido-1,2-ethanediol (153) as a precursor in the reaction with glyoxal under these same aqueous conditions at room temperature for 2 days significantly improved the yield of 150 to 54%. Similarly, methane sulfonamide reacted with glyoxal in water (pH 8.0, NaHCO_3_) at 20 °C for 4 h to afford 2,3,5,6-tetrahydroxy-1,4-bismethylsulfonylpiperazine (149) in 68% yield. The structures of these tetrahydroxy compounds were confirmed through derivatization into esters, ethers, and chloro-derivatives (Scheme 59).^96^

**Scheme 60:**
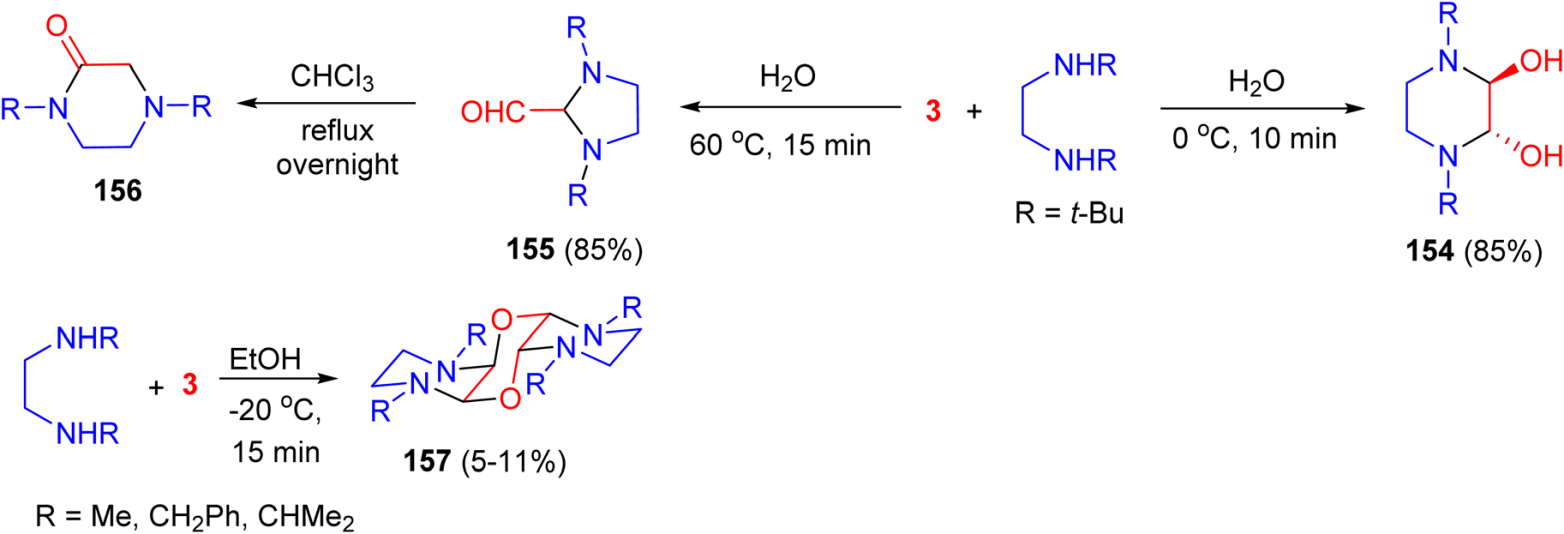
Synthesis of imidazolidine 155, piperazines 154, 156 and tricyclic compound 157.

In 1985, Willer and coworkers developed new chemistry from the reaction of *N*,*N*′-disubstituted ethylenediamines with glyoxal. In this method, the reaction of *N*,*N*′-di-*tert*-butylethylenediamine with glyoxal in water at 0 °C for 10 min gives initially *trans*-2,3-dihydroxy-1,4-di-*tert*-butylpiperazine 154, which rearranges thermally to 1,3-di-*tert*-butyl-2-imidazolidinecarboxaldehyde 155 at 60 °C for 5 min, and then compound 155 is converted into 1,4-di-*tert*-butyl-2-ketopiperazine 156 in CHCl_3_ at reflux overnight. The reaction of a series of *N*,*N*′-dialkyl-substituted ethylenediamines with glyoxal in ethanol at low temperature (−20 °C) for 15 min has been found to give a series of *cis*–*trans*–*cis*-1,4,6,9-tetraalkyl-1,4,6,9-tetraaza-5,10-dioxaperhydroanthracens 157 as minor products (Scheme 60).^97^

**Scheme 61:**
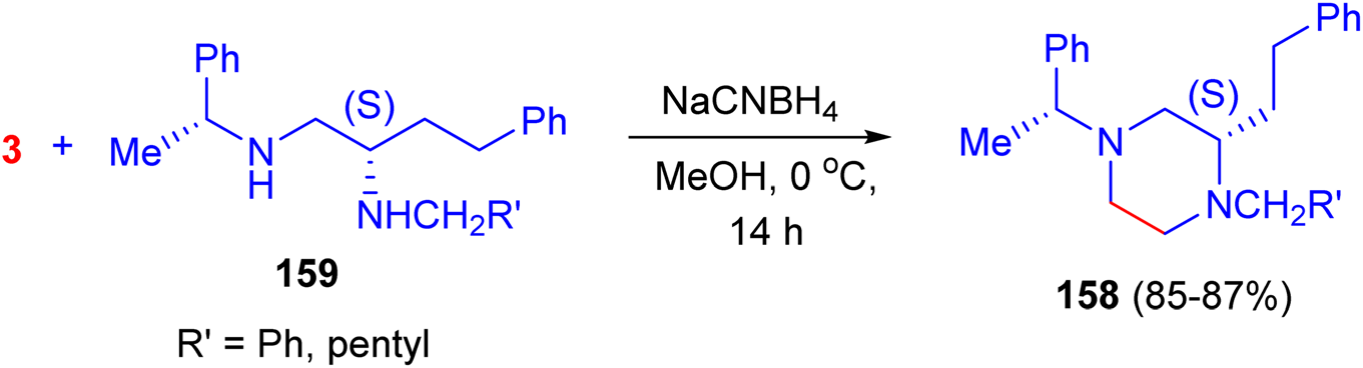
Synthesis of 2-substituted-1,4-piperazines 158.

Lee and his team synthesized 2-substituted-1,4-piperazines 158 in 85–87% yields *via* reductive cyclization of 1-(1′*R*)-phenethyl-2-(*S*)-alkylamino-1,2-diamine (159) with 40% aqueous glyoxal in methanol at 0 °C for 14 hours, using NaCNBH_3_ as the reducing agent (Scheme 61).^98^

#### 4.2.2. Synthesis of quinoxalines

**Scheme 62:**
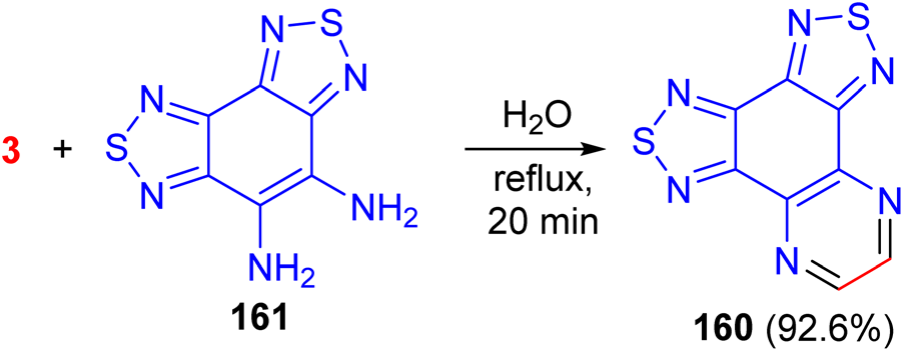
Synthesis of bis[1,2,5]thiadiazolo[3,4-*f*:3′,4′-*h*]quinoxaline (160).

Bis[1,2,5]thiadiazolo[3,4-*f*:3′,4′-*h*]quinoxaline (160) was synthesized in 92.6% yield by cyclization of diamine 161 with aqueous glyoxal (40%) in water under reflux conditions for 20 minutes, as shown in Scheme 62.^99^

**Scheme 63:**
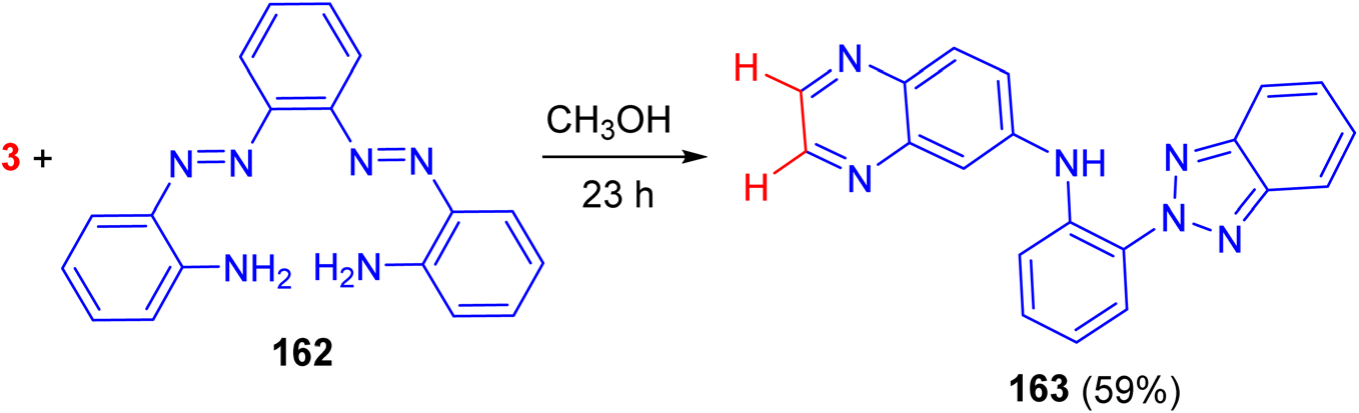
Synthesis 2-(2-2*H*-benzotriazolyl)-*N*-(6-quinoxalinyl)aniline 163.

In 1976, Skrabal and his group reported the cyclization of diamine 162 with 30% aqueous glyoxal in methanol (23 h), giving the rearranged product 163 in 59% yield (Scheme 63).^100^

**Scheme 64:**
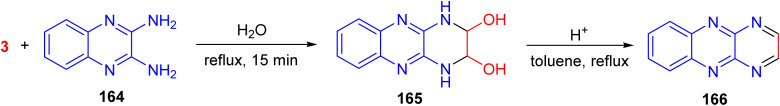
Synthesis of pyrazino[2,3-*b*]quinoxaline (166).

The reaction of 2,3-diaminoquinoxaline (164) with 40% aqueous glyoxal in water under reflux for 15 min resulted 1,2,3,4-tetrahydro-2,3-dihydroxypyrazino[2,3-*b*]quinoxaline (165). Compound 166 was also prepared by dehydration of 165 in toluene with a catalytic amount of *p*-toluenesulfonic acid and azeotropic removal of water. Also, when 165 was dehydrated by vacuum sublimation at high temperature, 166 was obtained (Scheme 64).^101^

**Scheme 65:**
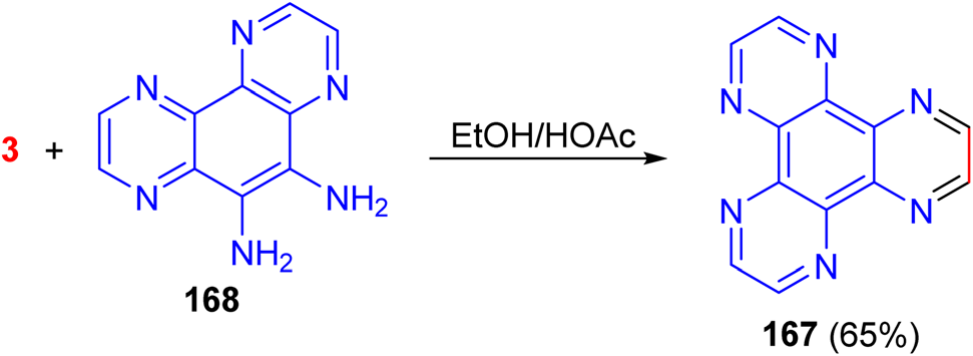
Synthesis of dipyrazino[2,3-*f*][2′,3′-h]quinoxaline (167).

In 1981, Nasielski-Hinkens and his colleagues produced dipyrazino [2,3-*f*][2′,3′-*h*]quinoxaline (167), a new ligand for low-valent transition metals, in 65% yield by the reaction of diamine 168 with 30% aqueous glyoxal in the mixture of EtOH/HOAc after a few minutes. Additionally, mono-, bis-, and tris-chromium tetracarbonyl complexes were synthesized from compound 167 (Scheme 65).^102^

**Scheme 66:**
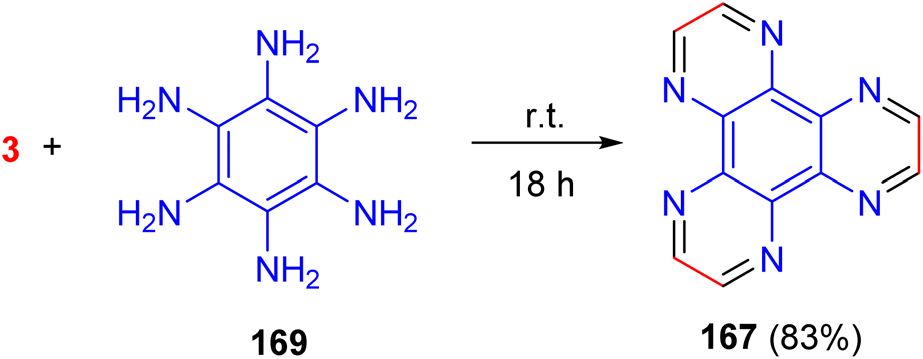
Preparation of 1,4,5,8,9,12-hexaazatriphenylene (167).

After that, Rogers synthesized 1,4,5,8,9,12-hexaazatriphenylene (167) in 83% yield by the reaction of hexaaminobenzene (169) with 40% aqueous glyoxal at room temperature for 18 h (Scheme 66).^103^

**Scheme 67:**
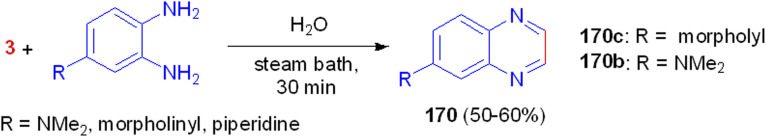
Synthesis of pyrido[2,3-*b*]pyrazines (170).

In 1982, Postovskii *et al.* published a report on the synthesis of 6-*R*-pyrido[2,3-*b*]pyrazines (170) in 50–60% yields *via* the cyclization of 6-*R*-2,3-diaminopyridines with 40% aqueous glyoxal in water under steam bath conditions for 30 minutes. The synthesized compounds were evaluated for their antitumor activity, with some demonstrating moderate efficacy. Specifically, compound 170c, in doses of 50–100 mg kg^−1^, exhibited the growth of sarcoma 37 by 50–55%, and compound 170b inhibited a 60% suppression of Lewis tumor growth (Scheme 67).^104^

**Scheme 68:**
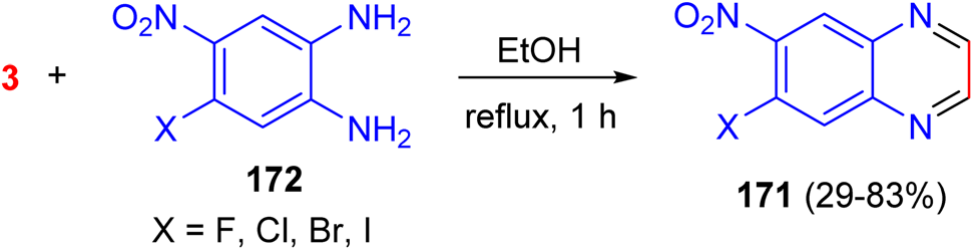
Synthesis of 6-fluoro-, 6-chloro- and 6-bromo-7-nitroquinoxaline 171.

Next, Nasielski-Hinkens and co-workers synthesized 6-fluoro-, 6-chloro- and 6-bromo-7-nitroquinoxaline 171 in 29–83% yields starting from 1,2-diamino-4-halobenzene 172 and glyoxal in EtOH under reflux conditions for 1 h (Scheme 68).^105^

**Scheme 69:**
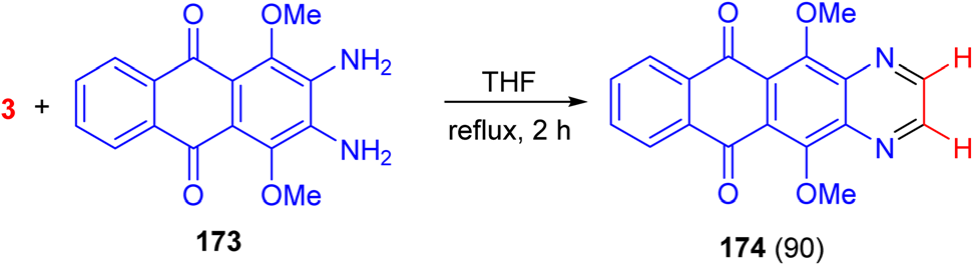
Preparation of naphtho[2,3-*g*]quinoxaline-6,11-dione derivatives 174.

In 2007, Shchekotikhin *et al.* reported that condensing diaminoanthraquinone 173 with glyoxal in refluxing THF for 2 h gave the naphtho[2,3-g]quinoxaline-6,11-dione derivatives 174 in 90% yield (Scheme 69).^106^

**Scheme 70:**
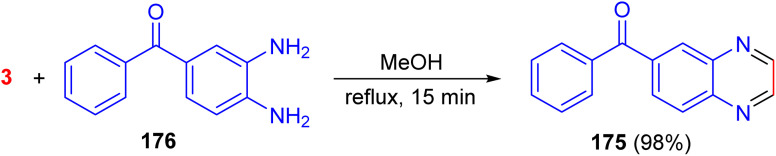
Preparation of 7-benzoyl quinoxaline (BQ) 175.

In 2023, 7-benzoyl quinoxaline (BQ) 175 was synthesized in 98% yield by the cyclocondensation of 3,4-diaminobenzophenone 176 with glyoxal in MeOH under reflux conditions for 15 min. The results of CDFT-based calculations for 175 showed that this molecule is stable and can act as a good electron acceptor. Molecular docking studies, providing valuable insights into the biological activity of the novel molecule, highlighted its potential to showcase promising therapeutic characteristics against commonly occurring cancer types (Scheme 70).^107^

#### 4.2.3. Synthesis of the other six-membered heterocycles

**Scheme 71:**
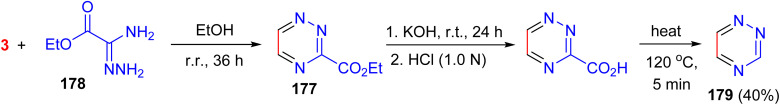
Synthesis of 1,2,4-triazine 179.

In 1966, Paudler and his team synthesized ethyl 1,2,4-triazine-3-carboxylate (177) by condensing glyoxal with ethyl oxalamidrazonate (178) in absolute ethanol at room temperature for 36 h. This intermediate was then subjected to saponification using potassium hydroxide, followed by dissolution in 1.0 N aqueous hydrochloric acid and decarboxylation at 120 °C, giving the final product, 1,2,4-triazine (179), in 40% yield (Scheme 71).^108^

**Scheme 72:**
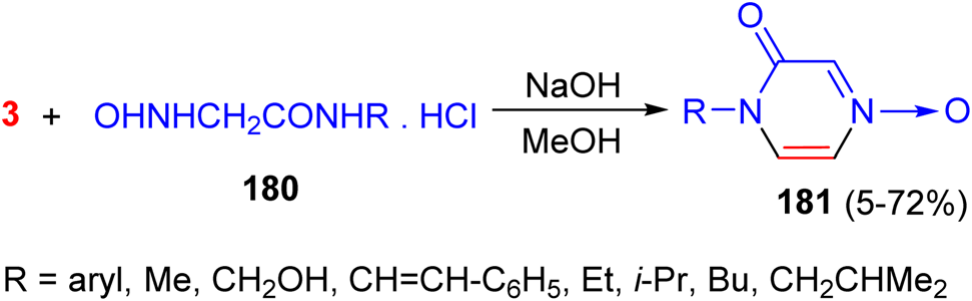
Synthesis of the 2(1*H*)-pyrazinone-4-oxide derivatives (181).

In 1980, Mano and coworkers reported the condensation of *N*-(alkyl- or aryl)-2-(hydroxyamino)acetamide hydrochlorides (180) with 40% aqueous glyoxal in methanol, using NaOH, to afford 2(1*H*)-pyrazinone-4-oxide derivatives (181) in 5–72% yields (Scheme 72).^109^

**Scheme 73:**
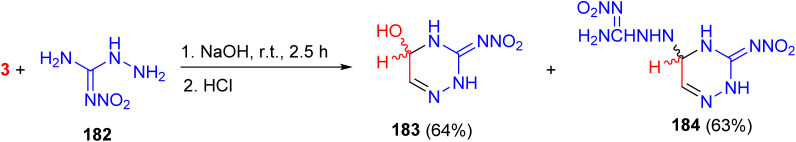
Synthesis of the triazine derivatives 183 and 184.

The reactions of 1-amino-2-nitroguanidine 182 with glyoxal in the presence of NaOH in water at room temperature for 2.5 h and then acidification with diluted HCl to pH 5, afforded 5-hydroxy-4,5-dihydro-3-nitroimino-1,2,4-triazine (183) in 64% yield. The same reaction conducted in a 1 : 2 ratio gave 5-aminonitroguanidyl-4,5-dihydro-3-nitroimino-1,2,4-triazine (184) in 63% yield (Scheme 73).^110^

**Scheme 74:**
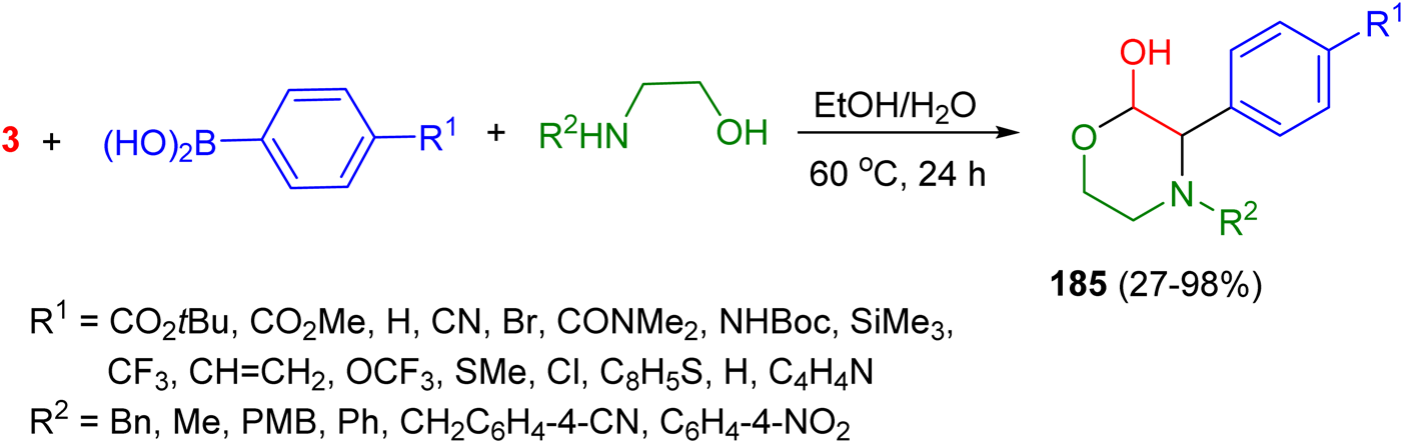
Synthesis of 2-hydroxy-3-arylmorpholines 185 using the Petasis borono-Mannich reaction.

Wright and his group prepared 2-hydroxy-3-arylmorpholines 185 in 27–98% yields from arylboronic acids, aqueous glyoxal, and 1,2-aminoethanols by a variant of the Petasis borono-Mannich reaction. The reaction proceeded in EtOH/H_2_O at 60 °C for 24 h. Upon treatment with methanesulfonic anhydride and triethylamine, these compounds underwent deoxygenation to generate intermediate 3,4-dihydro-2*H*-1,4-oxazines. Subsequent reduction with a triacetoxyborohydride salt and acetic acid yielded the 3-aryl morpholine products 185 (Scheme 74).^111^

**Scheme 75:**
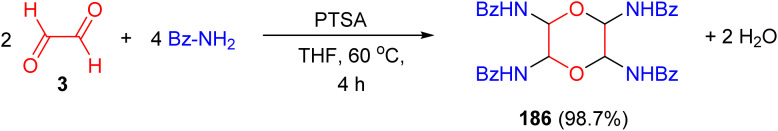
Synthesis of *N*,*N*′,*N*″,*N*‴-(1,4-dioxane-2,3,5,6-tetrayl)tetrabenzamide 186.

In 2022, Paromov *et al.* reported the synthesis of *N*,*N*′,*N*″,*N*‴-(1,4-dioxane-2,3,5,6-tetrayl)tetrabenzamide 186 in 98.7% yield by the condensation of benzamide with glyoxal in the presence of *p*TSA monohydrate in THF at 60 °C for 4 h (Scheme 75).^112^

### 4.3. Synthesis of polyaza polycyclic compounds

**Scheme 76:**
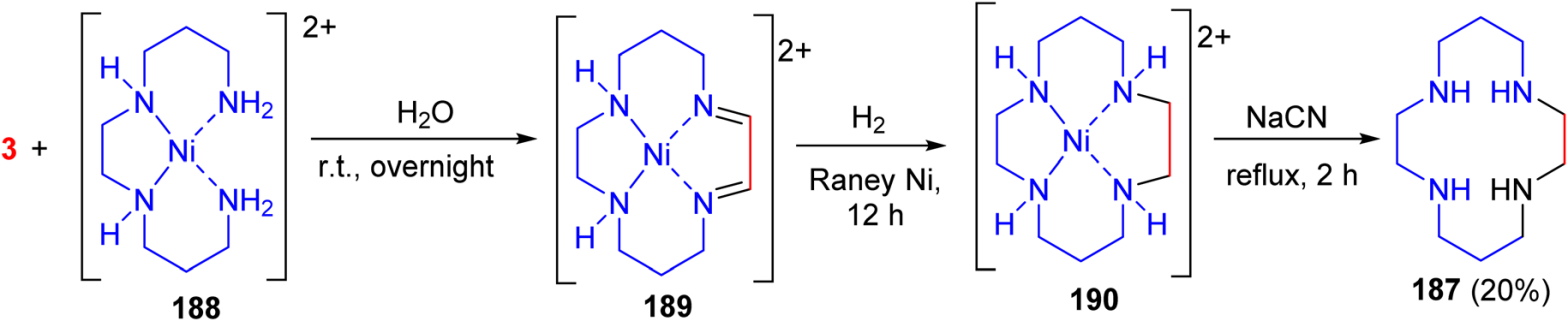
Synthesis of cyclam 187*via* the nickel(ii) complex.

In 1972, Barefield reported a three-step synthesis of 1,4,8,11-tetraazacyclotetradecane (cyclam, 187). The process began with the reaction of metal-ammine 188 and glyoxal in water at room temperature, which proceeded overnight to yield intermediate 189. In the second step, hydrogenation of 189 using RANEY® nickel over 12 h produced compound 190. Finally, refluxing a solution of 190 with NaCN for 2 h afforded cyclam (187) in 20% overall yield (Scheme 76).^113^

**Scheme 77:**
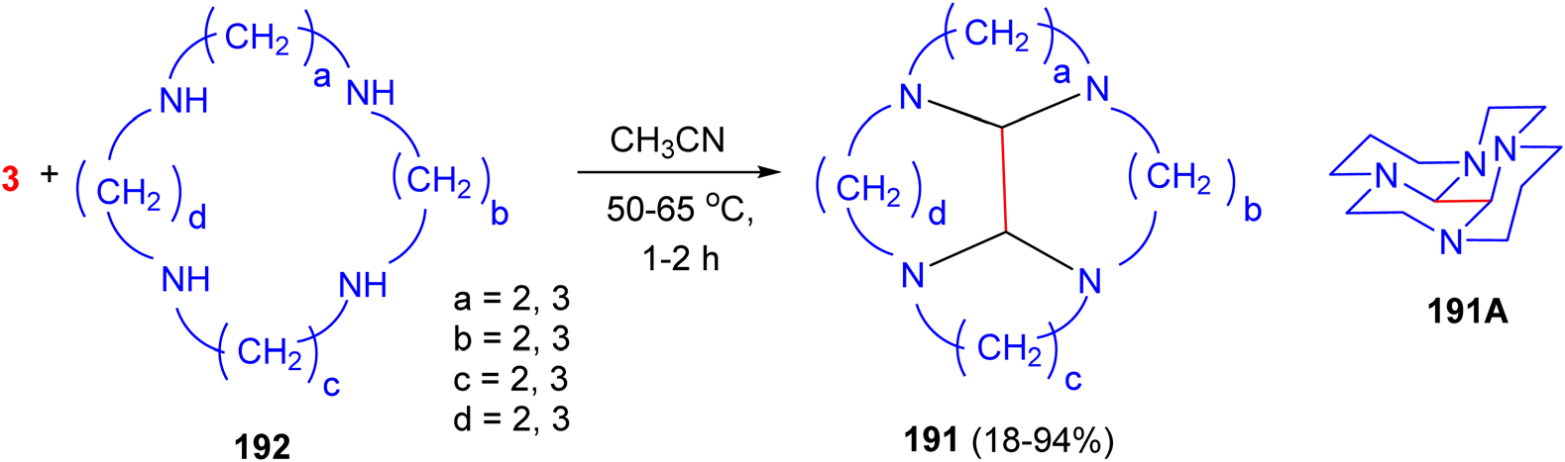
Preparation of tetracyclic tetramines 191.

In 1980, Weisman *et al.* obtained tetracyclic tetramines 191 in 18–94% yields by the condensation of tetramines 192 with glyoxal in CH_3_CN at 50–65 °C for 1–2 h under nitrogen. Dynamic ^13^C-NMR analysis showed the *cis*-stereochemistry of 191A (*a*, *b*, *c*, *d* = 2) and 191B (*a* = 3, *b* = 2, *c* = 3, *d* = 2) at room temperature (Scheme 77).^114^

**Scheme 78:**
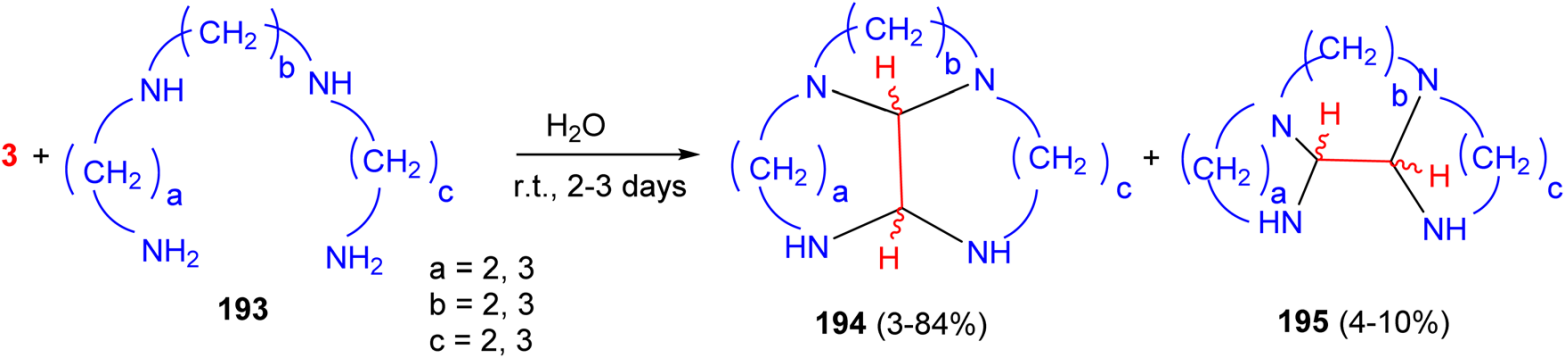
Synthesis of *cis*/*trans* tricyclic compounds 194 and 195.

Next, linear tetraamines 193 in the reaction with glyoxal in H_2_O at room temperature for 2–3 days afforded a mixture of *cis*/*trans*-tricyclic compounds 194 in 3–84% yields and 195 in 4–10% yields (Scheme 78).^115^

**Scheme 79:**
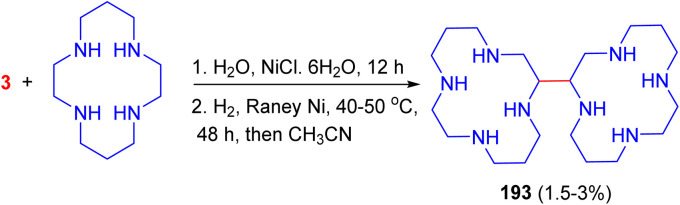
Synthesis of 2,2′-bi-(1,4,8,11-tetra-azacyclotetradecane) (193).

In 1981, Barefield and his group presented the synthesis of 2,2′-bis(1,4,8,11-tetra-azacyclotetradecane) (193) by the reaction of 1,5,8,12-tetra-azadodecane with 40% aqueous glyoxal in water, using NiCl·6H_2_O as a catalyst. The reaction was carried out for 12 h, followed by hydrogenation for 48 h at 40–50 °C in the presence of a RANEY^®^ nickel catalyst. Upon decomposition of the resulting macrocyclic nickel complexes with NaCN, compound 193 was isolated as a minor product (Scheme 79).^116^

**Scheme 80:**
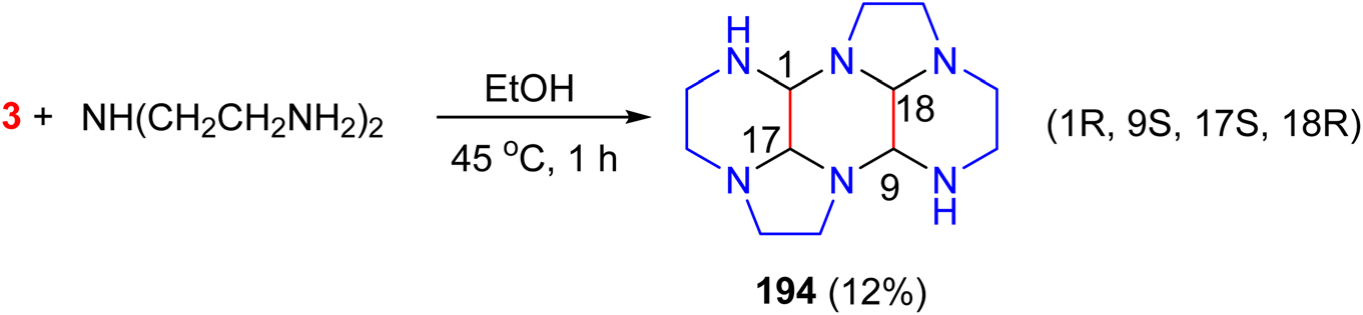
Preparation of hexaazapentacyclooctadecane 194.

Strasdeit and coworkers reported that reacting equimolar amounts of diethylenetriamine and aqueous glyoxal in ethanol at 45 °C for one hour produced the crystalline 2 : 2 adduct, 2,5,8,10,13,16-hexaazapentacyclo[8.6.1.1^2.5^.0^9,18^.0^13,17^]octadecane (194), with a yield of 12%. No other solid products, particularly no Schiff bases, were observed. Compound 194 is pentacyclic and has four asymmetric carbon atoms. Consequently, one of the two possible meso forms is present. The prefix *C*_i_ denotes the point symmetry. Moreover, the reaction of 194 with CdX_2_ (X = Cl, Br) in methanolic solutions at room temperature was studied (Scheme 80).^117^

**Scheme 81:**
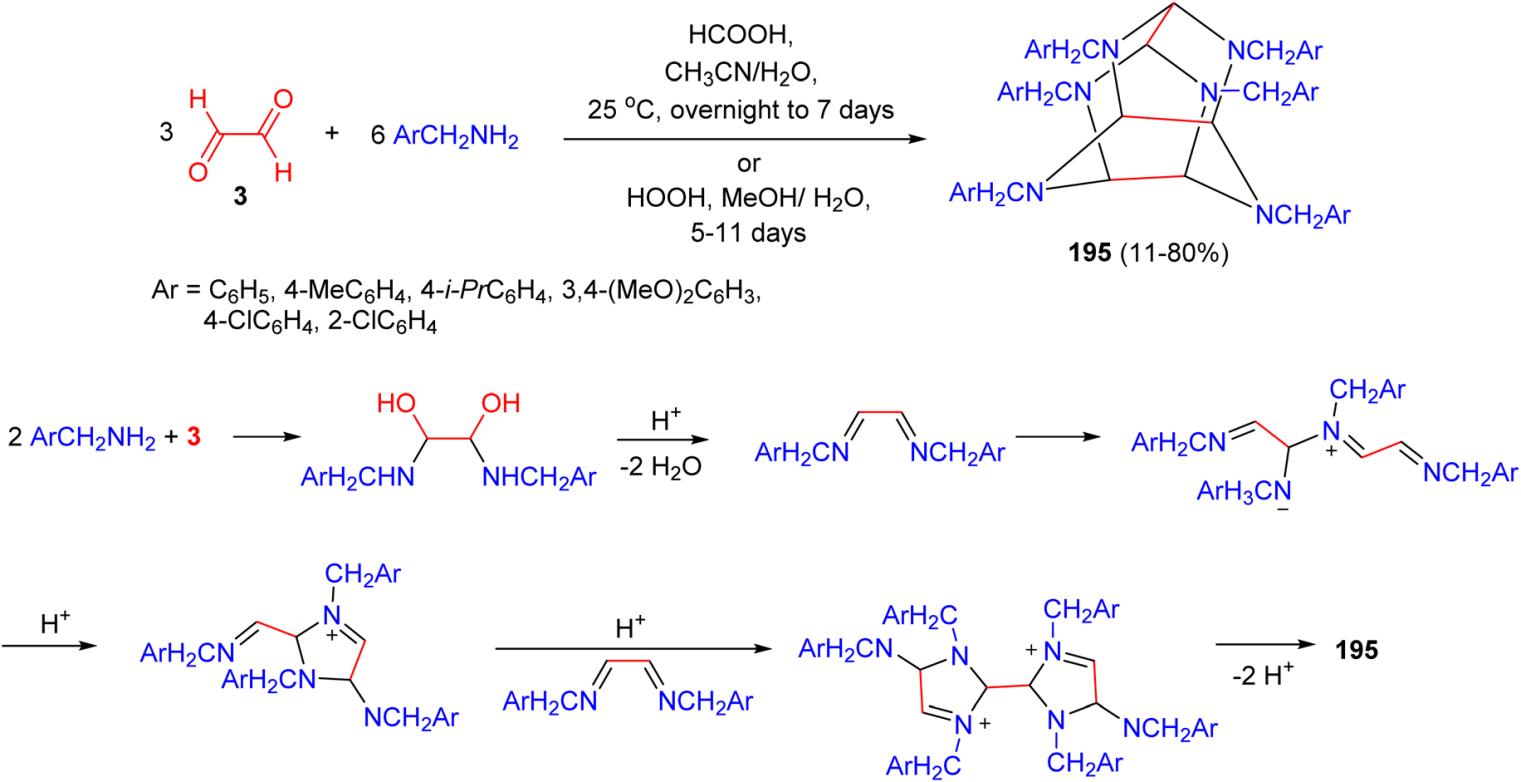
Synthesis of the tetraazatetracyclododecane derivatives 195.

In 1990, Nielsen and his colleagues developed two methods for synthesizing tetraazatetracyclododecane derivatives 195. This was achieved through the condensation of glyoxal with benzylamines in solvents such as acetonitrile/water with formic acid catalyst at 25 °C for overnight to 7 days with 24–80% yields, and in MeOH/H_2_O at 25 °C for 5–11 days with 11–64% yields. The proposed mechanism is depicted in Scheme 81.^118^

**Scheme 82:**
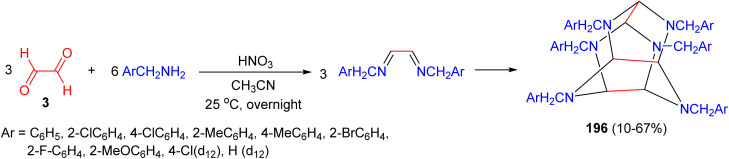
HNO_3_-catalyzed preparation of the tetraazatetracyclododecane derivatives 196.

In another study, the reaction of glyoxal with benzylamine or with substituted benzylamines in acetonitrile containing nitric acid overnight at room temperature led to the cage structure 196 in 10–67% yields. The diimines were implicated as reaction intermediates, as shown in Scheme 82.^119^

**Scheme 83:**
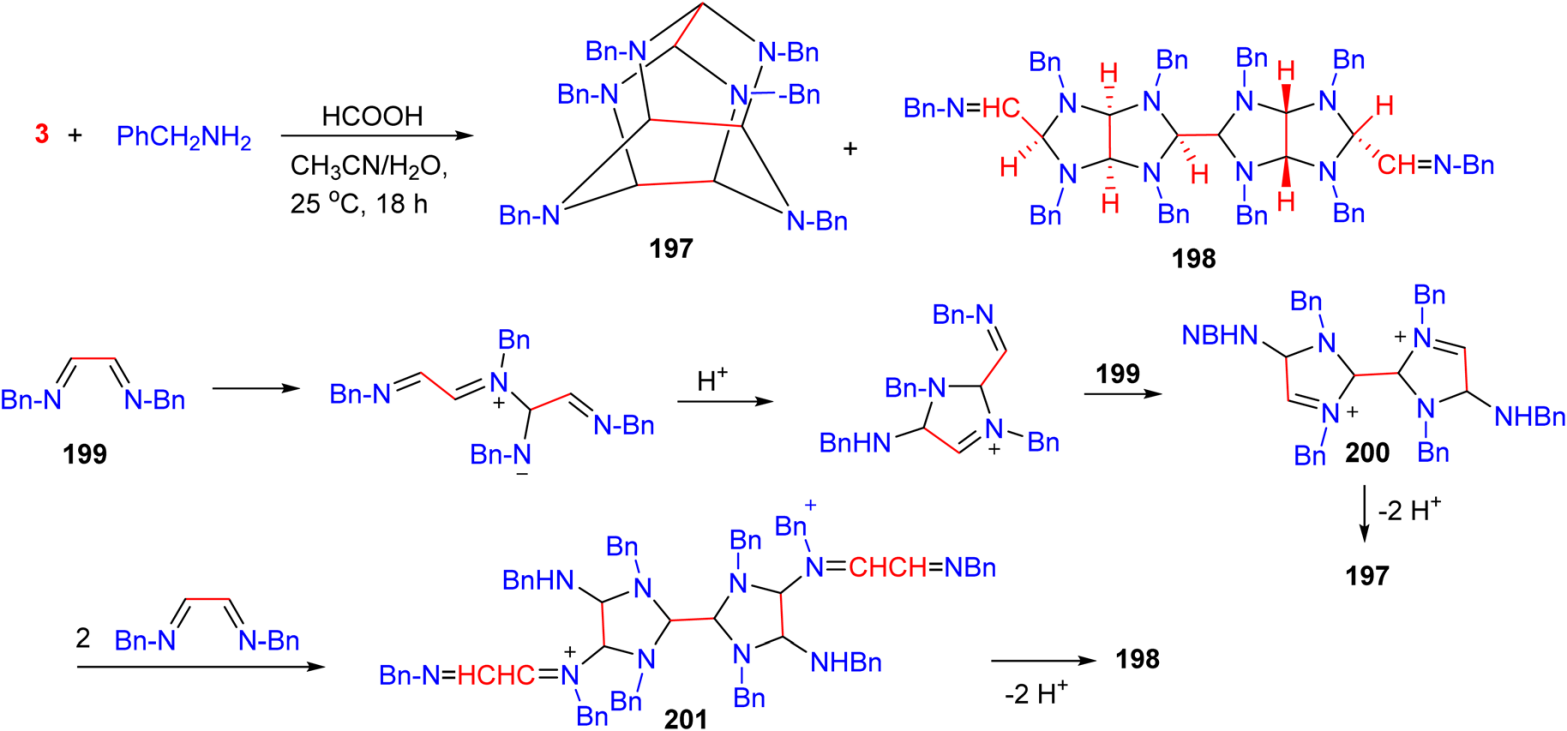
Preparation of polyazapolycyclic 197 and bi(2,4,6,8-tetraazabicyclo[3.3.0]octane) 198.

In 1992, Chaykovsky and his group reported that the condensation of glyoxal with benzylamine using formic acid as a catalyst in H_2_O/CH_3_CN at room temperature for 18 h afforded the polyazapolycyclic caged ring system 197 and bi(2,4,6,8-tetraazabicyclo[3.3.0]octane) 198. Recrystallization from acetonitrile (CH_3_CN) was used to separate these compounds. The mechanism of formation of 197 involves the trimerization, in discrete steps, of the conjugated dipolar diimine 199 (R = benzyl) to give, as an intermediate, the bicyclic dication 200. Intramolecular cyclization of 200 leads to the caged structure 197 after loss of two protons. However, if 200 were to react further with two more molecules of 199, one adding at each of the rings to give 201, then cyclization and loss of two protons leads to the bi(tetraazabicyc1ooctane) 198 (Scheme 83). X-ray crystallographic analysis was used to determine the crystal molecular structure of 198.^120^

**Scheme 84:**
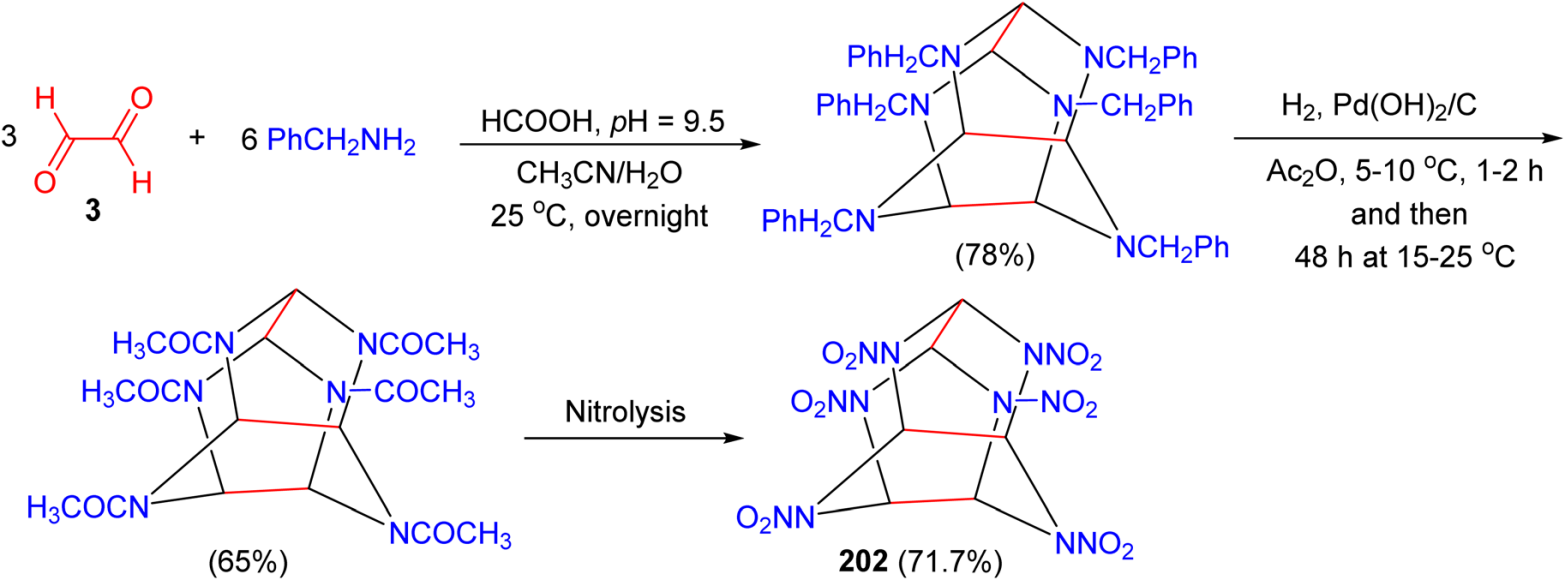
Formic acid catalyzed synthesis of hexanitrohexaazaisowurtzitane (HNIW) (202).

A polycyclic caged compound with high strain-hexanitrohexaazaisowurtzitane (HNIW) (202) was synthesized *via* a three-step reaction: condensation of benzylamine with glyoxal using formic acid a catalyst in CH_3_CN/H_2_O at 25 °C overnight, hydrogenolysis debenzylation in the presence of Pd(OH)_2_/C catalyst in Ac_2_O for 1–2 h at 5–10 °C and then for another 48 h at 15–25 °C, and a final nitrolysis. HNIW is the most powerful high-energy-density compound (HEDC) ever tested. HNIW's caged molecule is shaped approximately like a cube. In HNIW, each nitro group is planar, and the six groups are oriented in a crossed arrangement. This can result in tight packing and impart HNIW high density (Scheme 84).^121,122^

**Scheme 85:**
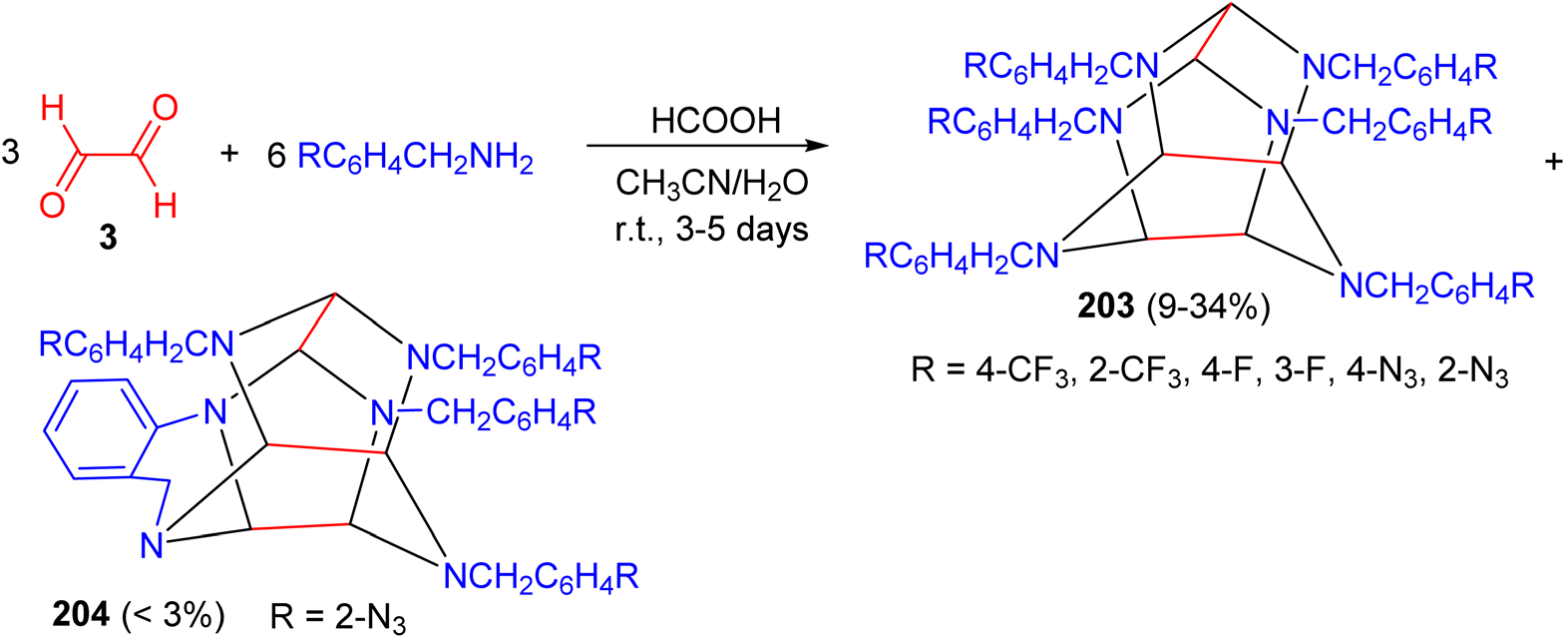
Synthesis of hexabenzylhexaazaisowurtzitanes 203 and 204.

Klapotke and his team reported the synthesis of hexaazaisowurtzitane-based polycycles 203 in 9–34% yields by reacting benzylamines with glyoxal in the presence of formic acid as a catalyst in a CH_3_CN/H_2_O (10 : 1) mixture at room temperature over 3–5 days. Moreover, with azide in the 2-position, 204 was isolated and identified as a type of polycycle (Scheme 85).^123^

**Scheme 86:**
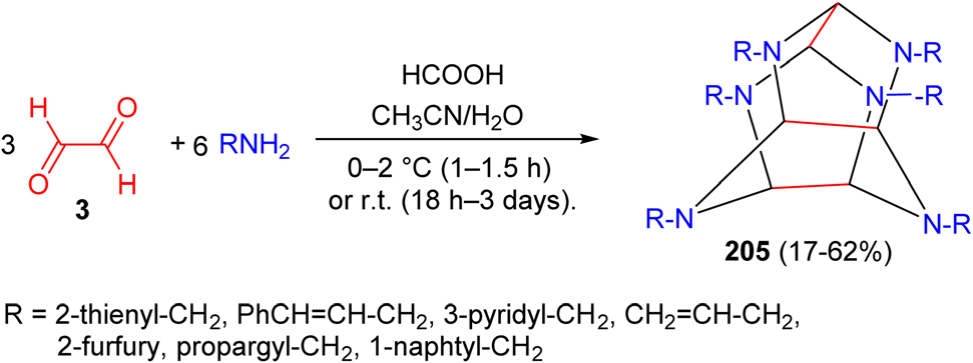
Synthesis of the hexaazaisowurtzitane cages 205.

In 2006, Herve *et al.* described the synthesis of hexaazaisowurtzitane cages (205) in 17–62% yields *via* the condensation of amines with glyoxal, catalyzed by HCOOH in CH_3_CN/H_2_O. The reaction proceeded at 0–2 °C (1–1.5 h) or at ambient temperature (18 h – 3 days). Steric effects hindered the formation of cage structures with α-substituted benzyl- and allylamines, though these substrates efficiently yielded diimines. Molecular mechanics calculations provided a rational explanation for this steric limitation (Scheme 86).^124^

**Scheme 87:**
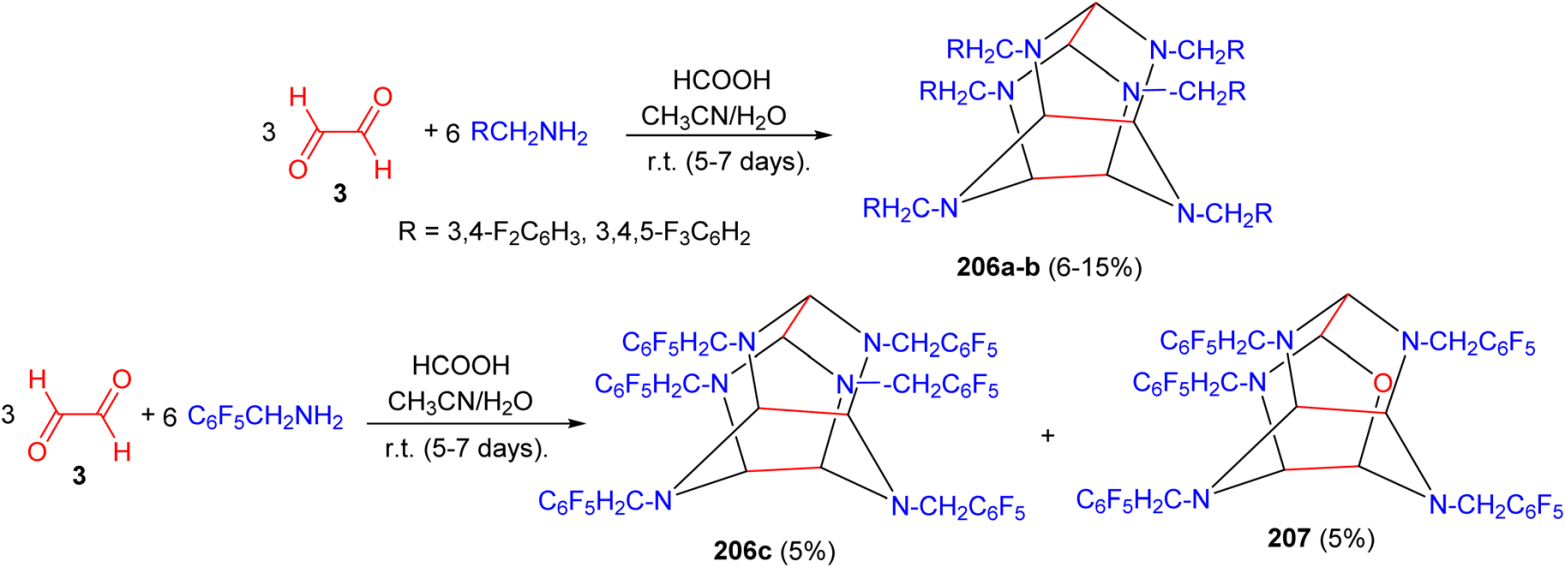
Synthesized polyfluorinated isowurtzitanes 206 and 207.

After that, Klapotke *et al.* synthesized polyfluorinated hexabenzylhexaazaisowurtzitanes 206a–c in modest yields (5–15%) *via* the condensation of polyfluorobenzylamines with glyoxal in a CH_3_CN/H_2_O solvent system, catalyzed by formic acid at ambient temperature over 5–7 days. Notably, the reaction produced an unusual isowurtzitane by-product 207, which was isolated in 5% yield. The study revealed an inverse relationship between fluorine content and isolated yields, with higher fluorination leading to lower yields and increased challenges in purification (Scheme 87).^125^

**Scheme 88:**
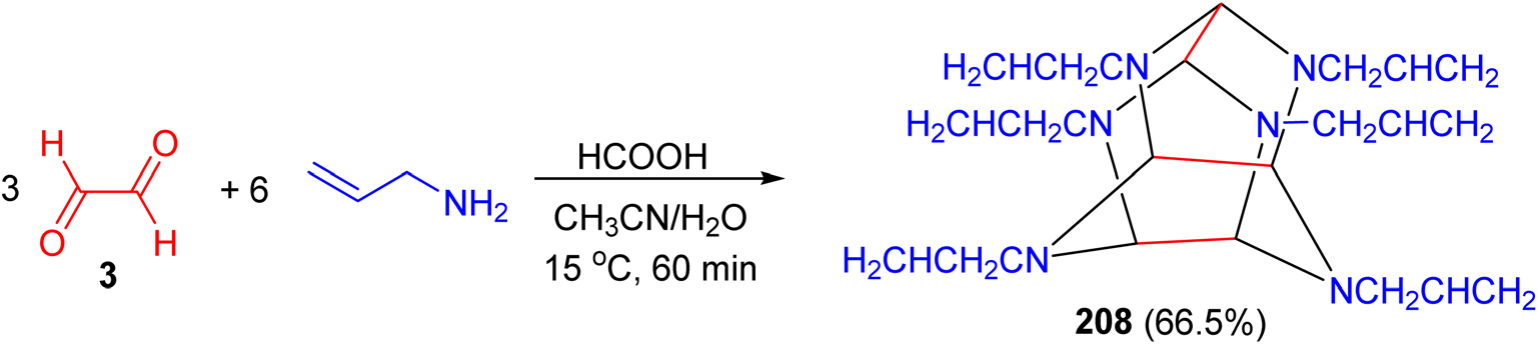
Preparation of 2,4,6,8,10,12-hexaallyl-2,4,6,8,10,12-hexaazaisowurtzitane (HALLIW) 208.

In 2009, 2,4,6,8,10,12-hexaallyl-2,4,6,8,10,12-hexaazaisowurtzitane (HALLIW) 208 was synthesized in 66.5% yield in a condensation reaction of glyoxal with allylamine in the presence of a protonic acid as a catalyst at 15 °C for 60 min. Optimization of the synthesis was accomplished by means of a mathematical experiment using planning theory with the steepest descent method. A method was developed for the purification of the crude product (Scheme 88).^126^

**Scheme 89:**
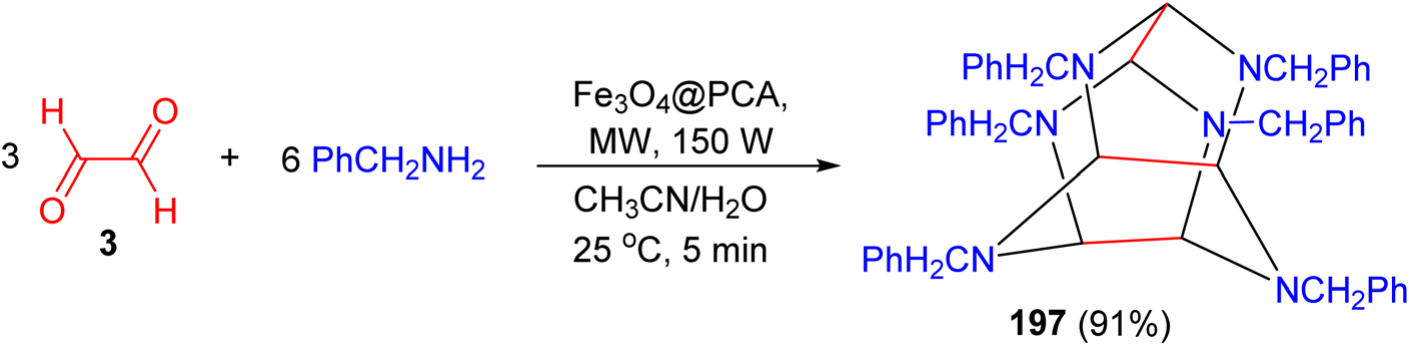
Preparation of HBIW (197) catalyzed by Fe_3_O_4_@PCT.

Joo and his group utilized Fe_3_O_4_@polycitric acid (PCA) nanoparticles as a heterogeneous solid acid catalyst for the synthesis of hexabenzylhexaazaisowurtzitane (HBIW) (197, 91% yield) from benzylamine and glyoxal in acetonitrile–water solvent under ultrasonic irradiation conditions at room temperature for 5 min. The catalyst could be reused up to 6 times without significant loss of activity (Scheme 89).^127^

**Scheme 90:**
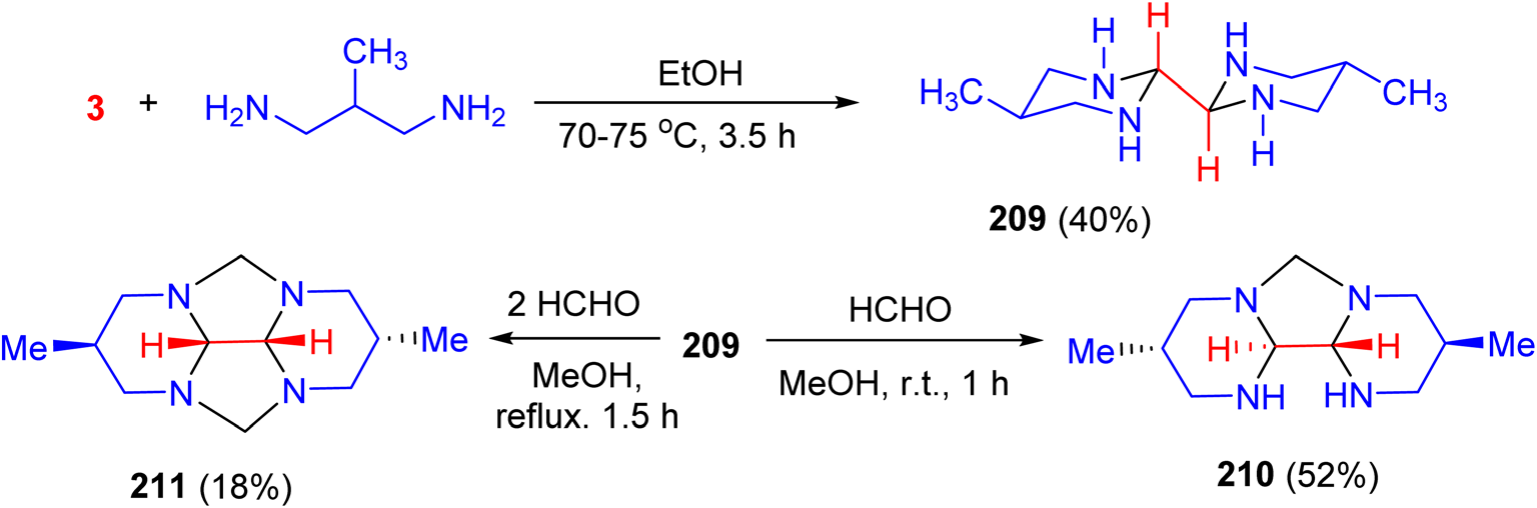
Synthesis of heterocyclic compounds 209 and 211.

Read *et al.* reported the synthesis of 2,5-*trans*-2′,5′-dimethylperhydro-2,2′-bipyrimidine (209) in 40% yield through the reaction of 2-methylpropane-1,3-diamine with glyoxal in EtOH at 70–75 °C for 3.5 h. Treatment of 209 with an equimolar amount of HCHO in MeOH at room temperature for 1 h readily gave tricycle 210 in 52% yield, after distillation. Treatment of 209 with 2 equiv. of HCHO in refluxing MeOH for 1.5 h gave 211 as a major product in 18% yield (Scheme 90).^128^

**Scheme 91:**
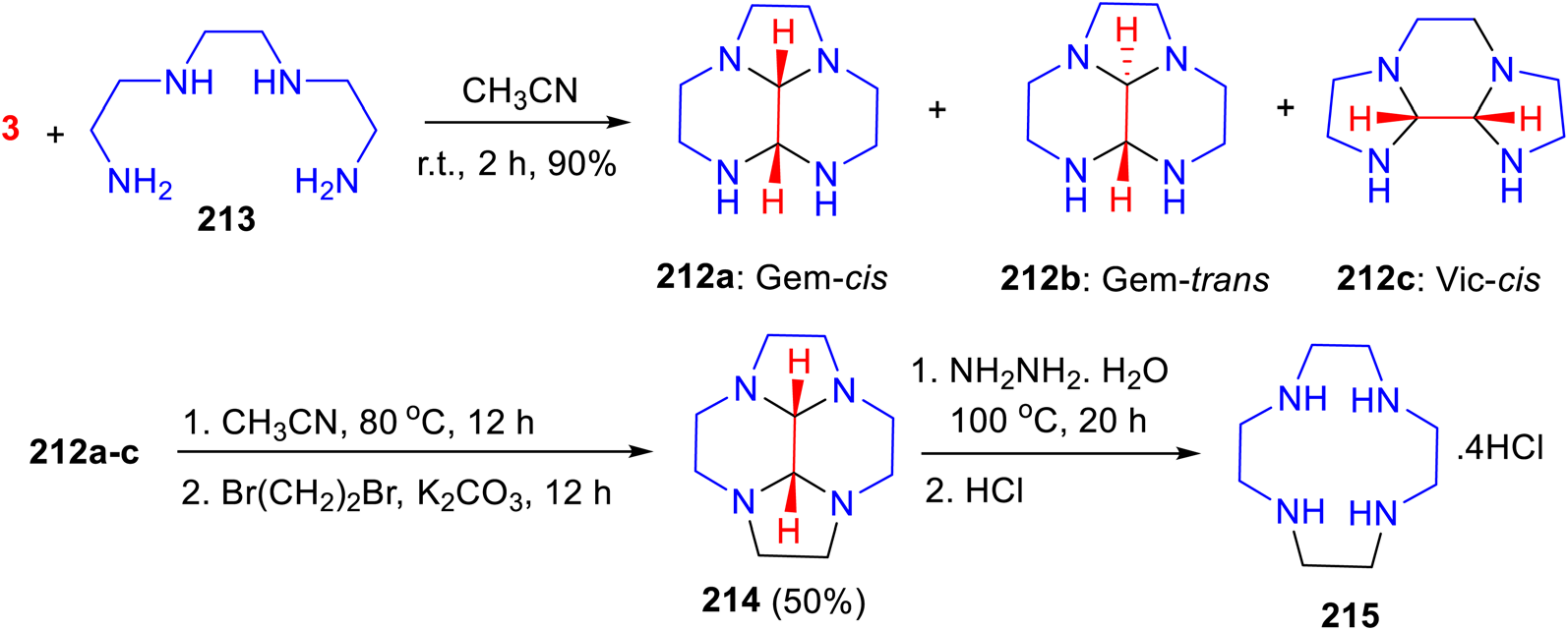
Synthesis of bis-aminal isomers 212a–c, tetracyclic tetraamine 214 and cyclen 215.

In 1999, Herve and co-workers synthesized a mixture of bis-aminal isomers 212a–d in 90% yield by using triethylenetetramine (213) and glyoxal in acetonitrile at room temperature for 2 h. Cyclization of this isomer mixture with dibromoethane in acetonitrile at 80 °C for 12 h, then 12 h in the presence of K_2_CO_3_, led to the formation of tetracyclic tetraamine 214 in 50% yield. The cyclen 215 was achieved by the reaction of 214 with an excess of hydrazine monohydrate for 20 h at 100 °C (Scheme 91).^129^

**Scheme 92:**
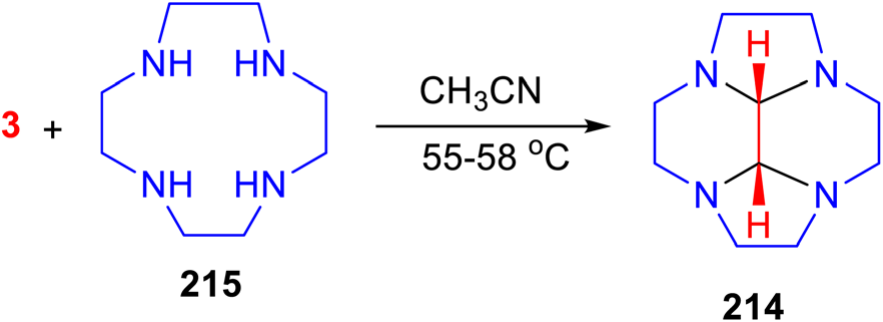
Synthesis of polycyclic compound 214.

After that, Faruk Khan *et al.* reported the synthesis of 214 by the reaction of cyclen 215 with glyoxal in dry CH_3_CN at 55–58 °C. Then, compound 215 was used for the synthesis of tetraazamacrocyclic bisquinoline derivatives as potential antimalarial agents (Scheme 92).^130^

**Scheme 93:**
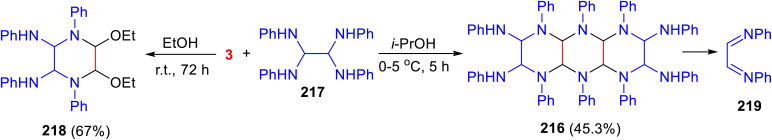
Synthesis of the polyazapolycyclic compound 216 and pyrazine derivative 218.

In 2005, Kakanejadifard and his group reported the synthesis of polyazapolycyclic compound 216 in 45.3% yield through the reaction of 1,1′,2,2′-tetrakis(phenylamino)ethane (217) with glyoxal in *i*-PrOH at 0–5 °C for 5 h. Moreover, compound 218 was obtained in 67% yield *via* the reaction of 217 with glyoxal in EtOH at room temperature for 72 h. It should be noted that compound 218 can be obtained by recrystallization of compound 216 in EtOH. Compound 218 is stable at room temperature, while compound 216 is degraded to 219 over 3 days (Scheme 93).^131^

**Scheme 94:**
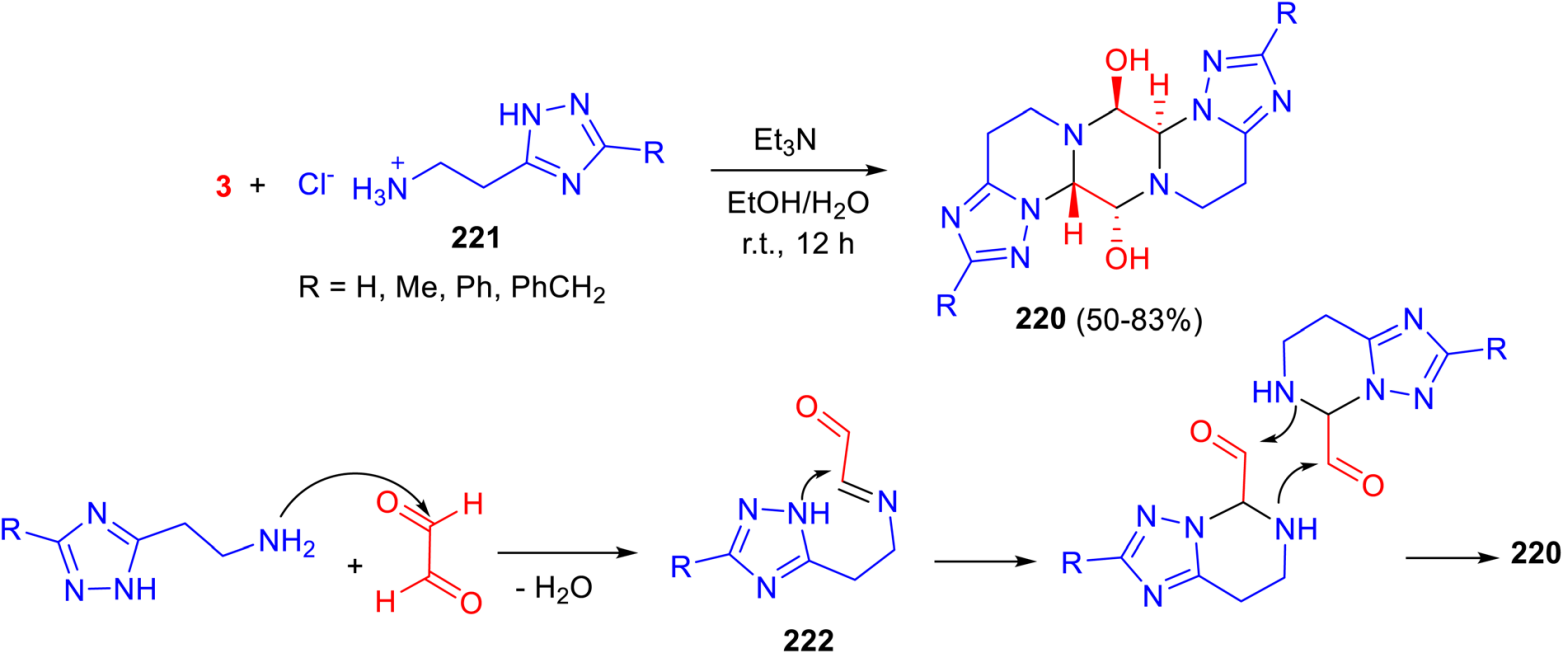
Preparation of pentacyclic heterocycles 220.

Khomenko and his team reported the synthesis of pentacyclic heterocycles 220 in 50–83% yields by the reaction of aminoethyl-1,2,4-triazole salts 221 and glyoxal in the presence of NEt_3_ in EtOH/H_2_O at room temperature for 12 h. The formation of 220 is explained in Scheme 94. The condensation of aminotriazole with one carbonyl group of glyoxal gives azomethine intermediate 222, which undergoes cyclization through intramolecular attack of the NH group of the 1,2,4-triazole to the C=N moiety, furnishing the tetrahydro[1,2,4]triazolo[1,5-*c*]pyrimidine-5-carbaldehyde derivative 223. Subsequent double hemiaminal formation *via* stepwise intermolecular-intramolecular addition processes between the amino and formyl groups of two molecules of 223 provides the product 220.^132^

**Scheme 95:**
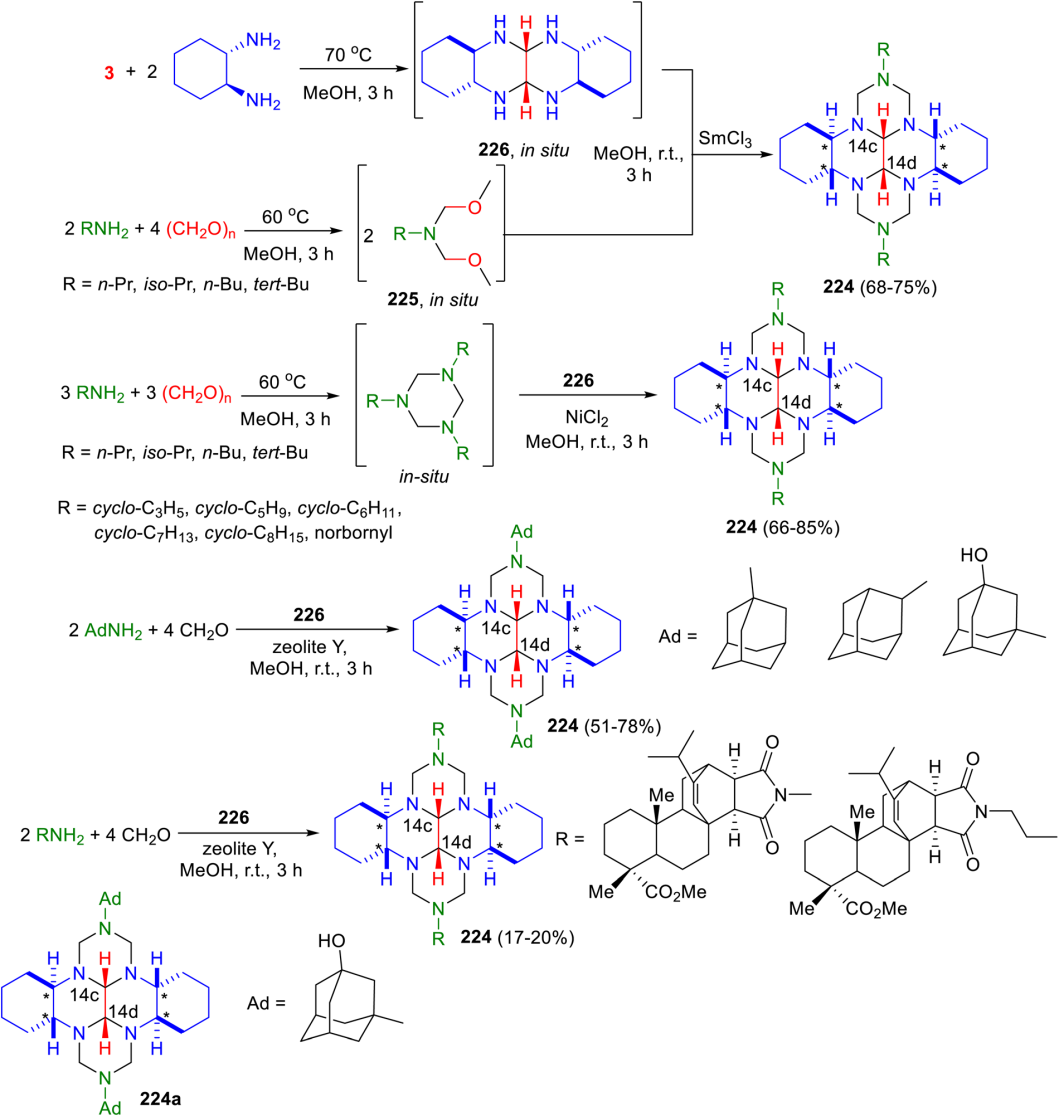
Synthesis of perhydrohexaazadibenzotetracenes 224.

In 2020, Dzhemileva and co-workers reported the synthesis of 2,9-disubstituted 3bR*,7aR*,10bR*,14aR*-*cis*-14*c*,14*d*-perhydro-2,3*a*,7*b*,9,10*a*,14*b*-hexaazadibenzotetracenes 224 in 17–85% yields through intermolecular heterocyclization of *N*,*N*-bis(methoxymethyl)-*N*-alkylamines 225 with *trans*-1,6,7,12-tetraazaperhydrotetracene 226 in the presence of SmCl_3_·6H_2_O, NiCl_2_·6H_2_O or zeolite Y in MeOH at room temperature for 3 h. The synthesized perhydro hexazadibenzotetracenes possess an R*, R*, R*, R*-relative configuration at the C3b, C7a, C10b, and C14a stereocenters, as well as a *cis*-junction of the rings at the C14c-C14d bond. Structural elucidation was achieved through 1D (^1^H, ^13^C) and 2D (COSY, HSQC, HMBC) NMR spectroscopy, MALDI-TOF/TOF mass spectrometry, and X-ray crystallography (Scheme 95). A cytotoxic effect of the perhydrohexaazadibenzotetracenes synthesized thereby was determined based on the IC_50_ for six tumor cell cultures (Jurkat, K562, U937, A549, A2780 and T74D) and the same for normal fibroblasts (Fibroblasts). Compound 224a was demonstrated to possess a cytostatic activity (a proliferation-restrictive activity) with regard to cells in all studied lines.^133^

**Scheme 96:**
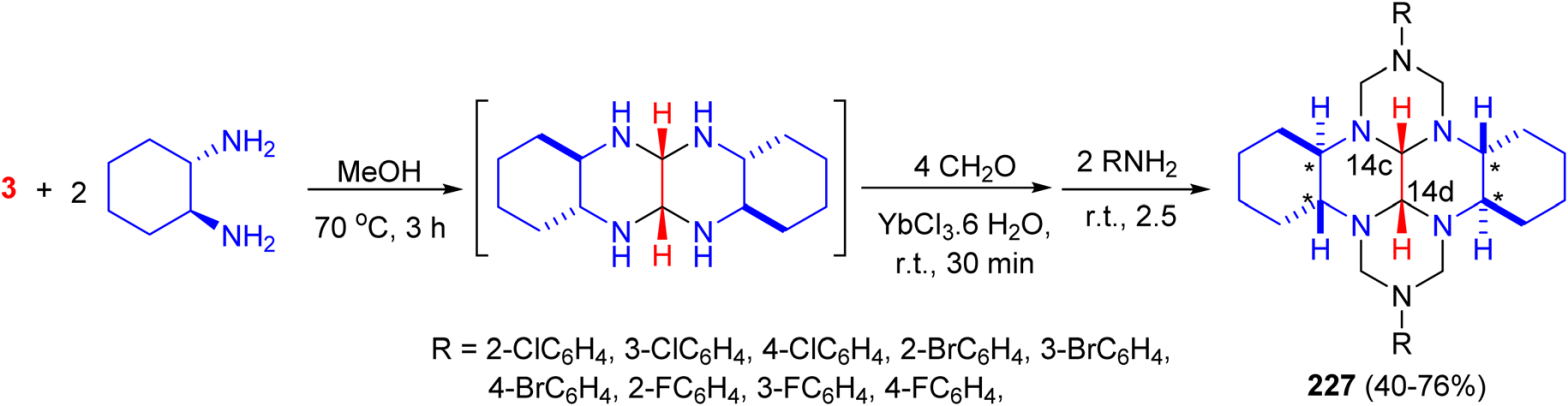
Synthesis of 2,9-bis(halophenyl)-substituted perhydrohexaazadibenzotetracenes 227.

A one-pot procedure was developed for the synthesis of 2,9-bis(halophenyl)-substituted perhydrohexaazadibenzotetracenes 227 in 40–76% yields. The method entails first condensing (±)-*trans*-cyclohexane-1,2-diamine with glyoxal in MeOH at 70 °C for 3 h, followed by the addition of formaldehyde, a haloaniline, and YbCl_3_·6H_2_O as a catalyst, with the reaction then proceeding at room temperature for 3 h. A plausible mechanism of the formation of 227 involves the intermediate formation of the tetrakis(hydroxymethyl) derivative from *trans*-1,6,7,12-tetraazaperhydrotetracene and formaldehyde. Presumably, coordination of YbCl_3_·6H_2_O as a hard Lewis acid to the hydroxy oxygen atom of the intermediate product generates the carbocation, and the subsequent nucleophilic addition of the haloaniline to that carbocation yields the target fused polyazapolycyclic compounds (Scheme 96).^134^

**Scheme 97:**
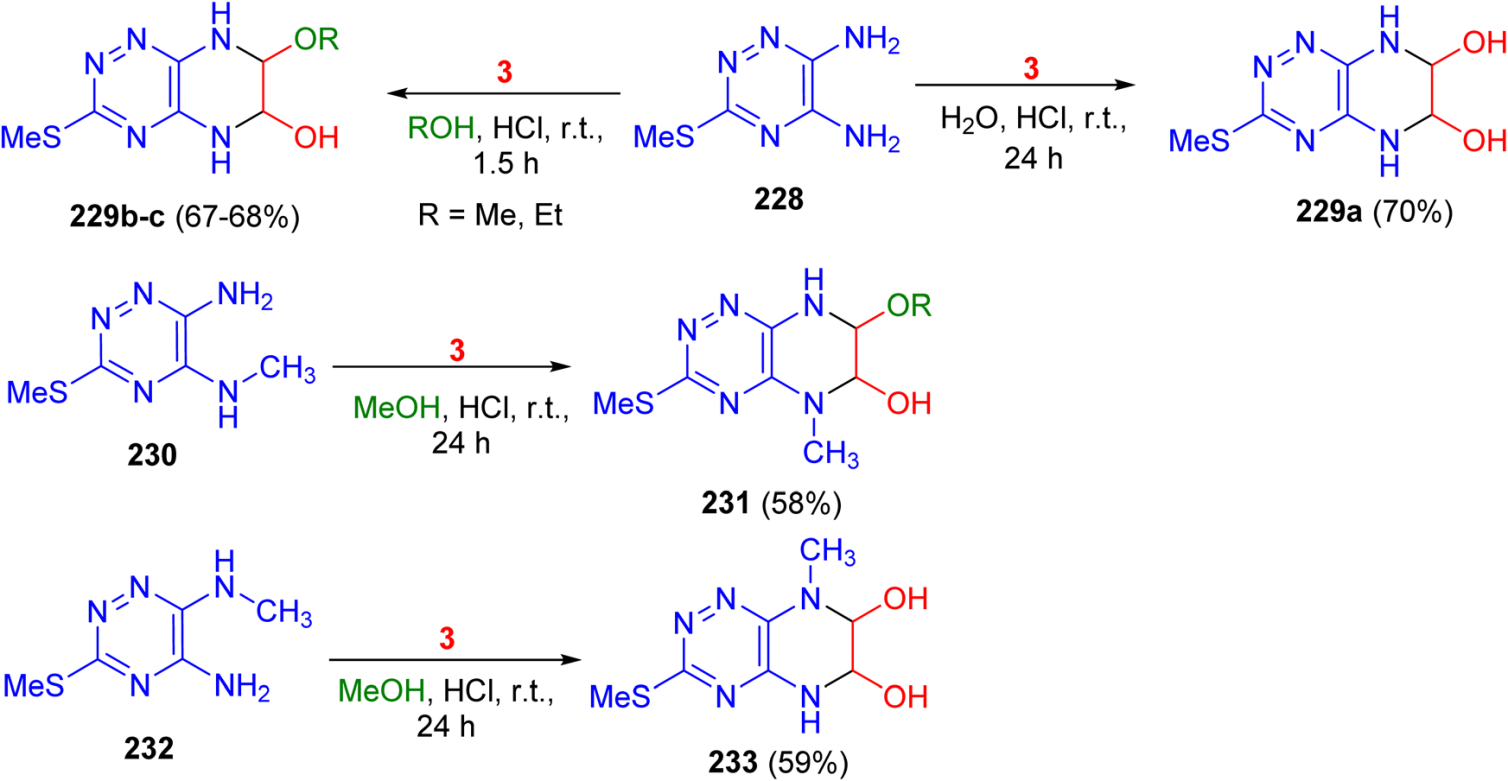
Synthesis of the pyrazino triazine ring systems 229, 231 and 233.

In 1983, Panzica *et al.* investigated the reaction of 5,6-diamino-3-methylthio-*as*-tiazine (228) with 40% aqueous glyoxal under neutral or acidic conditions at room temperature for 24 h, which gave 6,7-dihydroxy-3-methylthio-5,6,7,8-tetrahydropyrazino[2,3-*e*]-*as*-triazine (229a). When adduct 228 was dissolved in alcohol and stirred at room temperature for 1.5 h, the corresponding methoxy 229b or ethoxy 229c adducts were obtained, depending on the alcohol used. Additionally, cyclization of 230 with glyoxal in methanol produced the 7-methoxy adduct 231, while compound 232 yielded only the dihydrate 233 under the same conditions (Scheme 97).^135^

**Scheme 98:**
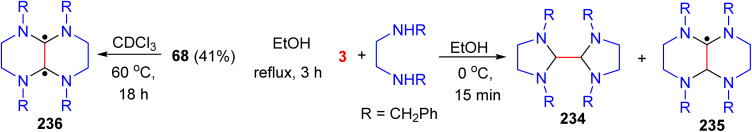
Synthesis of biimidazolidines 234 and tetraazadecalins 235, 236.

Willer and his group discovered that the reaction of *N*,*N*′-dibenzylethylenediamine with glyoxal in ethanol at 0 °C for 15 min afforded 1,1′,3,3′-tetrabenzyl-2,2′-biimidazolidine (234) and *trans*-1,4,5,8-tetrabenzyl-l,4,5,8-tetraazadecalin (235) in a 60 : 40 ratio as the initial products. Additionally, compound 235 was found to undergo reversible isomerization to the *cis*-isomer 236 in CDCl_3_, with a determined 
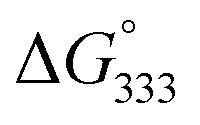
 of <0.1 kcal mol^−1^. However, dynamic behavior was observed in the ^1^H and ^13^C NMR spectra of 235 and 236, resulting from nitrogen inversion and ring-flip processes, respectively (Scheme 98).^136^

**Scheme 99:**
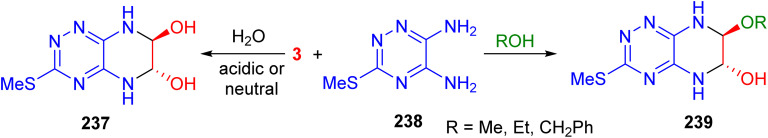
Synthesis of the pyrazino[2,3-*e*]-*as*-triazine ring systems 237 and 239.

Hodgson and colleagues prepared 6,7-dihydroxy-5,6,7,8-tetrahydropyrazino[2,3-*e*]-*as*-triazines 237, by ring closure of selected 5,6-diamino-*as*-triazines 238 with 40% aqueous glyoxal. These 4-azapteridines experience a novel exchange process with alcohols at the C(7)-position. When dissolved in alcohol and stirred at room temperature, the 7-alkoxy, 6-hydroxy analogues 239 are formed and isolated. In fact, during ring closure, if alcohols are used as the solvent, only the latter compounds are obtained. A single-crystal X-ray diffraction study determined the predominant and most stable adduct to be the *trans* (*R*,*R* or *S*,*S*) isomer (Scheme 99).^137^

**Scheme 100:**
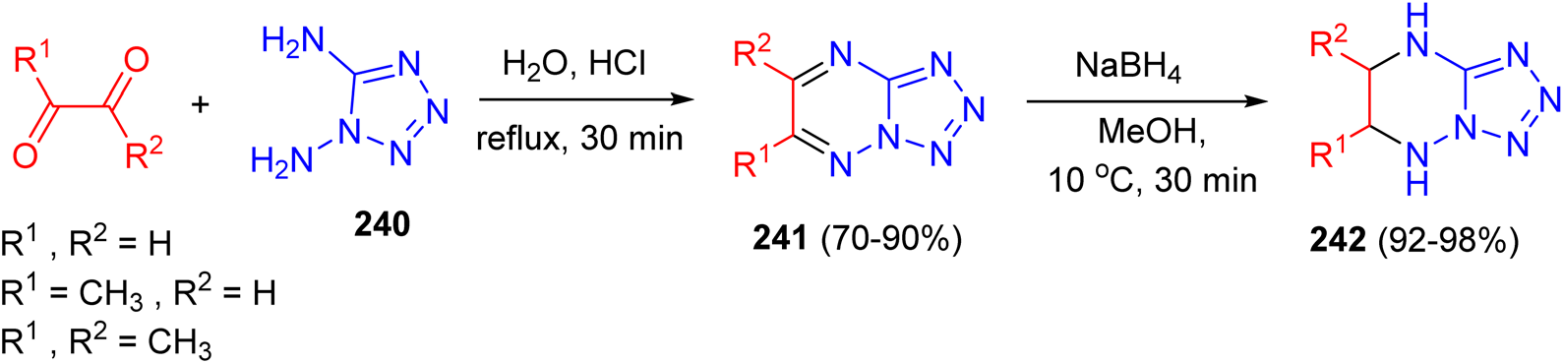
Synthesis of tetrazolo[1,5,*b*][1,2,4]triazines 241 and 5,6,7,8-tetrahydrotetrazolo[1,5-*b*][1,2,4]triazines 242.

In another study, the reaction of 1,5-diaminotetrazole (240) with 1 equiv. of glyoxal, pyruvic aldehyde, or biacetyl in water under reflux conditions for 30 min with an acid catalyst gave the desired tetrazolo[ 1,5,*b*][1,2,4]-triazines 241 in 70–90% yields, contaminated in the glyoxal and pyruvic aldehyde cases with a small amount of a highly insoluble byproduct. Compounds 241 are rapidly reduced with sodium borohydride in methanol at 10 °C for 30 min to give high yields of the 5,6,7,8-tetrahydro compounds 242 in 92–98% yields (Scheme 100).^138^

**Scheme 101:**
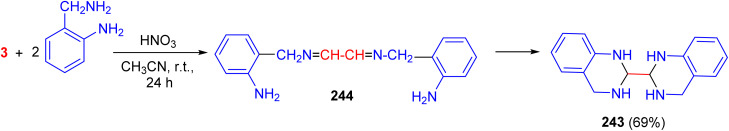
Synthesis of 2.2′-bi(1,2,3,4-tetrahydroquinazoline) (243).

Batsanov *et al.* studied the condensation of glyoxal with 2-aminobenzylamine in acetonitrile containing 70% aqueous nitric acid at room temperature for 24 h, which gave 2,2′-bi(1,2,3,4-tetrahydroquinazoline) (243) in 69% yield. This is presumably formed by intramolecular cyclisation of 244. The structure of 243 was confirmed by X-ray crystallography (Scheme 101).^139^

**Scheme 102:**
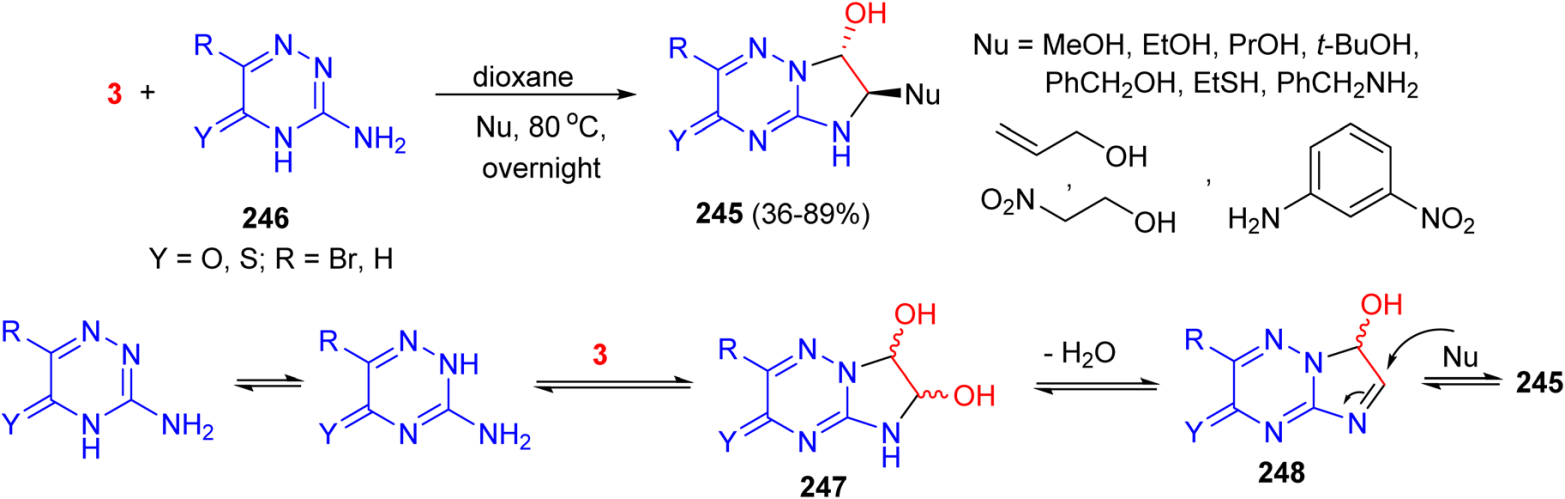
Diastereoselective synthesis of C(6)-functionalized dihydroimidazotriazines 245.

In 2003, Guillaumet *et al.* described the regio- and diastereoselective synthesis of C(6)-functionalized dihydroimidazotriazines 245 in 36–89% yields by ring closure of different 5(2*H*)-1,2,4-triazin-3-ones 246 with aqueous glyoxal and various nucleophiles (alcohols, thiols, or amines) in dioxane at 80 °C overnight. The suggested mechanism is demonstrated in Scheme 102. First, a double addition of amino groups on each aldehyde moiety of glyoxal occurs to form the diol intermediate 247. This diol would be in equilibrium with imine 248, onto which a nucleophile could add. The structure and exact stereochemistry were established through a combination of single-crystal X-ray diffraction analysis and ^1^H and ^13^C NMR spectroscopy.^140^

**Scheme 103:**
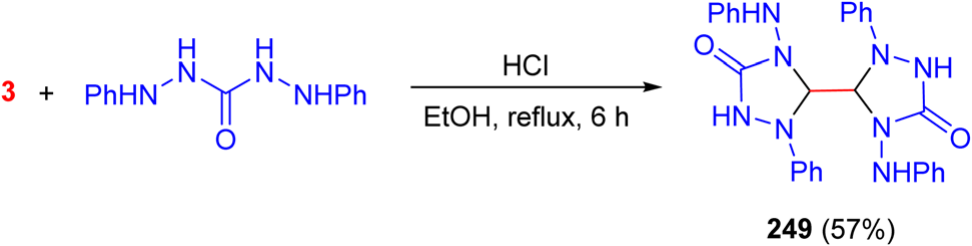
Acid catalyzed synthesis of bicyclohexaaza compound 249.

In 2016, the reaction of 1,5-diphenylcarbazide with aqueous glyoxal in ethanol at reflux, catalyzed by hydrochloric acid over 6 h, yielded a white precipitate with 57% yield. Spectroscopic analysis, supported by computational studies, revealed only one C=O stretching vibration band in the experimental IR spectrum, confirming that the obtained product corresponds to 249 (Scheme 103).^141^

**Scheme 104:**
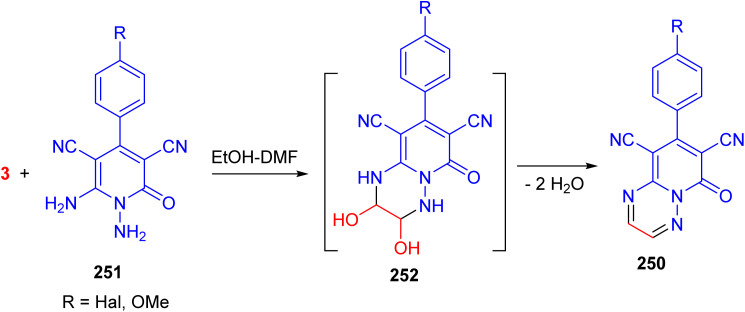
Preparation of 8-aryl-6-oxo-6*H*-pyrido[1,2-*b*][1,2,4]triazine-7,9-dicarbonitriles 250.

In 2023, Dotsenko and his group developed a method to produce 8-aryl-6-oxo-6*H*-pyrido[1,2-b][1,2,4]triazine-7,9-dicarbonitriles 250 by the reaction of pyridines 251 with glyoxal in EtOH/DMF under mild conditions. Obviously, the reaction proceeds through the formation of the corresponding semi-aminals 252 with subsequent dehydration (Scheme 104).^142^

### 4.4. Synthesis of polyoxa polycyclic compounds

**Scheme 105:**
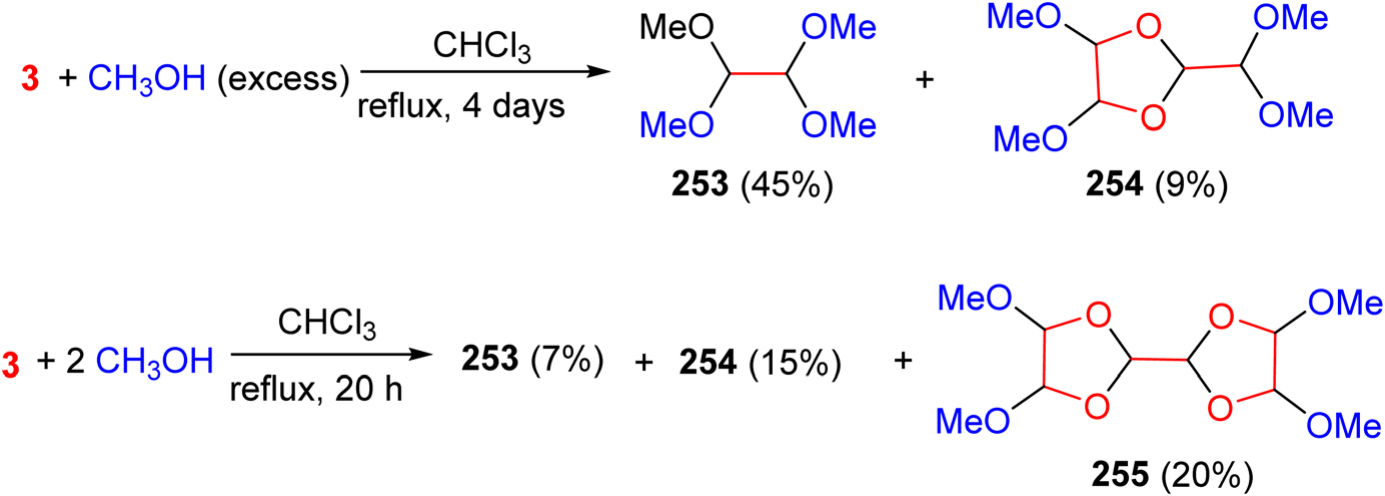
Synthesis of the 1,3-dioxalane and bis-1,3-dioxalane derivatives 254, 255.

In 1972, the Kliegman group found that reacting 80% aqueous glyoxal with excess methanol under acidic conditions in refluxing chloroform for four days produced 1,1,2,2-tetrakis(methoxy)ethane (253) in 45% yield and 2-dimethoxymethyl-4,5-dimethoxy-1,3-dioxolane (254) in 9% yield. However, when the reaction was carried out with only 2 equivalents of methanol per glyoxal under reflux in chloroform for 20 hours, the product distribution shifted toward higher molecular weight species. In this case, the dimer 254 and a trimer (255) became the major products, obtained in 15% and 20% yields, respectively (Scheme 105).^143^

**Scheme 106:**
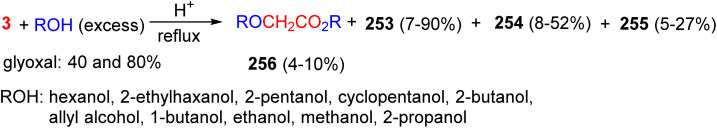
Products 253–256 isolated from the alcohol-glyoxal reactions.

In 1973, the Kliegman group reported the reaction of aqueous glyoxal with alcohols in the presence of *p*-toluenesulfonic acid under reflux conditions. Water was removed azeotropically by the refluxing alcohol using a continuous Dean–Stark apparatus, yielding glycolates and acetal products. These products included glycolate 256, 1,1′,2,2′-tetraalkoxyethanes 253, 1,3-dioxolanes 254, and 1,3-bisdioxolanes 255. The relative abundance of any of the acetal products depends on the initial glyoxal concentration as well as the initial ratio of alcohol to glyoxal in the reaction mixture. It is also shown that dioxolane formation can be rationalized not only by the reaction of alcohol with dimeric and trimeric glyoxal, but also *via* the direct reaction of glyoxal with any of the already formed acetals (Scheme 106).^144^

**Scheme 107:**
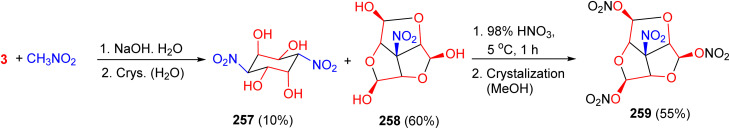
Synthesis of the trinitrate derivative 259.

The condensation of nitromethane with glyoxal in an aqueous NaOH solution at room temperature for 2 h yielded two products: 3,6-dinitro-cyclohexane-1,2,4,5-tetraol (257, 10%) and, unexpectedly, the tricyclic nitro-triol 258 (60%). The latter was converted into 6b-nitrohexahydro-2*H*-1,3,5-trioxacyclopenta[*cd*]pentalene-2,4,6-triyl trinitrate (259) in 55% yield by treatment with 98% HNO_3_ at 5 °C for 1 h. Compound 259 was further characterized for explosive properties, including impact and friction sensitivity, activation energy, detonation velocity, heat of combustion (under oxygen), and enthalpy of formation (Scheme 107).^145^

### 4.5. Synthesis of polyaza–polyoxa polycyclic compounds

**Scheme 108:**
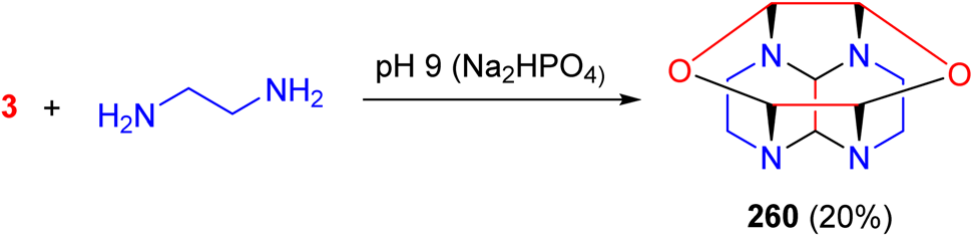
Synthesis of a heterocyclic cage compound 260.

In 1968, the Edwards group reported the synthesis of a heterocyclic cage compound 260 from ethylenediamine and glyoxal in a molar ratio of 2 : 3, in dilute aqueous solution buffered to pH 9 (Na_2_HPO_4_) (Scheme 108). Compound 260 is easily isolated by extraction into chloroform and purified by crystallization from ethanol or by vacuum sublimation. Its structure was confirmed by X-ray crystallographic analysis.^146^

**Scheme 109:**
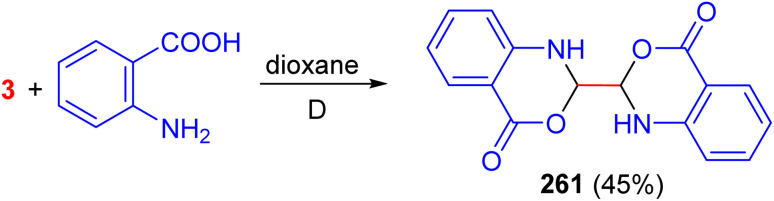
Synthesis of 2,2′-bis(l,2-dihydro-4-oxo-3,1-benzoxazine) (261).

In 1970, the Kliegman group reported the synthesis of 2,2′-bis(l,2-dihydro-4-oxo-3,1-benzoxazine) (261) in 45% yield by the reaction of glyoxal with *o*-aminobenzoic acid in hot dioxane (Scheme 109).^147^

**Scheme 110:**
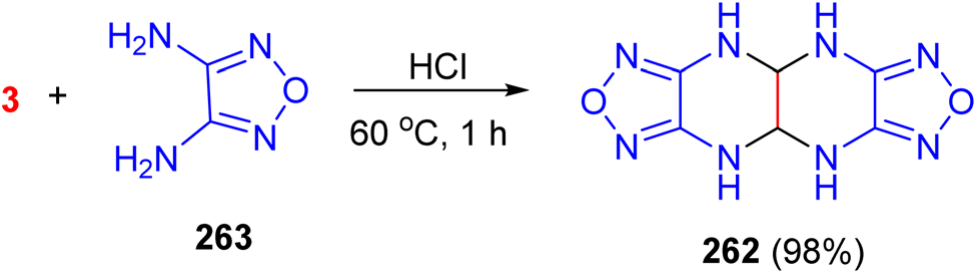
Synthesis of tetracyclic compound 262.

Next, Willer obtained compound 262 in 98% yield from the reaction of 3,4-diaminofurazan (263) with glyoxal in warm HCl solution for 1 h. This compound was fully characterized spectroscopically. However, they were unable to establish the stereochemistry of the ring junction (Scheme 110).^148^

**Scheme 111:**
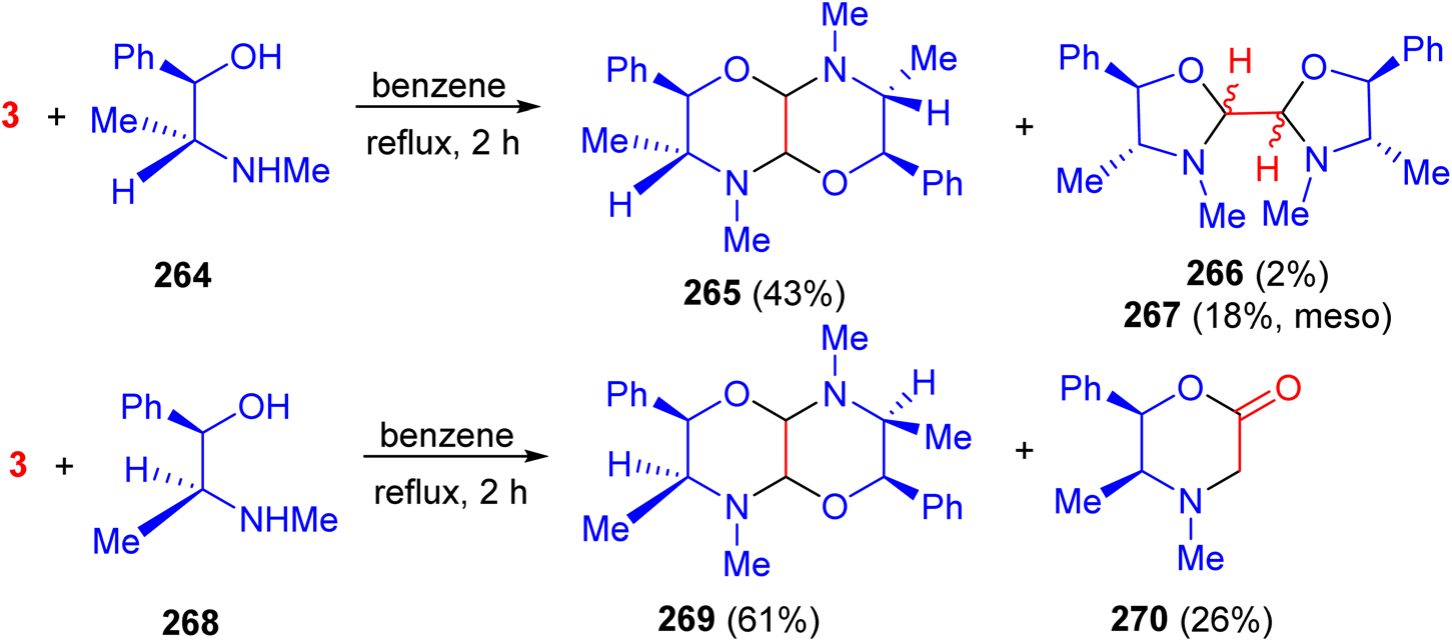
Synthesis of heterocyclic compounds 265–267, 269 and 270.

In another study, Farfan *et al.* found that reacting glyoxal with (1*R*,[2*R*)-(-)-pseudoephedrine (264) in benzene under reflux for 2 h using a Dean–Stark apparatus yielded a mixture of heterocyclic compounds 265–267 in 2–43% yields. These products were separated through multiple recrystallizations using methanol-hexane mixtures. Similarly, when (1*R*,2*S*)-(-)-ephedrine (268) was condensed with glyoxal under identical conditions, a mixture of 269 and 270 was obtained in 61 and 26% yields, respectively; the mixture was then separated using the same purification method (Scheme 111).^149^

**Scheme 112:**
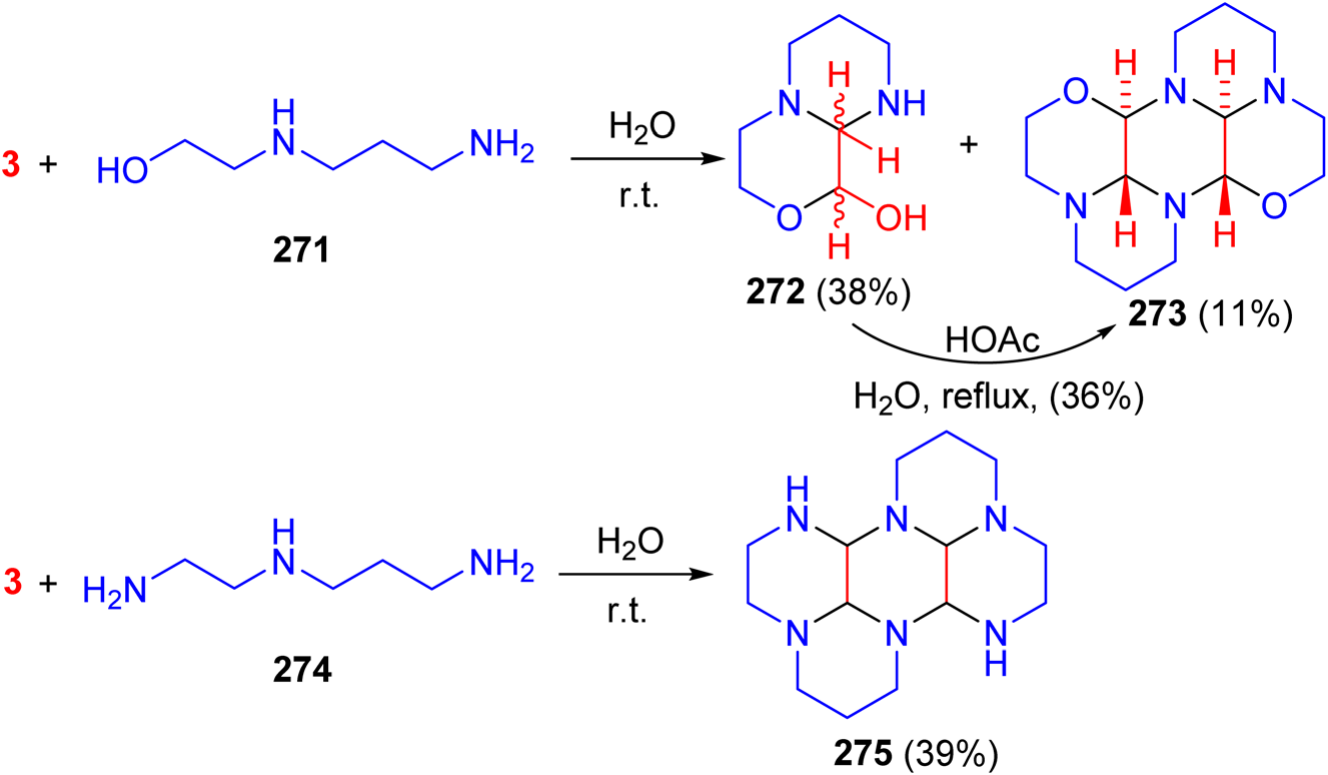
Synthesis of perhydroprimidinomorpholine 272 and pentaheterocycles 273, 275.

In 1993, Okawara and coworkers found that the one-pot reaction of glyoxal with 2-(3-aminopropylamino)ethanol (271) in water at room temperature gave the perhydroprimidinomorpholine 272 and 273 in 38 and 11% yields, respectively. Compound 272 was converted into 273 in 36% yield by refluxing an aqueous solution in the presence of AcOH as a catalyst. Similarly, the reaction of *N*-(2-aminoethyl)-1,3-propanediamine (274) with glyoxal gave 275 in 39% yield (Scheme 112).^150^

**Scheme 113:**
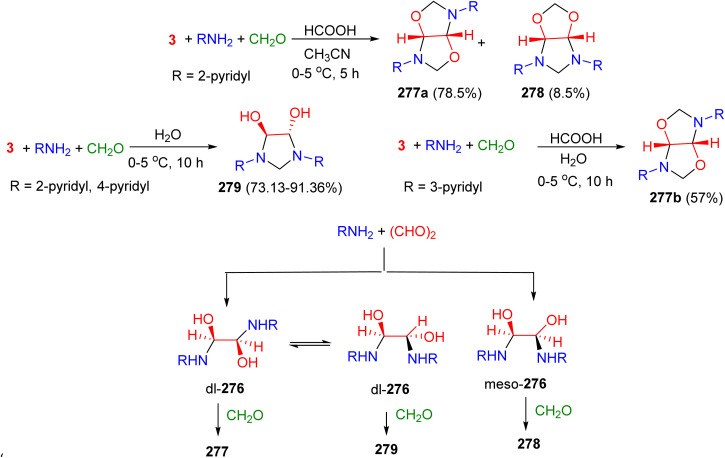
Synthesis of bicyclooctanes 277 and 278 and imidazolidines 279.

In 1997, Farnia and colleagues reported that the condensation of 2-aminopyridine with glyoxal proceeds with high selectivity to give a mixture of meso (minor) and dl (major) diol 276 as intermediates that could be easily transformed into the corresponding bicyclooctanes 277 and 278 with formaldehyde and acetonitrile as the solvent. In water, however, the reaction selectively produces imidazolidines 279. Based on NMR analysis, the major diastereomers were assigned as the *syn*-derivatives. A network of hydrogen bonding between the pyridyl nitrogens and the hydroxy hydrogens forms a pattern of twelve atoms in a chair-like conformation (Scheme 113). Differing products formed in the reactions in acetonitrile and water from 276 due to the modes of trapping of the reversibly formed intermediate, the conformational equilibria of which are likely to be solvent dependent.^151^

**Scheme 114:**
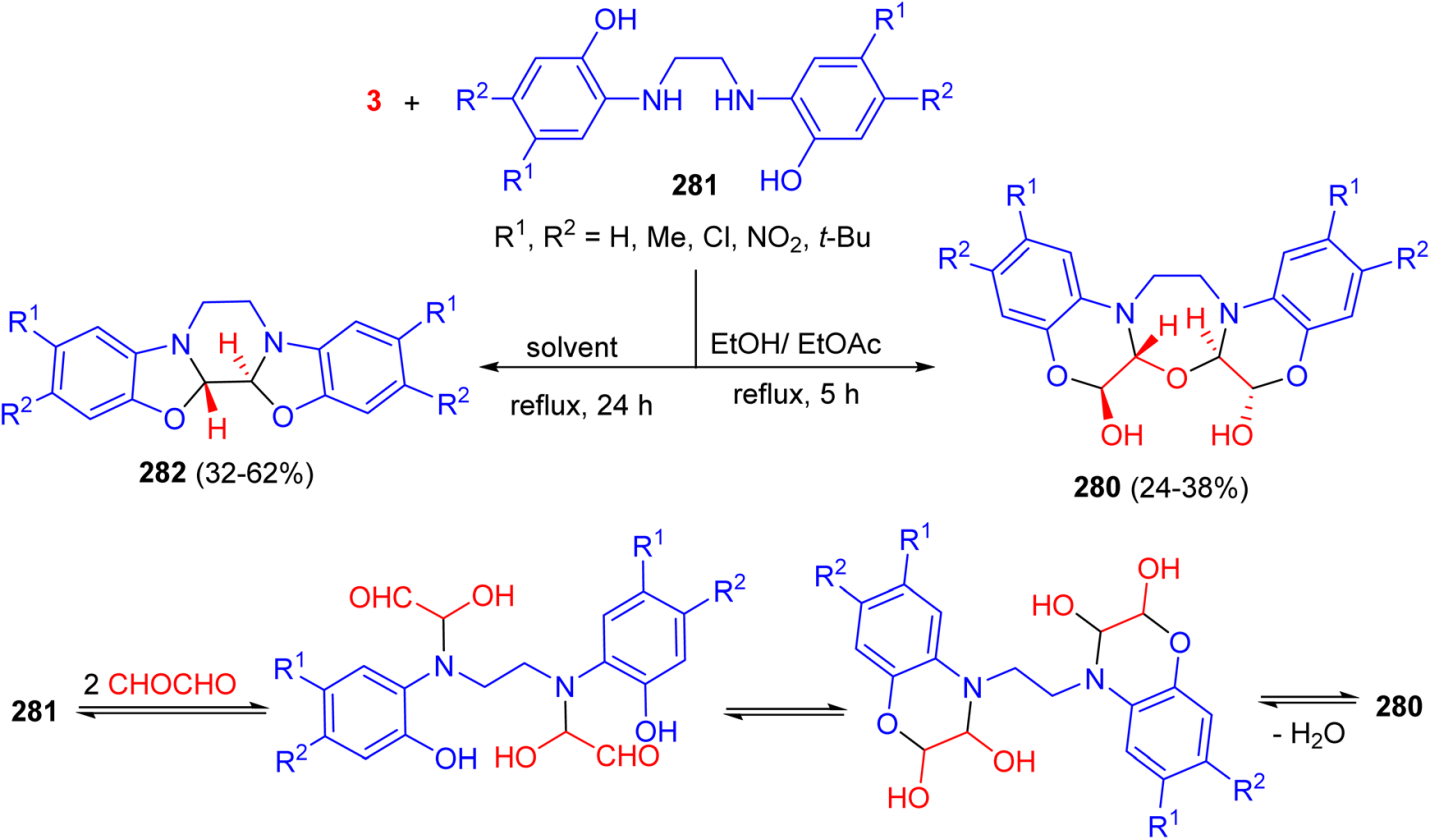
Synthesis of 1,3,6-oxadiazepines 280 and *N*,*N*′-ethylene-2,2′-bisbenzoxazolidines 282.

In 2001, facial synthesis of 1,3,6-oxadiazepine derivatives 280 was reported in 24–38% yields by the reaction of 2,2′-(1,2-ethanediyldiimino)bisphenol (281) with 2 equiv. of glyoxal in EtOH–ethyl acetate under reflux conditions for 5 h. Moreover, the reaction of 281 with 1 equiv. of glyoxal in appropriate solvents under reflux for 24 h using a Dean–Stark trap resulted *N*,*N*′-ethylene-2,2′-bisbenzoxazolidines 282 in 32–62% yields. The proposed mechanism for the synthesis of 280 is outlined in Scheme 114.^152^

**Scheme 115:**
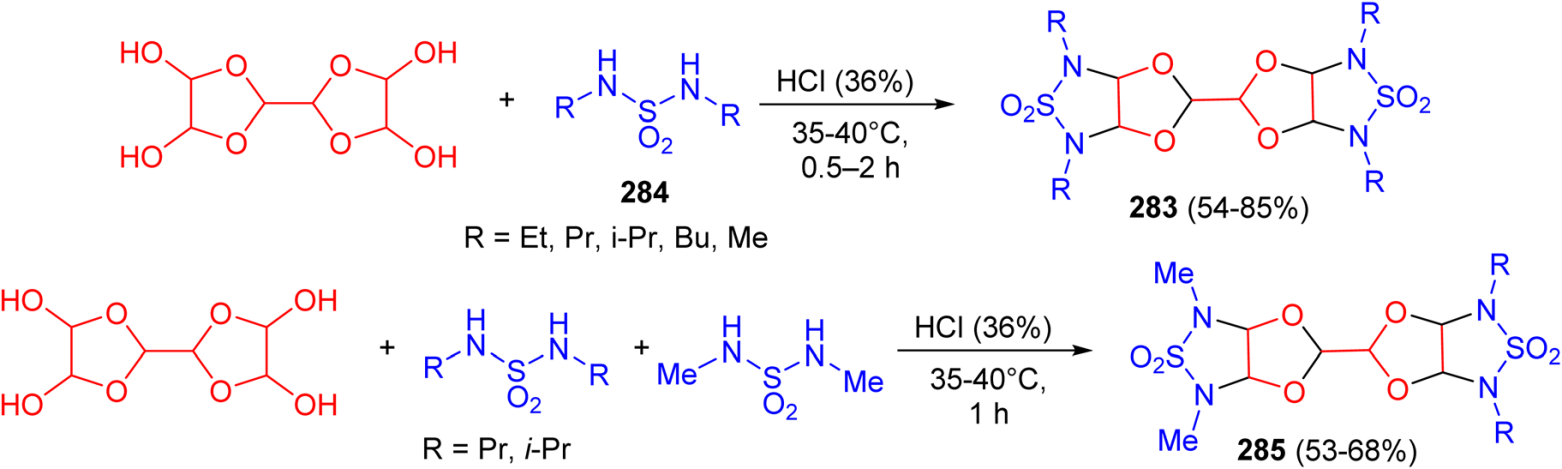
Synthesis of polyaza–polyoxa compounds 283 and 285.

Gazieva and his team developed a method to synthesize 3,3′-bi{(1R*,3 s*,5S*)-6,8-dialkyl-2,4-dioxa-7-thia-6,8-diazabicyclo[3.3.0]octane-7,7-dioxides} 283 in yields of 54–85%. The reaction involves 1,3-dialkylsulfamide 284 and glyoxal dihydrate trimer in the presence of concentrated HCl at 35–40 °C for 0.5–2 h. Additionally, compounds 285 were synthesized in 53–68% yields by reacting 1,3-dimethylsulfamide, glyoxal dihydrate trimer, and either 1,3-dipropyl- or 1,3-diisopropylsulfamide using concentrated HCl (∼36%) at 35–40 °C for 1 h. Compound 283a (R = *i*-Pr) showed weak bacteriostatic activity against *Staphylococcus aureus*, with an effective concentration >1000 µg mL^−1^. Meanwhile, compound 283b (R = Me) demonstrated fungicidal effects against pathogens causing root rot and seed mold in crops, although its activity was at least two times weaker than that of thiram (Scheme 115).^153^

**Scheme 116:**
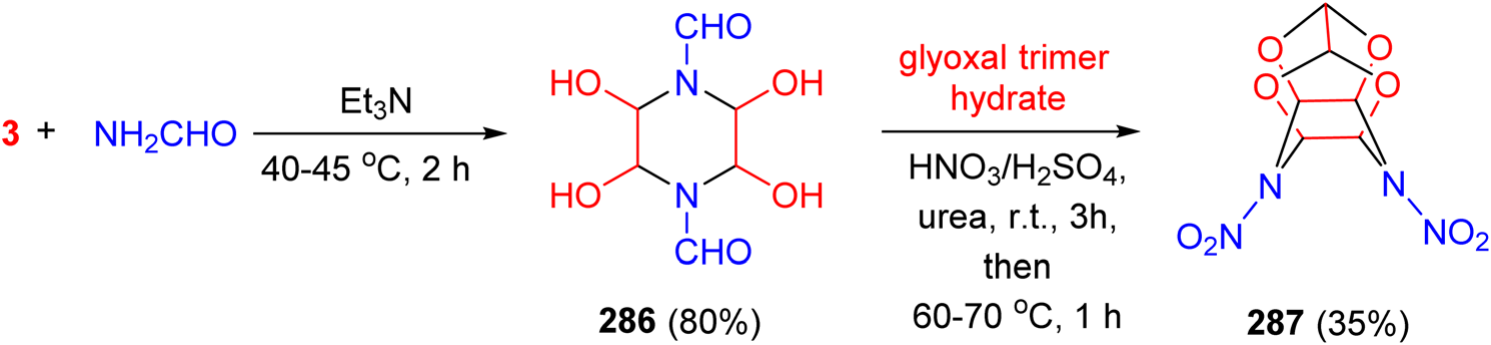
Synthesis of 4,10-dinitro-2,6,8,12-tetraoxa-4,10-diazatetracyclo[5.5.0.0^5,9^0^3,11^]dodecane (287).

1,4-Diformyl-2,3,5,6-tetrahydroxypiperazine (DFTHP) 286 was synthesized in 80% yield by reacting formamide with glyoxal in the presence of Et_3_N at 40–45 °C for 2 h. Next, DFTHP (286) was reacted with glyoxal trimer hydrate under acidic conditions using a mixture of 98% nitric acid, sulfuric acid, and urea. The reaction proceeded at room temperature for 3 h, followed by heating to 65–70 °C for 1 h, giving 4,10-dinitro-2,6,8,12-tetraoxa-4,10-diazatetracyclo[5.5.0.0^5,9^0^3,11^]dodecane (TEX) (287) in 35% yield (Scheme 116).^154,155^

TEX is the second most dense nitramine explosive. It is friction-insensitive, possesses low impact sensitivity (*ca.* 24 J with a BAM impact tester), and exhibits a very large critical diameter >60 mm, which makes this energetic material suitable for large charges such as general-purpose bombs or torpedo warheads. TEX bears a very low shock sensitivity in the NOL-LSGT (50% *ρ* > 6.98 GPa) and possesses excellent compatibility with a wide range of binders and other energetic materials.^156^

**Scheme 117:**
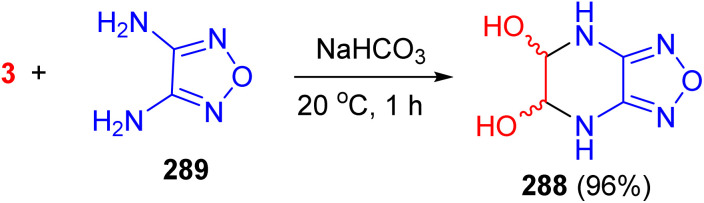
Synthesis oxadiazolo[3,4-*b*]pyrazine 288.

In 2012, Willer and his team described the synthesis of 5,6-dihydroxy-4,5,6,7-tetrahydro[1,2,5]oxadiazolo[3,4-*b*]pyrazine 288 in 96% yield by the reaction of 3,4-diamino[1,2,5]oxadiazole 289 with glyoxal using NaHCO_3_ at 20 °C for 1 h. The DSC thermogram of 288 revealed two endothermic peaks at 113 and 151 °C, indicating a stepwise dehydration process that ultimately produced the target [1,2,5]oxadiazolo[3,4-*b*]pyrazine, in agreement with theoretical predictions (Scheme 117).^157^

**Scheme 118:**
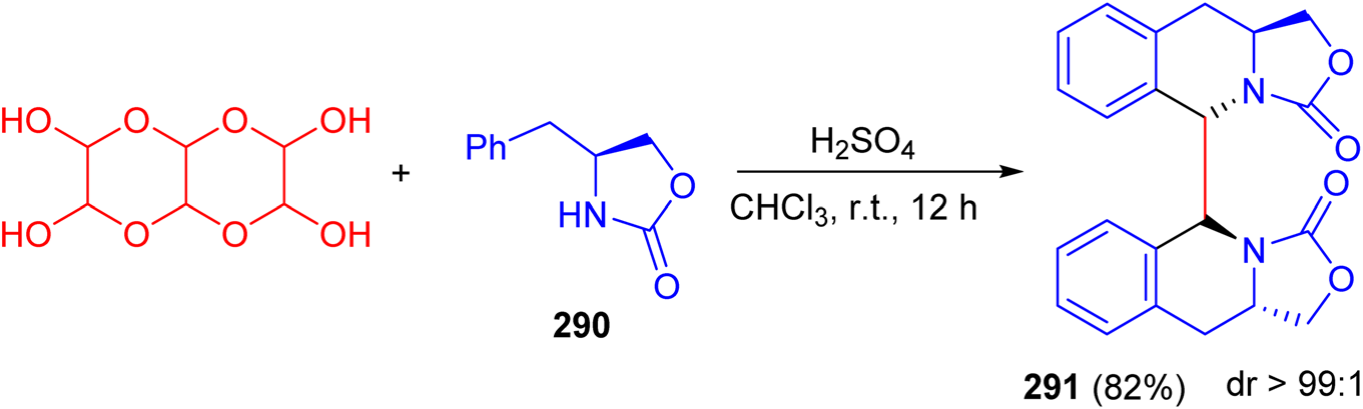
Synthesis of bisoxazolidinone 291.

The reaction of (*S*)-4-benzyloxazolidin-2-one 290 with the hydrate trimer form of glyoxal using H_2_SO_4_ in CHCl_3_ at room temperature for 12 h produced bisoxazolidinone 291 as a pure diastereomer in 82% yield. X-ray crystallographic analysis of 291 revealed a highly constrained dimeric structure in which the aromatic rings are *trans* to each other and the absolute configuration of the two newly created chiral centers is (*S*,*S*). The *trans* arrangement of the two chiral centers within each heterocyclic ring is also evident. However, replacing aqueous glyoxal with its trimeric dihydrate form did not yield the desired product (Scheme 118).^158^

**Scheme 119:**
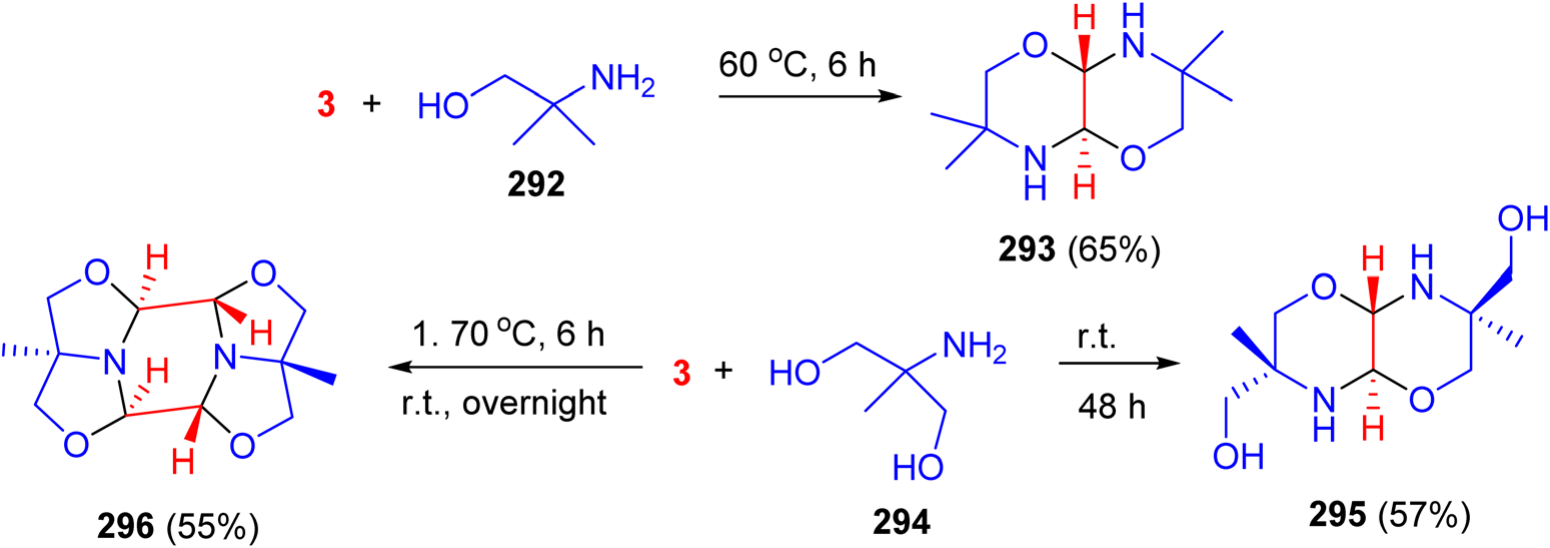
Synthesis of the polycyclic compounds 293, 295 and 296.

In 2015, Peera and his team reported the synthesis of polycyclic compounds through the reaction of amino alcohols with glyoxal. Their study revealed that condensing 2-amino-2-methyl-1-propanol (292) with glyoxal at 60 °C for 6 h gave 3,3,7,7-tetramethyl-octahydro[1,4]oxazino[3,2-*b*][1,4]oxazine (293) in 65% yield. Additionally, when 2-amino-2-methyl-1,3-propanediol (294) was reacted with glyoxal in water at room temperature for 48 h, bis-oxazine 295 was obtained in 57% yield. However, performing the same reaction under elevated temperature conditions (70 °C for 6 h, followed by stirring at room temperature overnight) led to the formation of a more complex polycyclic structure, 296 (55% yield), which was proposed to contain two bis-oxazolidine units. As a result, both 295 and 296 exist as *meso* compounds (Scheme 119).^159^

**Scheme 120:**
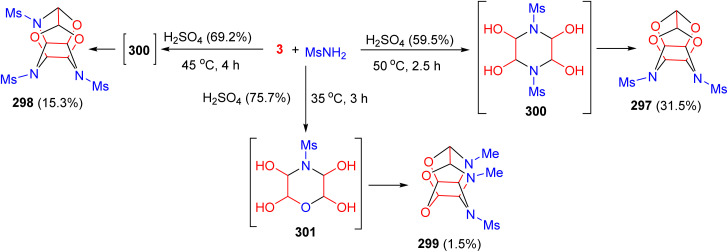
Sulfuric acid catalyzed synthesis of oxaazaisowurtzitanes 297–299.

Sulfuric acid catalyzed condensation of methanesulfonamide (mesylamide) with glyoxal in water at 35–50 °C for 2.5–4 h afforded oxaazaisowurtzitanes 297–299 in 1.5–31.5% yields. The yield of the obtained cage compounds depends on the ratio of initial reagents, their concentration in the reaction mixture, the acidity and temperature of the reaction medium, and also on the order and time of reagents mixing. It is presumed that the formation of compounds 297 and 298 proceeds through the piperazine intermediate 300, and of isowurtzitane 299, through the morpholine intermediate, 2,3,5,6-tetrahydroxy-4-(methanesulfonyl)morpholine 301 (Scheme 120).^160^

**Scheme 121:**
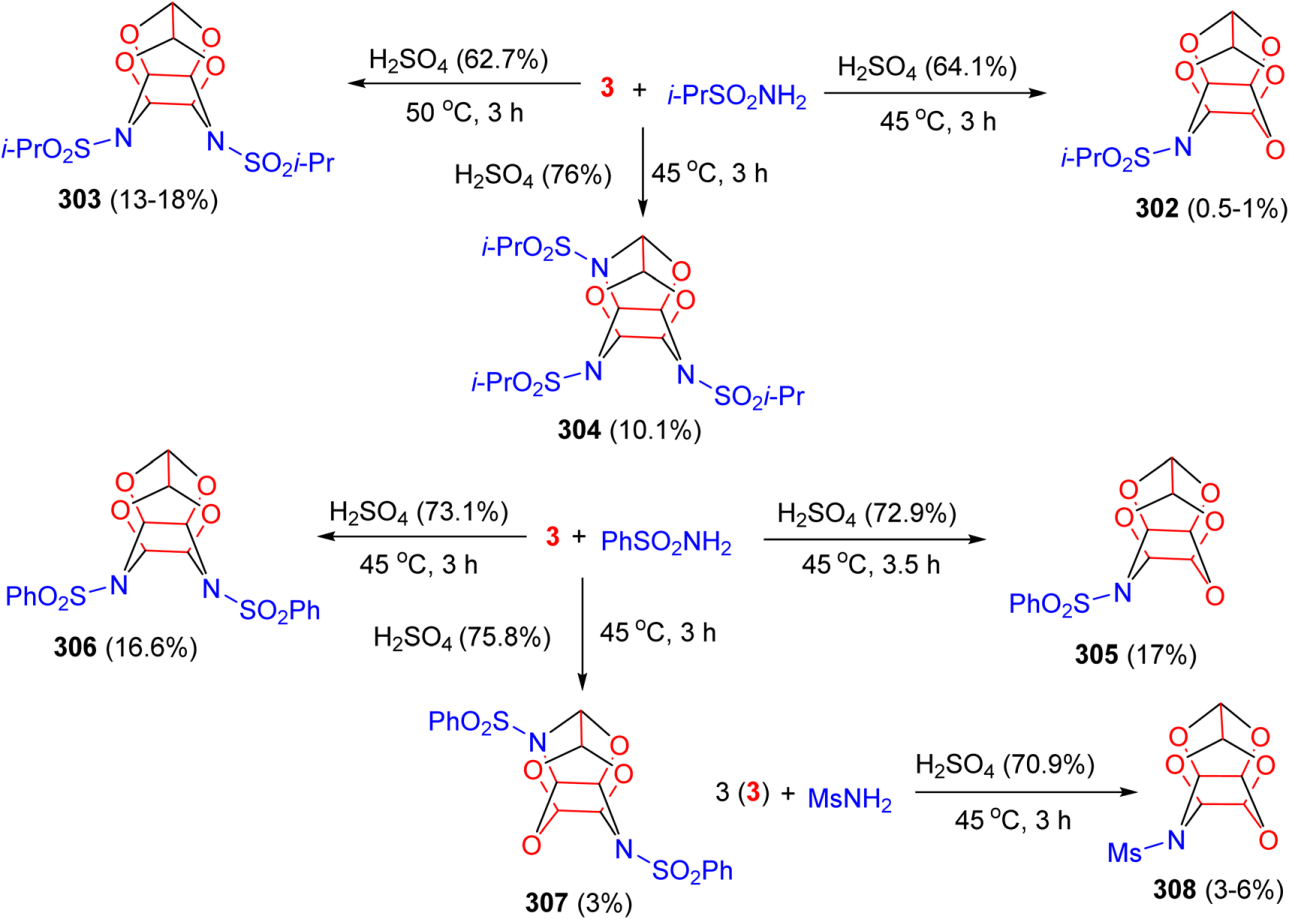
Synthesis of oxaazaisowurtzitane 302–308.

The condensation of propane-2-sulfonamide with glyoxal, catalyzed by H_2_SO_4_ in water at 45–50 °C for 3 h, gave three oxaazaisowurtzitane derivatives 302–304 in 0.5–18% yields. Moreover, the condensation of benzenesulfonamide with glyoxal resulted in three derivatives of oxaazaisowurtzitane 305–307 in 3–17% yields. The yield of the synthesized cage compounds depends on multiple parameters, such as the ratio of starting reagents and their concentration, the reaction medium's acidity and temperature, and the mixing sequence and time. Additionally, when mesylamide was condensed with an excess of glyoxal in the presence of sulfuric acid at 45 °C for 3 h, oxaazaisowurtzitane 308 was obtained in a modest yield of 3–6% (Scheme 121).^161^

**Scheme 122:**
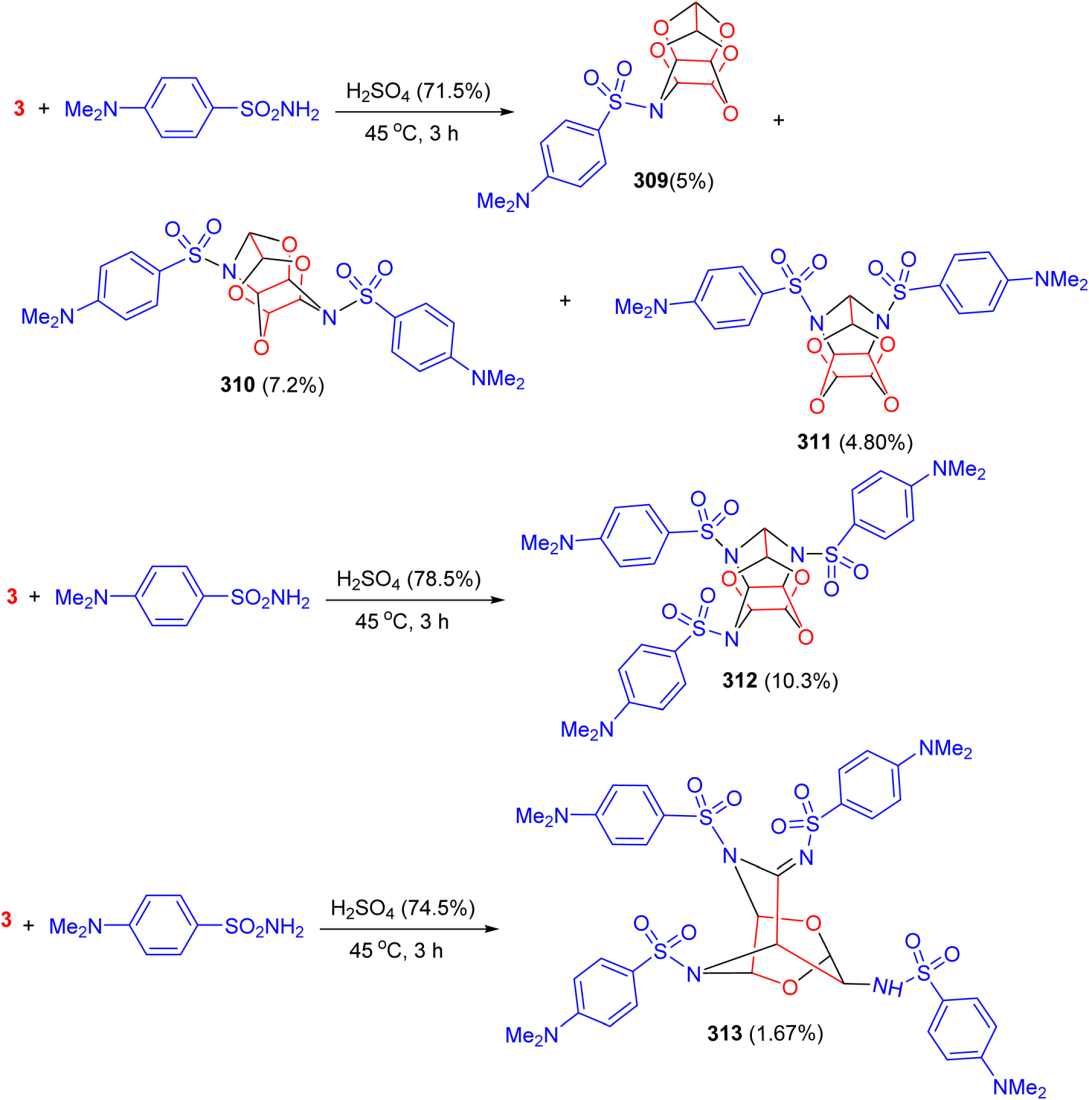
Preparation of *N*-polysulfonyl substituted aza-and oxaazaisowurtzitanes 309–313.

In 2020, Paromov and his team investigated the formation of *N*-polysulfonyl-substituted aza- and oxaazaisowurtzitanes 309–313*via* condensation of 4-dimethylaminobenzenesulfonamide with glyoxal in different ratios in the presence of various concentrations of H_2_SO_4_ as a catalyst in water at 45 °C for 3 h, and discovered new polyheterocyclic caged systems (Scheme 122).^162^

**Scheme 123:**
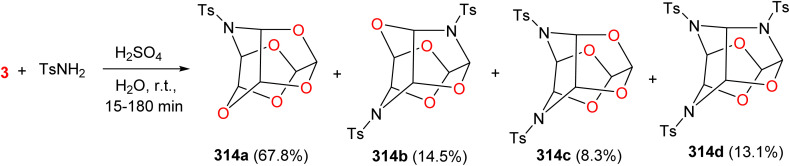
H_2_SO_4_ catalyzed synthesis of the aza- and oxaazaisowurtzitane derivatives 314a–d.

In 2024, Paromov *et al.* conducted a study involving the acid-catalyzed condensation of *p*-toluenesulfonamide with glyoxal using sulfuric acid (H_2_SO_4_) as the catalyst to synthesize a series of aza- and oxaazaisowurtzitane derivatives 314a–d. These compounds are part of a promising platform for high-energy-density materials. The synthesis of compound 314 was accomplished in an aqueous medium at ambient temperature, with reaction times ranging from 15 minutes to 3 h. The formation of the product was investigated by systematically varying key parameters: the stoichiometric ratio of the starting materials, the acidity of the catalyst, and the overall concentration of the reaction mixture. The rate-limiting step in generating oxaazaisowurtzitanes is the formation of strained five-membered rings, specifically oxazolidine and likely imidazolidine. The synthesis rate was found to depend on the specific molecular structure of the oxaazaisowurtzitane. Furthermore, elevated temperatures were shown to dramatically reduce the yield (Scheme 123).^163^

**Scheme 124:**
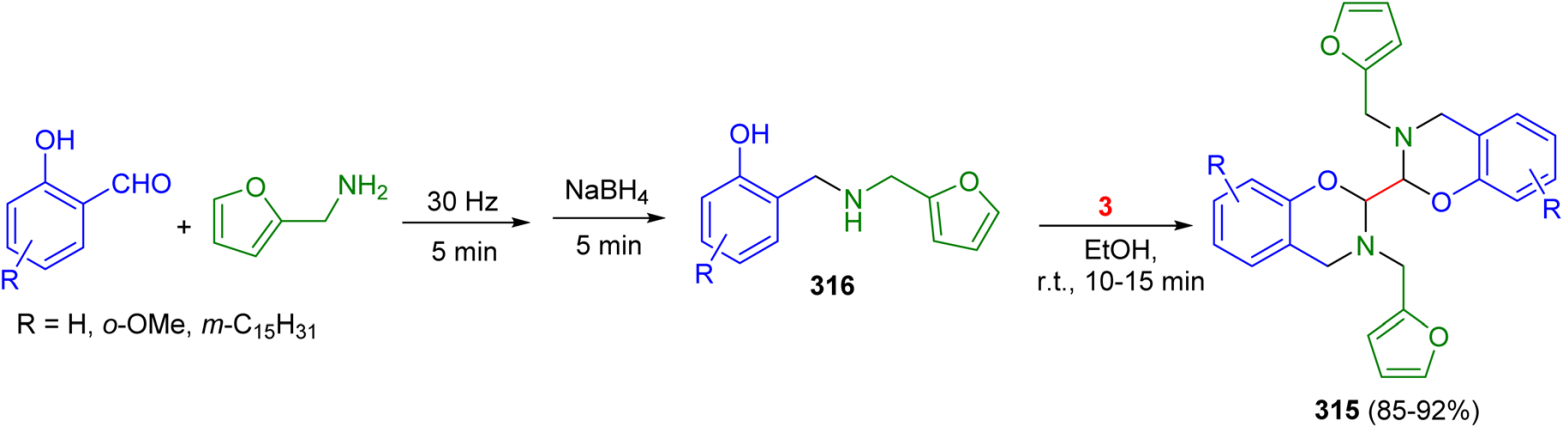
Synthesis of the oxazine-ring-substituted benzoxazine monomers 315.

Recently, a series of oxazine-ring-substituted benzoxazine monomers 315 has been successfully synthesized in 85–92% yields using furan-based aminophenol (AP) derivatives 316. Aminophenol (AP) derivatives were synthesized efficiently *via* a two-step mechanochemical protocol. First, *o*-hydroxybenzaldehydes were condensed with furfurylamine for 5 minutes at 30 Hz to form a Schiff base. This intermediate was then reduced *in situ* using NaBH_4_ for an additional 5 minutes, yielding the target AP derivatives in excellent yields over a total reaction time of 10 minutes. Compounds 315 were synthesized by an atom-efficient, greener process in ethanol with good yields at mild temperatures in 10–15 min. This work demonstrates a proof-of-concept for a highly efficient methodology for formaldehyde replacement in benzoxazine chemistry and holds promise for the exploration of a new platform chemical, glyoxal, toward the next generation of benzoxazine with unique reactivities (Scheme 124).^164^

### 4.6. Synthesis of the other heterocyclic and carbocyclic compounds

**Scheme 125:**
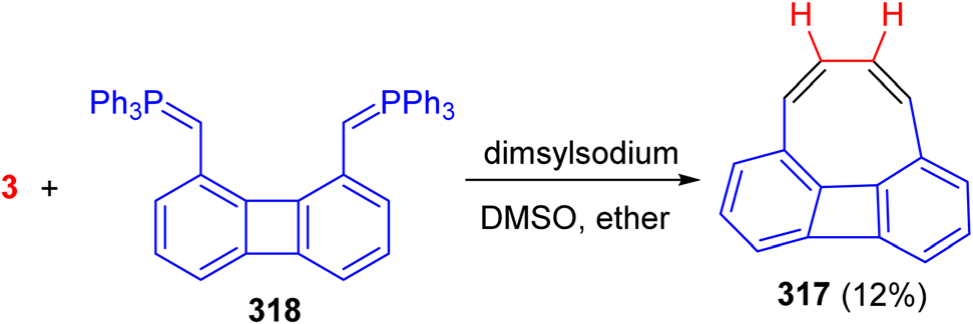
Synthesis of bis-ylide 317.

A freshly prepared ether solution of glyoxal, when treated with a bis-ylide 317 generated from 1,8-bis(bromomethyl)biphenylenetriphenylphosphonium salt and dimsyl sodium] afforded a complex mixture that contains 12% of the interesting cycloocta[def]biphenylene 318 using dimsyl sodium in DMSO (Scheme 125).^165^

**Scheme 126:**
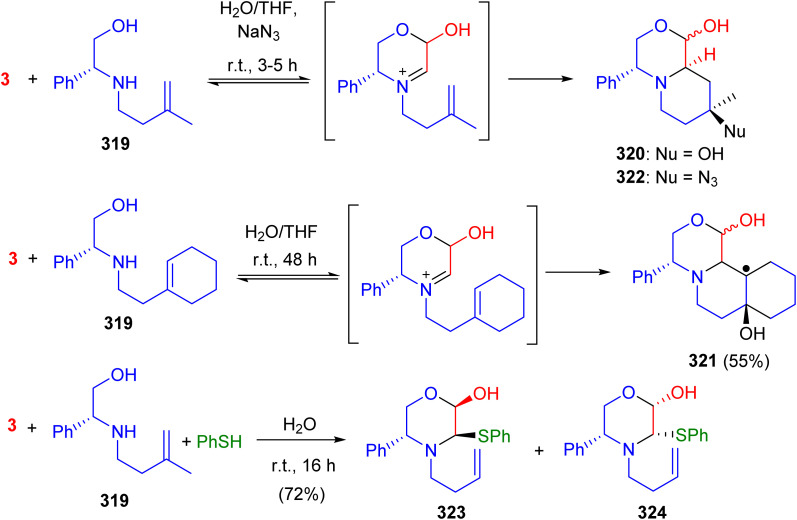
Synthesis of the mono/bi/tri-cyclic compounds 320–324.

Agami and co-workers found that the condensation of chiral *N*-homoallyl β-amino alcohols 319 with glyoxal produces iminium ions, which are cyclised with complete stereoselectivity. β-Amino alcohols 319 react with glyoxal in a water-tetrahydrofuran solution at room temperature to afford bicyclic compounds 320 and 321 (respective times: 5 and 48 h and yields: 72 and 55%). When an excess of sodium azide was present in the reaction medium, substrate 319 yielded a mixture of products 320 and 322 in a l : 5 respective ratio after 3 h at room temperature. Additionally, the diastereomeric mixture of amino thioethers 323 and 324 in a 3 : 1 respective ratio, was prepared in 72% yield from the one-pot condensation of compound 319, glyoxal and thiophenol in aqueous solution at room temperature for 16 h (Scheme 126).^166^

**Scheme 127:**
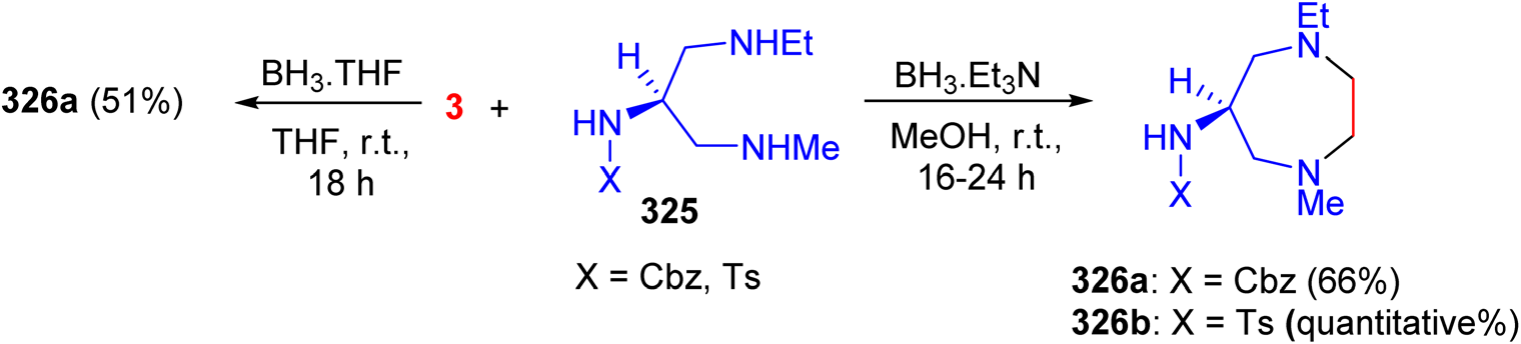
Preparation of the hexahydro-1,4-diazepine ring 326a–b.

Hirokawa and his colleagues developed the cyclization of 1,2,3-trisubstituted aminopropane 325 into the hexahydro-1,4-diazepine ring 326a–b by reaction with glyoxal in the presence of the BH_3_·Et_3_N complex in MeOH at room temperature for 16–24 h. Also, compound 326a was synthesized in 51% yield by using the BH_3_·THF complex in THF at room temperature for 18 h (Scheme 127).^167^

**Scheme 128:**
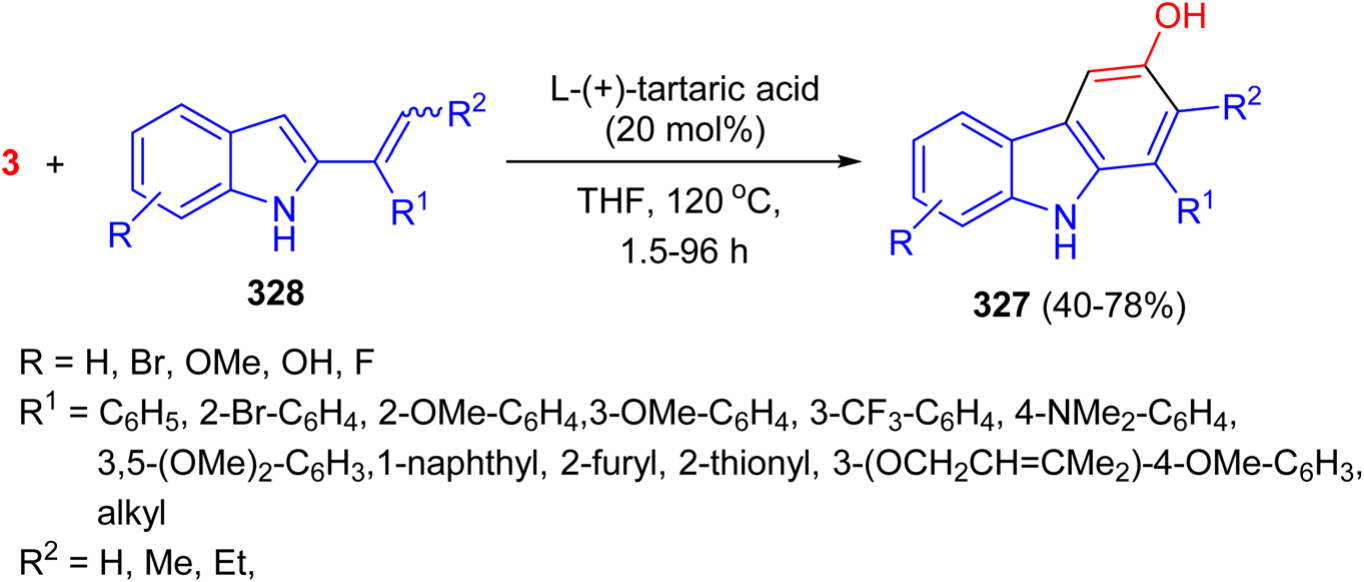
Synthesis of 3-hydroxy carbazoles 327.

In 2022, Maji and his colleagues developed the synthesis of 3-hydroxy carbazoles 327 in 40–78% yields by the reaction of 2-alkenyl indole 328 with glyoxal in the presence of l-(+)-tartaric acid in THF at 120 °C for 1.5–96 h. It should be noted that, in the case of 2-alkenyl indole having an aryl and heteroaryl functionality at the R^1^ position, both *E*- and *Z*-isomers reacted quite efficiently, producing the desired 3-hydroxy carbazole, whereas in the case of 2-alkenyl indole, having an alkyl functionality at the R^1^ position, the *E*-isomer reacted more efficiently compared to the respective *Z*-isomer. The reaction showed a broad substrate scope and hence provides an opportunity to deliver various unnatural carbazole scaffolds that structurally resemble their natural counterpart (Scheme 128).^168^

Owing to its high reactivity, glyoxal can participate in a wide range of reactions, leading to the formation of diverse products, particularly novel heterocyclic compounds that may not be readily anticipated. Consequently, these products may exhibit broad industrial applications, especially in the pharmaceutical field. In addition, many of these compounds have potential utility as ligands. To date, heterocyclic compounds synthesized using glyoxal have received limited attention with respect to their medicinal properties, and it is anticipated that greater focus will be devoted to this area in the future. Furthermore, there is a clear need to explore new synthetic routes, investigate physicochemical and biological properties, and identify new applications of novel heterocyclic compounds, particularly in pharmacology. Accordingly, current and future research trends in glyoxal-derived compounds are expected to focus on expanding their applications across various fields.

## 5. Conclusions

This review provides a comprehensive overview of advancements in synthesizing diverse molecular frameworks from glyoxal. It details synthetic methodologies, from one-pot multicomponent to sequential reactions, employing diverse catalysts and conditions to construct a wide array of structures, including five-membered heterocycles (*e.g.*, as imidazolidinones, imidazolidinthiones, glycolurils, imidazolidines, imidazo[4,5-*d*]imidazoles, imidazoles, imidazolium zwitterions, bis-imidazoles, benzofurans and naphthofurans, six-membered heterocycles (*e.g.*, piperazines, quinoxalines and triazines), aromatic compounds, fused heterocycles, polyaza polycyclic compounds, polyoxa polycyclic compounds and polyaza–polyoxa polycyclic compounds. The high reactivity of glyoxal makes it a valuable substrate in synthetic organic chemistry, particularly for synthesizing heterocyclic scaffolds. This review will help synthetic chemists update their knowledge on recent developments in this field.

## Author contributions

Writing-original draft preparation: Abolfazl Olyaei; writing-review and editing: Abolfazl Olyaei and Mahdieh Sadeghpour; and supervision: Mahdieh Sadeghpour. All authors have read and agreed to the published version of the manuscript.

## Conflicts of interest

The authors declare no conflicts of interest.

## Data Availability

No primary research results, software, or code have been included, and no new data were generated or analyzed as part of this review.
